# Synthesis, structure and *in vitro* antiproliferative effects of alkyne-linked 1,2,4-thiadiazole hybrids including erlotinib- and ferrocene-containing derivatives†

**DOI:** 10.1039/d1ra05095h

**Published:** 2021-08-25

**Authors:** Mohammed Boulhaoua, Tibor Pasinszki, Ana Torvisco, Rita Oláh-Szabó, Szilvia Bősze, Antal Csámpai

**Affiliations:** ELTE Eötvös Loránd University, Institute of Chemistry, Department of Inorganic Chemistry H-1117 Budapest Hungary; Fiji National University, College of Engineering Science and Technology, Department of Chemistry P.O.Box 3722, Samabula Suva Fiji tibor.pasinszki@fnu.ac.fj; Graz University of Technology, Institute of Inorganic Chemistry Stremayrgasse 9/V 8010 Graz Austria; MTA-ELTE Research Group of Peptide Chemistry Pázmány P. sétány 1/A H-1117 Budapest Hungary; ELTE Eötvös Loránd University, Institute of Chemistry, Department of Organic Chemistry H-1117 Budapest Hungary antal.csampai@ttk.elte.hu

## Abstract

Chemotherapy is an indispensable tool to treat cancer, therefore, the development of new drugs that can treat cancer with minimal side effects and lead to more favorable prognoses is of crucial importance. A series of eleven novel 1,2,4-thiadiazoles bearing erlotinib (a known anticancer agent), phenylethynyl, ferrocenyl, and/or ferrocenethynyl moieties were synthesized in this work and characterized by NMR, IR and mass spectroscopies. The solid-phase structures were determined by single-crystal X-ray diffraction. Partial isomerisation of bis(erlotinib)-1,2,4-thiadiazole into its 1,3,4-thiadiazole isomer, leading to the isolation of a 3 : 2 isomer mixture, was observed and a plausible mechanism for isomerisation is suggested. The *in vitro* cytostatic effect and the long-term cytotoxicity of these thiadiazole-hybrids, as well as that of erlotinib, 3,5-dichloro-1,2,4-thiadiazole and 3,5-diiodo-1,2,4-thiadiazole were investigated against A2058 human melanoma, HepG2 human hepatocellular carcinoma, U87 human glioma, A431 human epidermoid carcinoma, and PC-3 human prostatic adenocarcinoma cell lines. Interestingly, erlotinib did not exhibit a significant cytostatic effect against these cancer cell lines. 1,2,4-Thiadiazole hybrids bearing one erlotinib moiety or both an iodine and a ferrocenethynyl group, as well as 3,5-diiodo-1,2,4-thiadiazole demonstrated good to moderate cytostatic effects. Among the synthesized 1,2,4-thiadiazole hybrids, the isomer mixture of bis-erlotinib substituted 1,2,4- and 1,3,4-thiadiazoles showed the most potent activity. This isomer mixture was proven to be the most effective in long-term cytotoxicity, too. 3,5-Diiodo-1,2,4-thiadiazole and its hybrid with one erlotinib fragment were also highly active against A431 and PC-3 proliferation. These novel compounds may serve as new leads for further study of their antiproliferative properties.

## Abstract 2

Fourteen 1,2,4-thiadiazole derivatives were synthesized and structurally characterized; their *in vitro* antiproliferative activities were evaluated on four tumorous cell lines (U87, A2058, A431, and HepG2).

## Graphical abstract

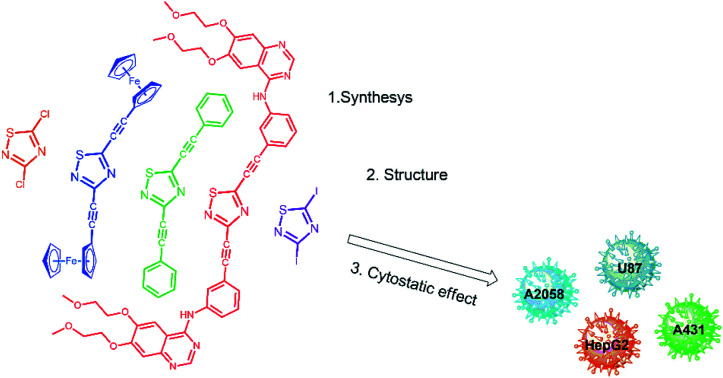

## Introduction

The various types of cancer are highly devastating diseases worldwide, in most cases with poor prognosis and low survival rates.^1,2^ It is no doubt that chemotherapy is one of the most indispensable tools for the treatment of malignancies. However, the clinical efficacy of most anticancer chemotherapies is substantially decreased by a variety of factors including multidrug resistance (MDR)^3,4^ and severe adverse effects that strongly make it necessary to develop more potent novel drugs with enhanced activity and selectivity towards tumours. One of the most promising new strategies in chemotherapy is based on the design and synthesis of hybrid compounds, by coupling different pharmacophore fragments.^5–7^ Such hybrid drugs having more than one molecular target at the cellular level can be considered to be anticancer agents of enhanced efficiency triggering cell death along multiplied pathways.

**Scheme 1:**
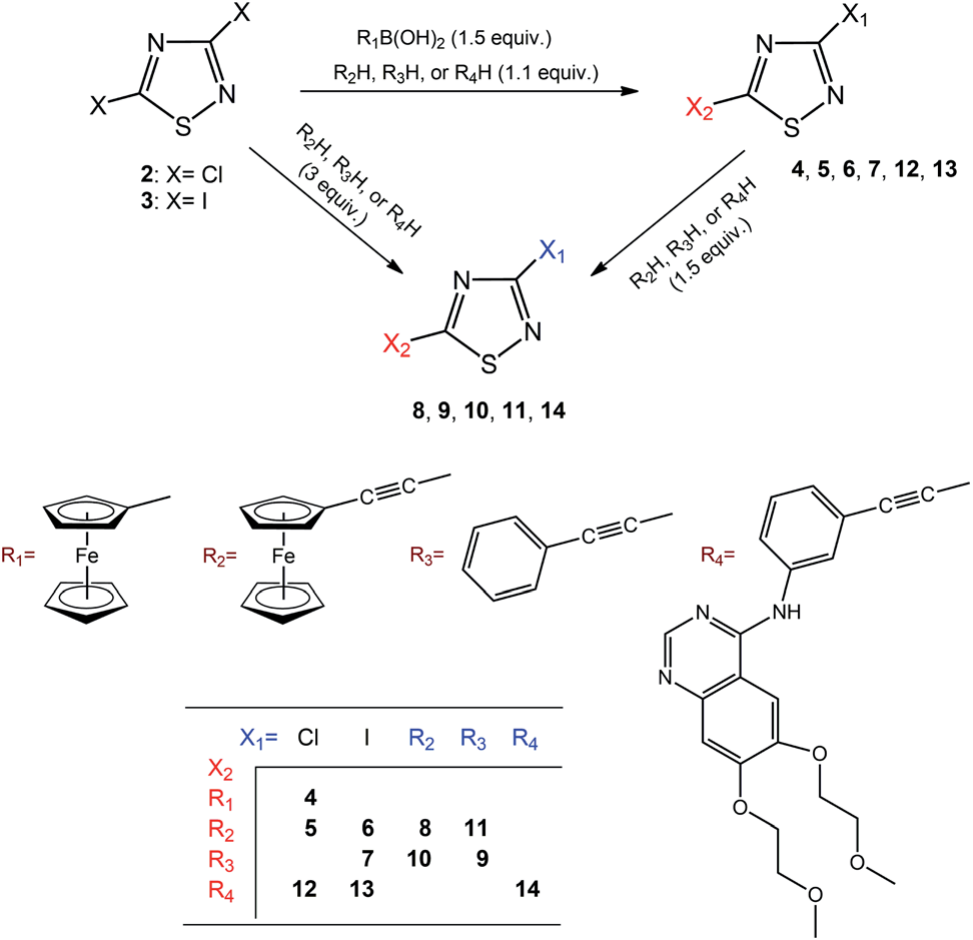
Synthesis and labelling of compounds.

**Scheme 2:**
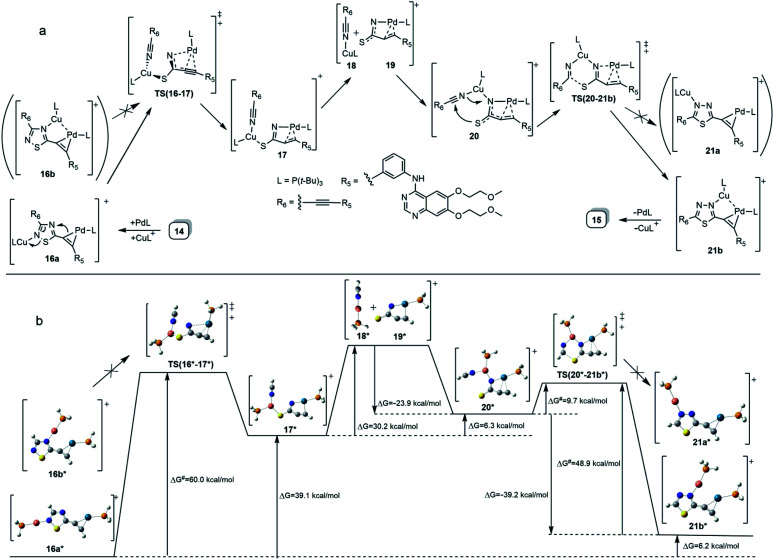
(a) Proposed mechanism for a bimetal-catalysed isomerization 14 → 15 taking place under the conditions of Sonogashira coupling reaction. (b) DFT-based representation of the mechanism involving intermediates and transition states (TS) of simplified structures along with the activation barriers and the relative energetics of the particular steps.

Hybrid anticancer agents possess real potential to overcome certain disadvantages of single cancer drugs, including MDR and adverse effects; therefore, based on this promising strategy, the aim of the present work was to develop novel anticancer agents for the potential treatment of human glioblastoma,^8^ melanoma,^9^ non-melanoma skin carcinoma,^10,11^ hepatocarcinoma^12^ and prostatic adenocarcinoma^13^ by coupling the pharmacophore 1,2,4-thiadiazole moiety to ethynylferrocene and to the known anticancer agent erlotinib, (*N*-(3-ethynylphenyl)-6,7-bis(2-methoxyethoxy)quinazolin-4-amine) (1). Thiadiazoles are versatile synthetic scaffolds possessing a wide range of biological activities.^14–16^ 1,2,4-Thiadiazole derivatives have been shown to exhibit anticancer activity against *e.g.*, breast, lung, colon, and ovarian cancerous cells.^15,16^ Ferrocene-based compounds have been reported to be active against several cancer cell lines, including *e.g.*, breast, lung, ovarian, melanoma, glioblastoma, and colon cancer.^17–19^ Erlotinib is an approved anticancer drug, and currently used for the treatment of locally advanced or metastatic non-small-cell lung cancer and pancreatic cancer.^20,21^ In this context, one of the aims of the present work was an *in vitro* test of the antiproliferative activity of 1 on human malignant cell lines A2058 (melanoma), A431 (epidermoid carcinoma), U87 (glioma), HepG2 (hepatocellular carcinoma), and PC-3 (prostatic adenocarcinoma) followed by an attempt to produce novel erlotinib-containing hybrids of enhanced efficiency. In this regard, prompted by the aforementioned well-documented activity profile of anticancer 1,2,4-thiadiazoles described to date and utilising Sonogashira reaction as an obvious feasible method, 1 was coupled with 3,5-dihalo-substituted 1,2,4-thiadiazoles (dichloro: 2, diiodo: 3) affording novel molecular hybrids. In order to estimate the contribution of the bulky, yet highly polar and functionalised erlotinib fragment to cytotoxicity in the design of additional hybrids, the quinazolinamine fragments were simplified to phenylethynyl groups, which were then further modified by replacing phenyl substituent(s) with three-dimensional and redox-active ferrocenyl group(s) of enhanced lipophilicity. Accordingly, in this paper, we report the synthesis and structural characterisation of a selection of novel thiadiazole-hybrids (4–15: Schemes 1 and 2) along with their evaluation in antiproliferative studies carried out *in vitro* on A2058, A431, U87, HepG2 and PC-3 cells using 1, 2 and 3, as controls.

## Results and discussion

### Synthesis and identification of compounds

**Fig. 1:**
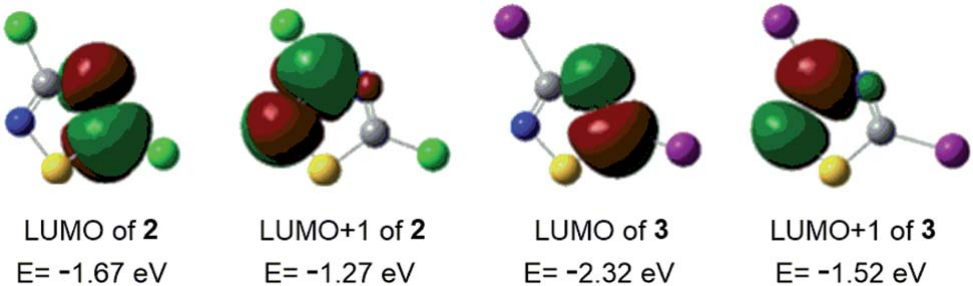
Acceptor orbitals of lowest energy levels in 3,5-dihalo-1,2,4-thiadiazoles 2 and 3 obtained by B3PW91 DFT functional^22^ using DGTZVP basis set.^23^

Considering the ethynyl functional group of 1, ethynylferrocene and phenylacetylene, it seemed to be straightforward to couple these compounds with 3,5-dihalo-1,2,4-thiadiazoles, 2 and 3, using Sonogashira-type cross-coupling reactions (Scheme 1; note that the bromo-derivative of these thiadiazoles is not known yet). Sonogashira-coupling was indeed a good choice, however, the product yield was very sensitive to reaction conditions, namely to solvent, base, catalyst, and temperature (see Tables S1 and S2, ESI†). The best results for coupling ethynylferrocene and phenylacetylene to thiadiazole were obtained by using toluene as the solvent, diisopropylamine (DIPA) as the base, and PdCl_2_(PPh_3_)_2_/CuI as the catalyst at a slightly elevated temperature of 50 °C. However, the combination of DMF, K_3_PO_4_, and Pd[P(*t*-Bu)_3_]_2_/CuI at 80 °C provided the most efficient conditions for the coupling with erlotinib. Using 1.1 or 3 equivalents of the alkyne component (1, ethynylferrocene or phenylacetylene) under optimised conditions, the cross-coupling reactions led smoothly and selectively to thiadiazole products monosubstituted at position five (C5) or to the disubstituted products, respectively, in isolated yields varied between 66 and 87%. In the subsequent step the monosubstituted 1,2,4-thiadiazoles were then converted into the target disubstituted products (Scheme 1). The selectivity of the coupling reaction in the first step allowed the synthesis of thiadiazole-hybrids with two different substituents by selecting the sequence of the introduction of the selected alkyne component. The regioselectivity of the first reaction step can be explained by the lower electron density of the thiadiazole ring at C5 and thus higher reactivity with nucleophiles, compared to position C3.^14^ This regioselectivity was unambiguously confirmed by the MO analysis of 2 and 3 disclosing LUMO concentrated at C5–N4 region and LUMO+1 delocalised over C3–N2 region in both models (Fig. 1). Moreover, the energetic data calculated for these orbitals are also in good agreement with the experimentally observed relative reactivity of 3 and 2, indicating that the diiodo derivative is more reactive than its dichloro-substituted counterpart. To investigate the effect of the ethynyl spacer between the thiadiazole moiety and ferrocenyl group, this latter was also directly linked to 2 applying Suzuki-type coupling. Reaction conditions were studied and good yield was obtained using dioxane, K_2_CO_3_, and Pd(OAc)_2_/PPh_3_ at reflux conditions (see Table S3, ESI†). Since the removal of the ethynyl spacer has not resulted in improved antiproliferative properties, this research direction has not been further investigated. All synthesized compounds have been unambiguously identified and characterized by mass (Fig. S1–S9, ESI†), IR (Fig. S10†), and NMR spectroscopies. Molecular masses were determined by ESI-MS, and IR spectroscopy provided a characteristic fingerprint for the ethynyl group(s) in the 2200–2227 cm^−1^ spectral region. The constitution of all newly synthesized thiadiazole-hybrids was confirmed by ^1^H and ^13^C NMR data (Fig. S17–S30, ESI†) assigned on the basis of connectivities disclosed by 2D-HSQC- and HMBC measurements (Fig. S31–S37, ESI†). In addition, the solid phase structures of hybrids 4, 5, 6, 8, 10 and 11 have also been determined by single-crystal X-ray diffraction as discussed later in details.

It is of interest that Sonogashira coupling reactions aiming at the synthesis of bis-erlotinib derivative 14 led to the isolation of an approximately 3 : 2 mixture of the targeted product and its isomer 15 featuring a [1,3,4]thiadiazole-centered symmetrical constitution (Scheme 2), as detected and identified by extended NMR studies including 2D-HSQC and 2D-HMBC measurements.

**Fig. 2:**
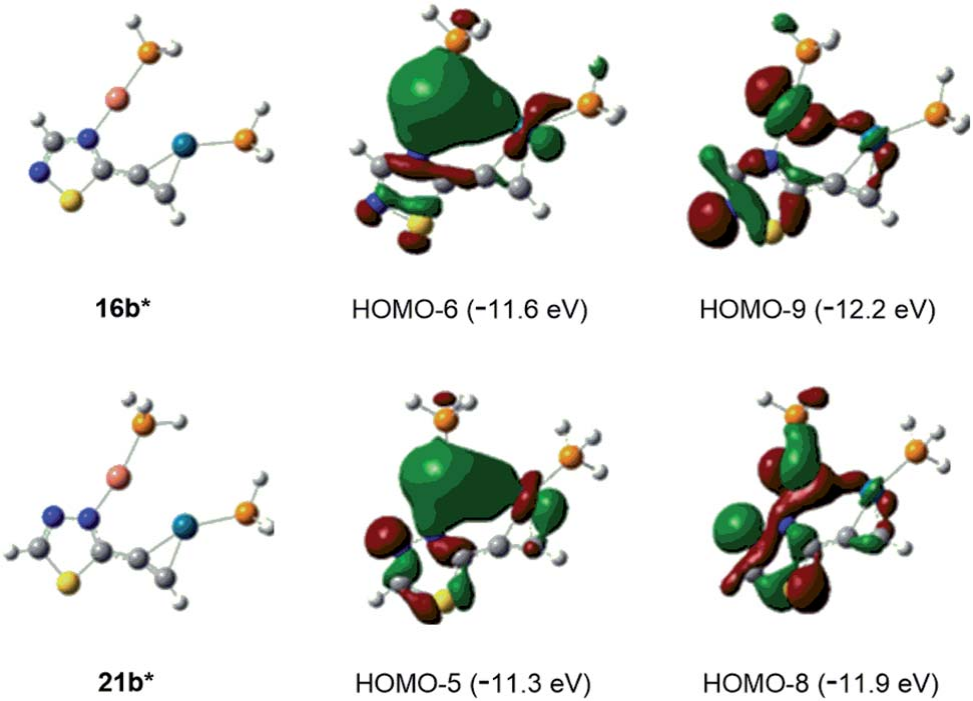
Interactions between Cu(i) and Pd(0) centers in simplified bimetal model complexes 16b* and 21b* represented by the delocalisation of bonding orbital pairs HOMO-6/HOMO-9 and HOMO-5/HOMO-8, respectively.

The formation of 15 can be interpreted in terms of the partial isomerisation of 14, the primary product of the Sonogashira reaction when it was conducted at 95 °C for prolonged reaction times (for 15 h by Procedure A and for 20 h by Procedure B) representing harsher conditions than those employed for the other conversions investigated in our research that were conducted at 50–80 °C for 6–12 h (see: Experimental). The mechanism proposed by us for transformation 14 → 15 involves the initial π-complexation of a Pd(0) species with the alkyne residue at C5-position and a concomitant coordination of a Cu(i) species to the N2 ring atom to construct bimetallic cationic complex 16a (Scheme 2a). By means of the coordination of the Cu(i) centre to the proximal S1 atom and the development of a bonding interaction between alkyne-coordinated Pd(0) and the skeletal N4 atom in 16a, the two metal centres promote the fission of the S1–N2 and C3–N4 bonds proceeding *via* transition state TS(16-17) to generate a five-membered palladacycle intermediate with pending tricoordinated copper(i)-centre (17). In the subsequent steps involving separated intermediates 18 and 19, complex 17 is supposed to isomerize to complex 20 featuring a N–Cu–N coordination mode. To terminate the rearrangement process, 20 undergoes electrocyclisation through a transition state TS(20-21b), with a quasi-six membered ring, finally constructing 21b stabilized by a Cu(i)⋯Pd(0) interaction as evidenced by DFT calculations performed on simplified models (Fig. 2). Regarding the initial elementary step it must be noted here that – according to the aforementioned theoretical studies – the cleavage of S1–N2 and C3–N4 bonds in regioisomer complex 16b, stabilized by Cu(i)⋯Pd(0) interaction, is not feasible to advance the process towards a complete ring transformation. The presented view about the mechanism gained support from the results of DFT modelling carried out at B3PW91/DGTZVP level of theory for the simplified structures of the assumed intermediates (16a,b*, 17*, 18*, 19*, 20*, 21a,b*) and transition states [TS(16*-17*) and TS(20*-21b*)] comprising single ethynyl group and appropriately coordinated neutral and cationic metal-containing simplified fragments PdPH_3_ and CuPH_3_, respectively (Scheme 2b). The intermediates and transition states were identified as local minima and first order saddle points, respectively, on the potential energy surface (PES). Transition states were localized by QST2 method.^24^ The energetic profile of the overall transformation was characterized by the changes in Gibbs free energy (*G*) accompanying the assumed elementary steps and the activation barriers of the ring opening and ring closing processes. The free energy values of optimised structures were obtained by correcting the computed total energy with zero-point vibrational energy (ZPE) and thermal corrections calculated at the same level. In the initial stage of the conversion, the fission of the 1,2,4-thiadiazole ring was disclosed as the rate-limiting endothermic elementary step (Δ*G* = +39.1 kcal mol^−1^) proceeding *via* a high barrier (Δ*G*^‡^ = 60.0 kcal mol^−1^) followed by an endothermic isomerization of the palladacycle intermediate (17* → 20*) taking place by the copper-centred decoordination–coordination sequence *via* separated Cu(i)- and Pd(0) fragments 18* and 19*. Finally, 20* was identified as the intermediate which can be connected by transition state TS(20*-21b*) exclusively with 21b* in accord with qualitative structural considerations, in the 1,3,4-thiadiazole-forming cyclization. The multistep isomerization of bimetal complex 16a* into 21b* is an endothermic process as indicated by the change in the free energy calculated for the overall conversion (Δ*G* = +6.2 kcal mol^−1^). Moreover, the change in the Gibbs free energy [Δ*G*(15*-14*) = +14.5 kcal mol^−1^] calculated for vacuum by modelling simplified metal-free isomer pair 2-ethynyl-1,3,4-thiadiazole(15*)/5-ethynyl-1,2,4-thiadiazole (14*) would suggest that – in general – the transformation of 5-alkynyl-substituted 1,2,4-thiadiazoles into the 1,3,4-thiadiazole counterpart is not a feasible process however, the relative energetics of structures 14 and 15 and their appropriate metal complexes under real experimental conditions with bulky phosphine ligand [P(*t*-Bu)_3_] of outstanding donor strength and dimethylformamide as solvent of significant coordination- and solvation ability, might significantly differ from the calculated values allowing the development of an equilibrium system containing 15 as the minor component. In accord with this view, upon further prolongation of the reaction time (24 h) practically no change in the isomer ratio was discernible in the isolated mixture of products 14 and 15.

Finally, the abovementioned Cu(i)⋯Pd(0) contact was disclosed by MO analysis of the simplified models 16b* and 21b*. The enhanced stability of these complexes relative to their coordination isomers 16a* [Δ*G*(16a*–16b*) = +5.0 kcal mol^−1^] and 21a* [Δ*G*(21a*–21b*) = +4.2 kcal mol^−1^], respectively, can be attributed to this type of interaction which is demonstrated by the delocalisation of two–two bonding orbitals in between the metal centres (Fig. 2). Our attempts to separate 14 from 15 was not successful, therefore the mixture 14/15 was used in biological studies.

### Crystal structure of ferrocenethynyl- and phenylethynyl-hybrids

**Fig. 3:**
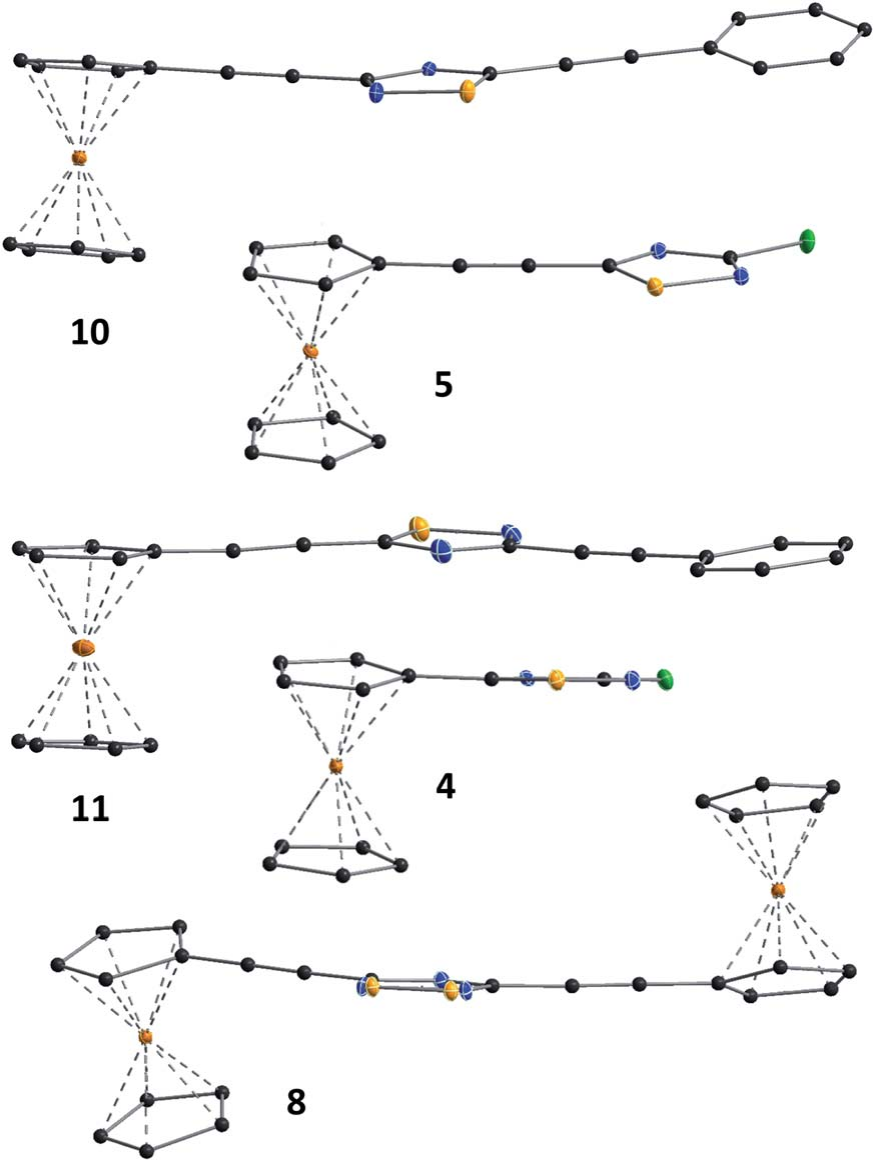
Solid state structure of 4, 5, 8, 10 and 11 established by single crystal X-ray diffraction. All atoms are shown as 30% shaded ellipsoids (C: black, N: blue, S: yellow, Cl: green, Fe: orange).

**Fig. 4:**
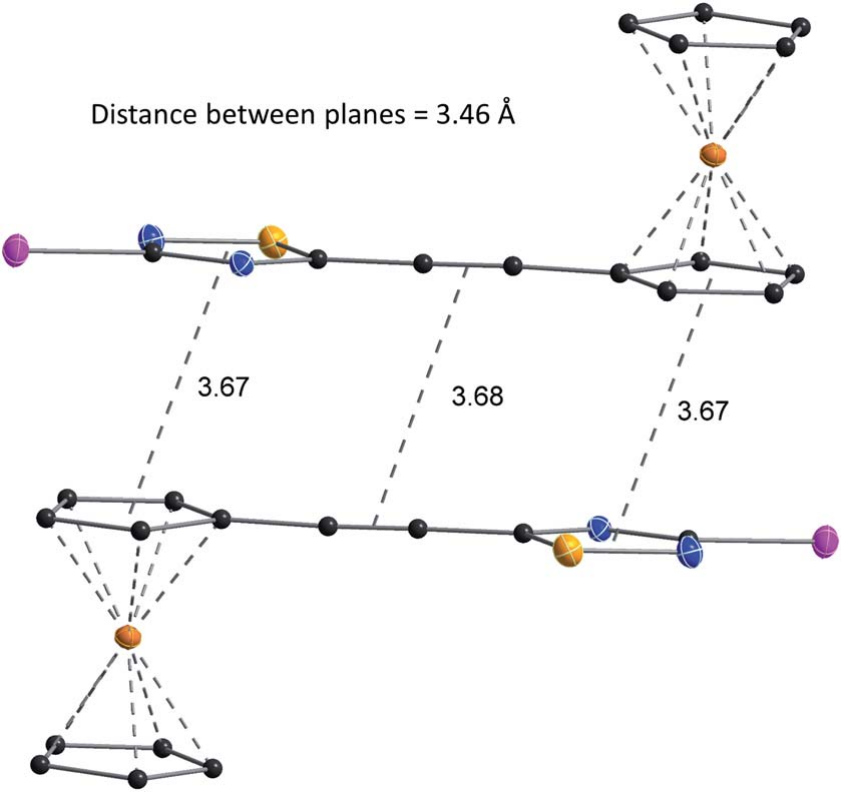
Structure and packing of 6. All atoms are shown as 30% shaded ellipsoids (C: black, N: blue, S: yellow, I: violet, Fe: orange).

The structures of compounds 4, 5, 6, 8, 10, and 11 in the solid phase were determined by solving X-ray diffraction data collected from single crystals. We note that our attempts to grow X-ray quality single crystals from erlotinib derivatives were to no avail. The structure of 7 was published in our previous paper.^25^ The crystal structures of 4, 5, 6, 8, 10, and 11 are shown in Fig. 3 and 4 with geometric parameters listed in Tables S4–S9, ESI.† Compound 5 crystallizes in the triclinic space group *P*1̄, all others in the monoclinic space group *P*2_1_/*c*. All investigated hybrids exhibit a quasi-planar arrangement of the thiadiazole and connected phenyl or cyclopentadienyl rings; small deviations can be explained by solid-phase effects. A characteristic motif of the crystal packing is the formation of molecular dimers, by weak π⋯π interactions between aromatic rings of oppositely oriented molecules (see Fig. 4), and their arrangement into columns in the crystal (Fig. S11–S16, ESI†). Intermolecular interactions in crystals, in general, are weak as indicated by the relatively large distance between ring centroids and interatomic distances (Tables S10–S12, ESI†).

### Antiproliferative activity of compounds 1–13 and 3/2 mixture of 14/15

The effects of 1–13 and 14/15 on the growth of five human cancer cell lines were investigated using three different protocols. Protocol 1 has involved the treatment of cells with 1–14/15 for 20 h, consecutive washing, and incubating cells for an additional 72 h. Half maximal inhibitory concentration (IC_50_) values are presented in Tables 1 and 2. Interestingly, 1 has not shown a significant cytostatic effect on U87, A2058, A431, HepG2, and PC-3 cells (IC_50_ > 50 μM). Linking 1 to the 1,2,4-thiadiazole ring, however, resulted in enhanced cytostatic effect; compound mixture 14/15 comprising the 1,2,4- and 1,3,4-thiadiazole isomers with two erlotinib moieties exhibited a significant anti-tumour effect on all five cell lines (IC_50_ < 2 μM in case of U87, A2058, HepG2, and PC-3; IC_50_ < 6 μM in case of A431). PC-3 cells were the most sensitive to 14/15 (IC_50_ = 0.4 μM). Iodine substituent on the 1,2,4-thiadiazole ring was found to be effective in increasing cytostatic effect; the diiodo-1,2,4-thiadiazole (3) was observed to be equally effective against A431 cells as its mono and di-substituted erlotinib derivatives (13 and 14/15). 3 and all erlotinib-hybrids were effective against PC-3. The linking of ferrocene, ethynylferrocene or ethynylbenzene to the thiadiazole frame has not resulted in a marked anti-tumour effect, namely with an IC_50_ value lower than 10 μM (Table 1), however, several compounds exhibited notable cytostatic effect with IC_50_ values between 10 and 30 μM (see Table 1).

**Table 1:** *In vitro* cytostatic effect (half maximal inhibitory concentration (IC_50_) values) of compounds 1–14/15 on U87, A2058, A431 and HepG2 cell lines

Label	U87	A2058	A431	HepG2
**IC**_**50**_ **(μM) 20 h treatment + 72 h incubation**				
1	>100	>100	78.1 ± 14.4	>100
2	>50	11.3 ± 1.0	28.6 ± 5.7	>50
3	15.3 ± 0.6	20.2 ± 7.9	5.5 ± 2.9	13.0 ± 1.9
4	>50	>50	>50	>50
5	>50	>50	>50	>50
6	18.2 ± 6.1	17.1 ± 6.5	22.0 ± 7.9	13.9 ± 3.3
7	>50	>50	19.5 ± 9.3	>50
8	>50	>50	>50	>50
9	>50	>50	17.4 ± 7.4	>50
10	45.0 ± 5.9	23.7 ± 10.2	>50	29.4 ± 10.9
11	>50	31.8 ± 7.1	>50	>50
12	25.9 ± 4.1	20.3 ± 3.9	22.9 ± 3.1	27.4 ± 5.5
13	27.0 ± 4.0	16.0 ± 6.1	5.8 ± 4.1	22.9 ± 0.9
14/15	1.7 ± 0.1	1.2 ± 0.2	5.6 ± 0.1	1.6 ± 0.2

**IC**_**50**_ **(μM) 72 h treatment**				
1	>10	>10	3.4 ± 2.1	>10
3	>10	>10	9.3 ± 0.3	>10
13	>10	>10	6.4 ± 4.2	>10
14/15	0.9 ± 0.3	1.5 ± 0.4	1.1 ± 0.6	3.4 ± 1.4

**IC**_**50**_ **(μM) 3 × 24 h treatment**				
1	8.9 ± 0.5	>10	2.4 ± 0.3	>10
3	>10	>10	6.0 ± 1.0	>10
13	>10	9.6 ± 0.2	4.6 ± 2.9	>10
14/15	0.7 ± 0.4	1.4 ± 0.4	1.0 ± 0.3	2.7 ± 1.2

The long term cytotoxicity (cytotoxic activity; the direct killing of cancer cells) of 1 and three thiadiazole hybrids (3, 13, and 14/15), that were effective in cytostasis experiments, was also studied on previously used four cell lines. Two setups of experiments were conducted: cells were treated with thiadiazole derivatives for 72 h (protocol 2) or treated 3-times with compounds, without interstitial washing, for 24 h (protocol 3). 1 produced an effect on A431 cells after 72 hours, and on A431 and U87 cells following the 3 × 24 h treatment. 14/15 was the most effective in these experiments too.

Currently, the combination of dabrafenib/trametinib, vemurafenib/cobimetinib, and encorafenib/binimetinib are approved for treating melanoma.^9^ Although there is a rapid early response and high response rate to these combined agents, the progression of disease occurs at a median of eleven months, due to drug resistance; therefore, novel drugs and drug combinations are needed.^26^ Thiadiazole-hybrids might be potential candidates. A2058 cells are found to be more sensitive to 14/15 than to vemurafenib (IC_50_ = 5.93 μM).^27^ If chemotherapy is applied for the treatment of non-melanoma skin cancers, 5-fluorouracil (*e.g.* in the form of its oral prodrug capecitabine or in creams) may be used.^10,11^ 5-Fluorouracil has an IC_50_ value of 47.02 μM for A431 cells.^28^ Thiadiazole derivatives 2, 3, 6, 7, 9, 12, 13 and 14/15 exhibit smaller IC_50_ values therefore are more toxic to A431 cells than this compound. Application of temozolomide in combination with radiotherapy has become a standard of care for glioblastoma multiforme patients; the drug, however, is very expensive and the survival rate of patients is less than two years.^8^ Sorafenib is currently the only effective first-line drug for the treatment of advanced hepatocellular carcinoma patients, however, its efficacy is short owing to the development of resistant cells.^12^ New drugs are therefore required. 3 and thiadiazole-hybrids are much more effective on U87 cell line than the currently used chemotherapeutic agent temozolomide (IC_50_ = 134.97 μM (ref. 29)) and 14/15 is more effective on HepG2 than Sorafenib (IC_50_ = 9.70 μM (ref. 30)). Docetaxel is the mainstay of chemotherapy for prostate cancer with cabazitaxel as second-line drug.^13^1 has also been investigated in clinical trials as a potential chemotherapeutic agent for prostate cancer treatment.^31^ Nine of the synthesized thiadiazole-hybrids proved to be more effective on PC-3 cells than 1 (Table 2). Thiadiazole derivatives studied in this work, especially compounds 14/15, 13, and 3 with the lowest IC_50_ values, may serve as new leads for further study of their antiproliferative properties. We note that the cytostatic effect of these compounds, especially that of 14/15, is outstanding compared to the reference anticancer drug 1.

**Table 2:** *In vitro* cytostatic effect (half maximal inhibitory concentration (IC_50_) values) of compounds 1–14/15 on PC-3 cell line

Label	PC-3	Label	PC-3
**IC**_**50**_ **(μM) 20 h treatment + 72 h incubation**			
1	>50	8	22.7 ± 2.4
2	>50	9	>50
3	3.1 ± 0.3	10	16.9 ± 3.7
4	>50	11	38.0 ± 4.2
5	>50	12	7.7 ± 2.6
6	10.7 ± 0.7	13	4.5 ± 0.5
7	37.6 ± 7.5	14/15	0.4 ± 0.1

**Table 3:** Crystallographic data and details of measurements for compounds 4–6, 8, 10, and 11^a^

Compound	4	6	5	10	11	8
Formula	C_12_H_9_ClFeN_2_S	C_14_H_9_FeIN_2_S	C_14_H_9_ClFeN_2_S	C_22_H_14_FeN_2_S	C_22_H_14_FeN_2_S	C_26_H_18_Fe_2_N_2_S
Fw (g mol^−1^)	304.57	420.04	328.59	394.26	394.26	502.18
*a* (Å)	9.7781(9)	12.0436(10)	7.4925(19)	11.3950(11)	7.4489(12)	9.8936(6)
*b* (Å)	21.088(2)	14.8358(12)	7.5319(16)	19.7263(18)	7.4086(12)	8.8451(5)
*c* (Å)	11.3518(10)	7.5107(6)	11.801(2)	7.6124(8)	32.085(5)	23.0850(13)
*α* (°)	90	90	102.546(17)	90	90	90
*β* (°)	99.460(4)	91.413(4)	103.317(17)	92.122(6)	93.571(8)	99.728(5)
*γ* (°)	90	90	92.575(18)	90	90	90
*V* (Å^3^)	2308.9(4)	1341.58(19)	Block, orange	1710.0(3)	1767.2(5)	1991.1(2)
*Z*	8	4	2	4	4	4
Crystal size (mm)	0.08 × 0.08 × 0.07	0.04 × 0.04 × 0.01	0.14 × 0.13 × 0.10	0.05 × 0.04 × 0.04	0.05 × 0.05 × 0.01	0.27 × 0.19 × 0.15
Crystal habit	Block, red	Plate, orange	Block, orange	Block, red	Plate, red	Block, red
Crystal system	Monoclinic	Monoclinic	Triclinic	Monoclinic	Monoclinic	Monoclinic
Space group	*P*2_1_/*c*	*P*2_1_/*c*	*P*1̄	*P*2_1_/*c*	*P*2_1_/*c*	*P*2_1_/*c*
*d*_calc_ (mg m^−3^)	1.752	2.080	1.734	1.531	1.482	1.675
*μ* (mm^−1^)	1.69	1.69	1.56	1.01	0.98	1.58
*T* (K)	100(2)	100(2)	100(2)	100(2)	100(2)	100(2)
2*θ* range (°)	2.3–33.2	3.4–32.4	2.8–33.3	2.9–33.2	2.5–33.2	2.5–33.2
*F*(000)	1232	808	332	808	808	1024
*T*_min_, *T*_max_	0.556, 0.741	0.522, 0.747	0.622, 0.747	0.632, 0.747	0.372, 0.747	0.587, 0.747
*R*_int_	0.056	0.117	0.048	0.102	0.25	0.106
No. of measured, independent and observed [*I* > 2 s(*I*)] reflections	186019, 8831, 7915	31160, 2340, 1838	39429, 2191, 2128	48371, 3001, 2421	3092, 3092, 2241	196783, 7626, 6753
Independent reflections	8831	2340	2191	3001	3092	7626
No. of parameters, restraints	307, 0	173, 0	172, 0	235, 0	235, 0	299, 0
*Δ*_max_, *Δ*_min_ (e Å^−3^)	0.69, −0.35	0.61, −0.60	0.34, −0.18	0.28, −0.33	0.60, −0.93	0.60, −0.60
*R*1, w*R*2 (all data)	*R*1 = 0.0314	*R*1 = 0.0503	*R*1 = 0.0177	*R*1 = 0.0499	*R*1 = 0.1247	*R*1 = 0.0343
	w*R*2 = 0.0679	w*R*2 = 0.0669	w*R*2 = 0.0445	w*R*2 = 0.0750	w*R*2 = 0.1837	w*R*2 = 0.0709
*R*1, w*R*2 (>2*σ*)	*R*1 = 0.02*5*5	*R*1 = 0.0297	*R*1 = 0.0168	*R*1 = 0.0317	*R*1 = 0.0862	*R*1 = 0.0287
	w*R*2 = 0.0630	w*R*2 = 0.0574	w*R*2 = 0.0437	w*R*2 = 0.0649	w*R*2 = 0.1693	w*R*2 = 0.0681

a Mo Kα (*λ* = 0.71073 Å). *R*_1_ = *Σ*/|*F*_o_| − |*F*_c_|/|*Σ*|*F*_d_; w*R*2 = [*Σ*_w_(*F*_o_^2^ − *F*_2_^2^)^2^/*Σ*_w_(*F*_o_^2^)^2^]^1/2^.

Finally, it is of crucial importance to provide evidence for that the intact hybrid molecules rather than any of their decomposition-derived fragments are the species which induce evolution of the antiproliferative effect in the course of the biological assays employing long-term treatment of the cells. According to our first observations the synthesized compounds in solid state were stable at ambient conditions; decomposition or colour change were not observed over a couple of months. Moreover, these compounds were not sensitive to air, moisture or light when handled at ambient conditions, and can generally be stored in closed vial at room temperature in the dark without decomposition as proved by IR- and MS measurements. The long-term stability of the compounds in solution was also checked by registering their ^1^H-NMR spectra in DMSO-d_6_ after 72 h following the preparation of the liquid samples stored under air at room temperature. Supporting our abovementioned observations regarding the stability of the novel hybrids, their spectra did not show any detectable change in their structures, although the solvent was contaminated with a substantial amount of HDO.

## Experimental

### Materials and spectroscopic methods

Chemicals, namely precursors (1, 2, ethynylferrocene, ferroceneboronic acid, phenylacetylene; purity ≥ 97%), catalysts and ligands (palladium(ii) acetate, [Pd(OAc)_2_], bis(triphenylphosphine) palladium dichloride, [PdCl_2_(PPh_3_)_2_], bis(tri-*tert*-butylphosphine)palladium(0), [Pd[P(*t*-Bu)_3_]_2_], and triphenylphosphine, PPh_3_; purity ≥ 98%), bases (diisopropylamine, triethylamine, potassium carbonate, K_2_CO_3_, and tripotassium phosphate, K_3_PO_4_; purity ≥ 98%), and anhydrous solvents (1,4-dioxane, toluene, *N*,*N*-dimethylformamide; purity ≥ 99.8%) were purchased from commercial sources (Sigma-Aldrich, Fluorochem, VWR) and used, except solvents, without further purification. Solvents were dried according to published methods^32^ and distilled before use. Compounds 3, 7 and 9 were synthesized as described recently.^25^ Synthetic reactions were monitored by thin-layer chromatography (TLC) using Merck Silica gel 60 F254 TLC plates, and plates were visualised under a Camag model UV lamp 4 dual wavelength 254/366 nm. Column chromatography was performed using Merck silica gel 60 (0.063–0.200 mm) using a column (diameter 2.5 cm) with sintered glass disc.

The ^1^H- and ^13^C-NMR spectra of the synthesized compounds were recorded on a Bruker DRX-500 MHz spectrometer at 500 MHz and 125 MHz, respectively, at room temperature using the deuterium signal of the solvent as the lock and tetramethylsilane (TMS) as the internal standard. The assignment of all ^1^H- and ^13^C-NMR data necessary for exact structural elucidation of the compounds was based on the cross-peak correlations discernible in 2D-HSQC and HMBC spectra obtained by standard Bruker pulse programs. Mass spectroscopic measurements were done using an Esquire 3000 + (Bruker) ion trap mass spectrometer and electrospray ionization (ESI). Exact mass measurements for samples 12, 13, and 14/15 were taken on a high-resolution Waters Q-Tof Premier mass spectrometer equipped with an ESI ion source (3000 V capillary voltage, 350 °C desolvation temperature, 650 L h^−1^ nitrogen as desolvation gas). The samples were dissolved in methanol (10 μg mL^−1^) and 5 μL were injected in a continuous flow of methanol (400 μL min^−1^, contain 0.1% formic acid). Each compound was analysed twice, the first spectrum was recorded without consideration of temperature variations. The second measurement was processed after the correction of temperature variations with a reference compound.

### Synthetic procedures and characterisation of the thiadiazole hybrid products

#### 3-Chloro-5-ferrocenyl-1,2,4-thiadiazole (4)

A mixture of 3,5-dichloro-1,2,4-thiadiazole, 2 (1 mmol, 0.16 g), ferroceneboronic acid (1.5 mmol, 0.34 g), Pd(OAc)_2_ (5 mol%, 0.01 g), PPh_3_ (15 mol%, 0.04 g) and K_2_CO_3_ (3 mmol, 0.41 g) in freshly distilled dioxane (5 ml) was stirred vigorously and heated to reflux for 14 h under nitrogen atmosphere. The reaction was monitored by TLC using hexane/ethyl acetate (95 : 5) as eluent. After completion of the reaction, the resulting mixture was cooled to room temperature, and the solvent was evaporated *in vacuo*. The crude material was dissolved with chloroform, washed with water, dried over MgSO_4_ and concentrated under reduced pressure. The residue was purified by column chromatography on silica gel using hexane/ethyl acetate (95 : 5) as eluent to afford the pure red crystalline product 4 (yield: 0.24 g, 78%). Mp: 124–126 °C.
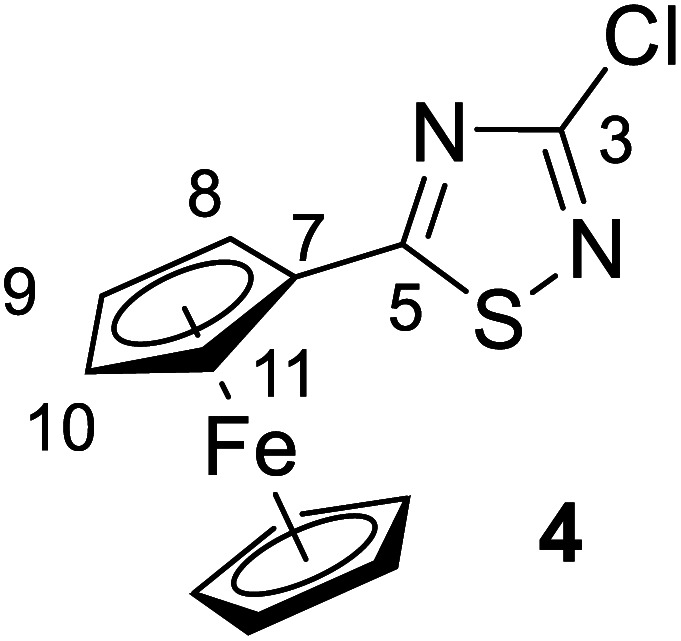


Spectroscopic data: MS: 305.0 *m*/*z* [M + 1^+^]. IR (neat, ATR): 3090 (w), 2923 (w), 2853 (vw), 1516 (s), 1430 (s), 1385 (w), 1350 (vw), 1235 (vs), 1201 (m), 1104 (m), 1064 (w), 1028 (w), 999 (m), 927 (vw), 822 (m), 752 (w), 716 (m), 693 (m), 641 (w), 507 (m), 481 (m) cm^−1^. ^1^H NMR (CDCl_3_): 4.89 (br ∼ s, 2H, H-8,11); 4.57 (br ∼ s, 2H, H-9,10); 4.14 (s, 5H, η^5^-C_5_H̲_5_) ppm; ^13^C NMR (CDCl_3_): 192.8 (C-5); 157.0 (C-3); 73,4 (C-7); 72.0 (C-9,10), 70.9 (η^5^-CH̲_5_H_5_); 68.8 (C-8,11) ppm.

#### 3-Halogeno-5-ferrocenethynyl-1,2,4-thiadiazole (5–6)

A mixture of 3,5-dihalogeno-1,2,4-thiadiazole, 2 (1 mmol, 0.16 g) or 3 (1 mmol, 0.34 g), ethynylferrocene (1.1 mmol, 0.23 g), Pd(PPh_3_)_2_Cl_2_ (3 mol%, 0.02 g), CuI (3 mol%, 0.006 g), and diisopropylamine (1.1 mmol, 0.11 g) in freshly distilled toluene (5 mL) was stirred at 50 °C for 6 h under a nitrogen atmosphere. The reaction was monitored by TLC using hexane/ethyl acetate (97 : 3) as the eluent. After completion of the reaction, the resulting mixture was cooled to room temperature, and the solvent was evaporated *in vacuo*. The crude material was dissolved with chloroform, washed with water, dried over MgSO_4_ and concentrated under reduced pressure. The residue was purified by column chromatography on silica gel using hexane/ethyl acetate (97 : 3) as eluent to afford the pure orange-red crystalline products 5 (yield: 0.27 g, 82%, mp: 167–169 °C) or 6 (yield: 0.37 g, 87%, mp: 161–163 °C).
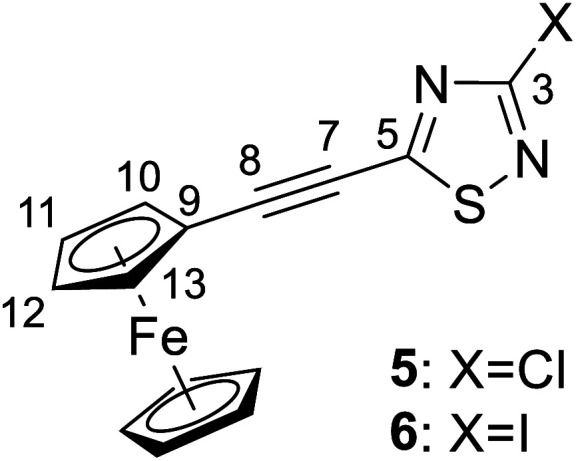


Spectroscopic data of 5: MS: 328.9 *m*/*z* [M + 1^+^]. IR (neat, ATR): 3095 (vw), 2955 (vw), 2922 (w), 2854 (vw), 2200 (vs), 1501 (m), 1420 (s), 1381 (m), 1355 (w), 1259 (w), 1228 (vs), 1116 (s), 1028 (m), 1000 (w), 943 (m), 818 (s), 731 (w), 682 (w), 623 (vw), 541 (w), 495 (s), 456 (m) cm^−1^. ^1^H-NMR (CDCl_3_): 4.61 (t, *J* = 1.8 Hz, 2H, H-10,13); 4.40 (t, *J* = 1.8 Hz, 2H, H-11,12); 4.26 (s, 5H, η^5^-C_5̲__5_); ^13^C-NMR (125 MHz): 171.7 (C-5); 157.5 (C-3); 107.6 (C-8); 75.7 (C-7); 72.6 (C-11,12); 70.8 (C-10,13); 70.5 (η^5^-__5_H_5_); 60.5 (C-9) ppm.

Spectroscopic data of 6: MS: 419.9 *m*/*z* [M^+^]. IR (neat, ATR): 3097 (vw), 2959 (vw), 2923 (vw), 2857 (vw), 2199 (vs), 1495 (w), 1407 (m), 1373 (w), 1340 (w), 1252 (w), 1187 (s), 1115 (m), 1029 (w), 1002 (vw), 923 (w), 888 (w), 822 (m), 789 (w), 727 (vw), 666 (vw), 625 (vw), 537 (vw), 488 (m) cm^−1^. ^1^H-NMR (CDCl_3_): 4.60 (t, *J* = 1.8 Hz, 2H, H-10,13); 4.39 (t, *J* = 1.8 Hz, 2H, H-11,12); 4.25 (s, 5H, η^5^-C_5_H̲_5_); ^13^C-NMR (CDCl_3_): 171.2 (C-5); 117.6 (C-3); 107.9 (C-8); 75.0 (C-7); 72.5 (C-11,12); 70.8 (C-10,13); 70.5 (η^5^-C̲_5_H_5_); 60.5 (C-9) ppm.

#### 3,5-Bis(ferrocenethynyl)-1,2,4-thiadiazole (8)

Procedure A: a mixture of 5 (1 mmol, 0.33 g) or 6 (1 mmol, 0.42 g), ethynylferrocene (1.5 mmol, 0.32 g), Pd(PPh_3_)_2_Cl_2_ (3 mol%, 0.02 g), CuI (3 mol%, 0.006 g), and diisopropylamine (1.5 mmol, 0.15 g) in freshly distilled toluene (5 mL) was stirred at 70 °C for 8 h under a nitrogen atmosphere. The reaction was monitored by TLC using hexane/ethyl acetate (95 : 5) as the eluent. After completion of the reaction, the resulting mixture was cooled to room temperature, and the solvent was evaporated *in vacuo*. The crude material was dissolved with chloroform, washed with water, dried over MgSO_4_ and concentrated under reduced pressure. The residue was purified by column chromatography on silica gel using hexane/ethyl acetate (95 : 5) as eluent to afford the pure dark red crystalline product 8 (yield: 0.39 g, 78%). Mp: 218–220 °C.

Procedure B: a mixture of 2 (1 mmol, 0.16 g) or 3 (1 mmol, 0.34 g), ethynylferrocene (3 mmol, 0.63 g), Pd(PPh_3_)_2_Cl_2_ (3 mol%, 0.02 g), CuI (3 mol%, 0.006 g), and diisopropylamine (3 mmol, 0.3 g) in freshly distilled toluene (5 mL) was stirred at 70 °C for 12 h under a nitrogen atmosphere. The reaction was monitored by TLC using hexane/ethyl acetate (95 : 5) as the eluent. After completion of the reaction, the resulting mixture was cooled to room temperature, and the solvent was evaporated *in vacuo*. The crude material was dissolved with chloroform, washed with water, dried over MgSO_4_ and concentrated under reduced pressure. The residue was purified by column chromatography on silica gel using hexane/ethyl acetate (95 : 5) as eluent to afford the pure dark red crystalline product 8 (yield: 0.33 g, 66%). Mp: 218–220 °C.
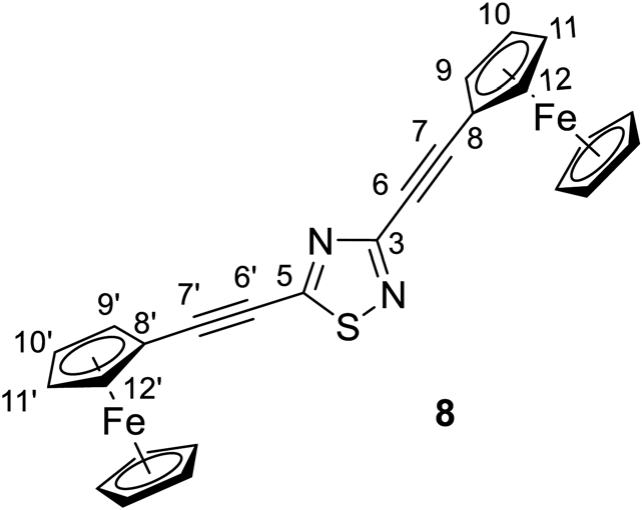


Spectroscopic data: MS: 503.0 *m*/*z* [M + 1^+^]. IR (neat, ATR): 3102 (vw), 2956 (w, sh), 2920 (s), 2851 (m), 2202 (vs), 1480 (m), 1413 (s), 1367 (w), 1283 (w), 1255 (m), 1212 (s), 1109 (s), 1028 (s), 892 (vw), 821 (s), 718 (w), 544 (w), 488 (s), 455 (m), 412 (vw) cm^−1^. ^1^H-NMR (CDCl_3_): 4.61 and 4.60 (two partly overlapping t's, *J* ∼ 2 Hz for each, 4H, H-9,9′,12,12′); 4.40 (two overlapping t's, *J* = 1.8 Hz, 4H, H-10,10′,11,11′); 4.26 and 4.24 (2 × s, 2 × 5H, 2 × η^5^-C_5_H̲_5_); ^13^C-NMR (CDCl_3_): 169.1 (C-5); 157.8 (C-3); 105.6 (C-7′); 90.1 (C-7); 79.0 (C-6); 75.7 (C-6′); 72.4 and 72.3 (C-9,9′,10,10′, 11,11′,12,12′); 70.5 and 70.3 (2 × η^5^-C̲_5_H_5_); 62.2 and 61.1 (C-8,8′) ppm.

#### 3-(Ferrocenethynyl)-5-(phenylethynyl)-1,2,4-thiadiazole (10)

A mixture of 7 (1 mmol, 0.31 g), ethynylferrocene (1.5 mmol, 0.32 g), Pd(PPh_3_)_2_Cl_2_ (3 mol%, 0.02 g), CuI (3 mol%, 0.006 g), and diisopropylamine (1.5 mmol, 0.15 g) in freshly distilled toluene (5 mL) was stirred at 70 °C for 8 h under a nitrogen atmosphere. The reaction was monitored by TLC using hexane/ethyl acetate (95 : 5) as the eluent. After completion of the reaction, the resulting mixture was cooled to room temperature, and the solvent was evaporated *in vacuo*. The crude material was dissolved with chloroform, washed with water, dried over MgSO_4_, and concentrated under reduced pressure. The residue was purified by column chromatography on silica gel using hexane/ethyl acetate (95 : 5) as eluent to afford the pure red crystalline product 10 (yield: 0.30 g, 75%). Mp: 170–172 °C.
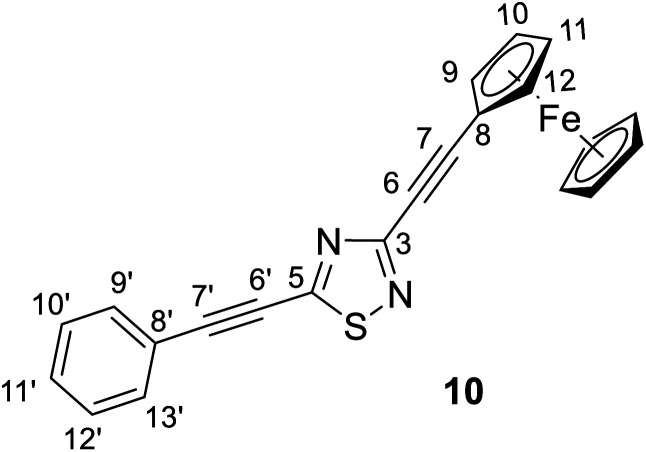


Spectroscopic data: MS: 595.1 *m*/*z* [M + 1^+^]. IR (neat, ATR): 3094 (vw), 3055 (vw), 2959 (w), 2924 (w), 2855 (vw), 2219 (vs), 1490 (m), 1450 (m), 1397 (m), 1367 (m), 1287 (m), 1217 (s), 1103 (m), 1041 (w), 999 (w), 972 (vw), 914 (vw), 824 (m), 756 (m), 718 (w), 687 (m), 581 (w), 535 (w), 495 (m) cm^−1^. ^1^H-NMR (CDCl_3_): 7.59 (dd, *J* = 7.8 Hz and 1.8 Hz, 2H, H-13′,15′); 7,45 (tt, *J* = 7.8 Hz and 1.8 Hz, 1H, H-11′); 7.40 (∼t, *J* ∼ 8 Hz, 2H, H-10′, 12′); 4.62 (t, *J* = 1.9 Hz, 2H, H-9,12); 4.30 (t, *J* = 1.9 Hz, 2H, H-10,11); 4.24 (s, 5H, η^5^-C_5_H̲_5_); ^13^C-NMR (CDCl_3_): 168.7 (C-5); 157.9 (C-3); 132.3 (C-9′,13′); 130.7 (C-11′); 128.8 (C-10′, 12′); 120.4 (C-8′); 103.2 (C-7′); 90.6 (C-7); 78.9 (C-6′); 78.5 (C-6); 72.4 (C-9,12); 70.3 (η^5^-C̲_5_H_5_); 69.8 (C-10,11); 62.0 (C-8) ppm.

#### 3-(Phenylethynyl)-5-(ferrocenethynyl)-1,2,4-thiadiazole (11)

A mixture of 6 (1 mmol, 0.42 g), phenylacetylene (1.5 mmol, 0.15 g), Pd(PPh_3_)_2_Cl_2_ (3 mol%, 0.02 g), CuI (3 mol%, 0.006 g), and diisopropylamine (1.5 mmol, 0.15 g) in freshly distilled toluene (5 mL) was stirred at 70 °C for 8 h under a nitrogen atmosphere. The reaction was monitored by TLC using hexane/ethyl acetate (95 : 5) as the eluent. After completion of the reaction, the resulting mixture was cooled to room temperature, and the solvent was evaporated *in vacuo*. The crude material was dissolved with chloroform, washed with water, dried over MgSO_4_ and concentrated under reduced pressure. The residue was purified by column chromatography on silica gel using hexane/ethyl acetate (95 : 5) as eluent to afford the pure red crystalline product 11 (yield: 0.31 g, 79%). Mp: 183–185 °C.
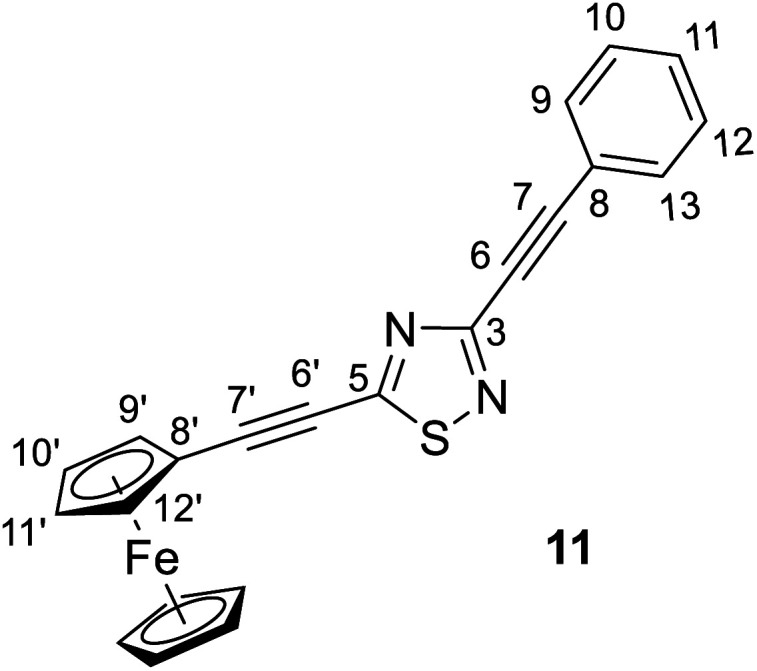


Spectroscopic data: MS: 595.1 *m*/*z* [M + 1^+^]. IR (neat, ATR): 3092 (vw), 3056 (vw), 2923 (vw), 2850 (vvw), 2201 (vs), 1494 (m), 1425 (s), 1292 (m), 1255 (m), 1198 (m), 1117 (m), 1063 (vw), 1030 (m), 1001 (w), 914 (w), 827 (m), 757 (m), 722 (w), 689 (m), 579 (vw), 533 (w), 484 (m) cm^−1^. ^1^H-NMR (CDCl_3_): 7.62 (dd, *J* = 7.8 Hz and 1.8 Hz, 2H, H-9,13); 7,40 (tt, *J* = 7.8 Hz and 1.8 Hz, 1H, H-11); 7.36 (∼t, *J* ∼ 8 Hz, 2H, H-10, 12); 4.61 (t, *J* = 1.8 Hz, 2H, H-9′,12′); 4.30 (t, *J* = 1.8 Hz, 2H, H-10′,11′); 4.26 (s, 5H, η^5^-C_5_H̲_5_); ^13^C-NMR (CDCl_3_): 169.4 (C-5); 157.4 (C-3); 132.3 (C-9,13); 129.8 (C-11); 128.5 (C-10, 12); 121.2 (C-8); 105.9 (C-7′); 89.2 (C-7); 82.0 (C-6); 75.7 (C-6′); 72.4 (C-9′,12′); 70.6 (C-10′,11′); 70.5 (η^5^-C̲_5_H_5_); 61.0 (C-8) ppm.

#### *N*-(3-((3-Halogeno-1,2,4-thiadiazol-5-yl)ethynyl)phenyl)-6,7-bis(2-methoxyethoxy)quinazolin-4-amine (12 and 13)

A mixture of 2 (1 mmol, 0.16 g) or 3 (1 mmol, 0.34 g), erlotinib (1.1 mmol, 0.43 g), Pd[P(*t*-Bu)_3_]_2_ (10 mol%, 0.05 g), CuI (10 mol%, 0.02 g) and K_3_PO_4_ (1.1 mmol, 0.23 g) in freshly distilled DMF (3 mL) was stirred vigorously at 80 °C for 12 h under a nitrogen atmosphere. The reaction was monitored by TLC using hexane/ethyl acetate (50 : 50) as the eluent. After completion of the reaction, the resulting mixture was quenched by water (30 mL), and extracted with chloroform (3 × 20 mL). The combined organic layers were washed with water, dried over anhydrous MgSO_4_, and concentrated under reduced pressure. The residue was purified by column chromatography on silica gel using hexane/ethyl acetate (50 : 50) as eluent to afford the pure light yellow products 12 (yield: 0.36 g, 71%, mp: 125–127 °C) or 13 (yield: 0.45 g, 75%, mp: 118–120 °C).
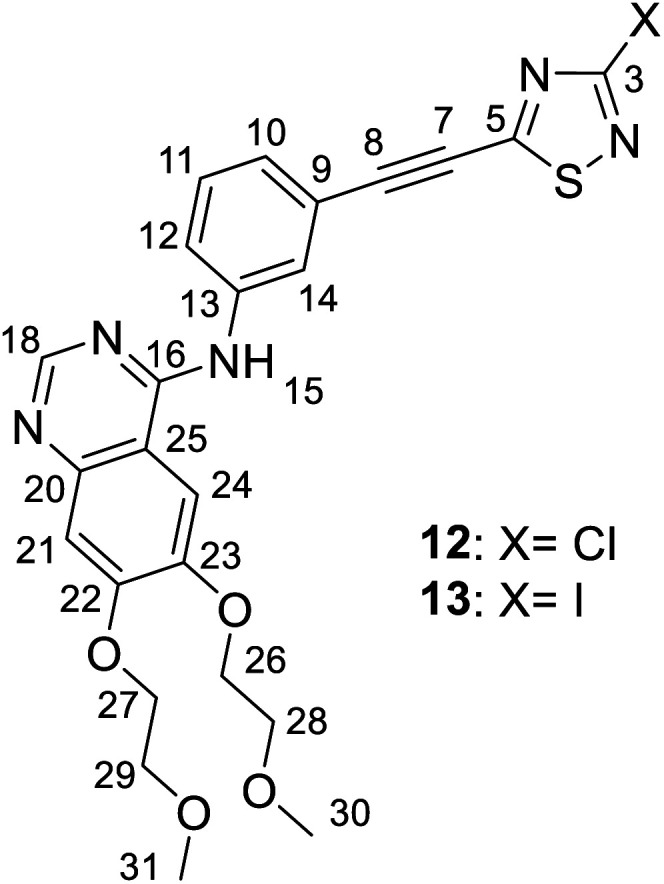


Spectroscopic data of 12: MS: 511.7 *m*/*z* [M + 1^+^]; HR-MS: 512.1155 *m*/*z* (most abundant [M + 1^+^] isotopic peak; theoretical value: 512.1159). IR (neat, ATR): 3485 (w), 3321 (w), 3115 (w), 3073 (w), 2979 (vw), 2923 (w), 2882 (w), 2817 (w), 2205 (m), 1625 (m), 1573 (m), 1509 (m), 1460 (m), 1428 (s), 1391 (w), 1366 (w), 1282 (w), 1222 (s), 1118 (m), 1100 (m), 1065 (w), 1022 (m), 959 (w), 929 (m), 894 (w), 861 (m), 786 (m), 681 (m), 616 (w), 576 (m), 550 (w), 465 (w) cm^−1^. ^1^H-NMR (DMSO-d_6_): 9.51 (s, 1H, H-15); 8.49 (s, 1H, H-18); 8.25 (br s, 1H, H-14); 7.95 (br d, *J* ∼ 8 Hz, 1H, H-12); 7.65 (s, 1H, H-24); 7.50 (t, *J* ∼ 8.0 Hz, 1H, H-11); 7.44 (br d, *J* = 8 Hz, 1H, H-10); 7.20 (s, 1H, H-21); 4.26 (m, 4H, H-26,27); 3.75 and 3.71 (two partly overlapping t's, *J* ∼ 6 Hz for each, 2 × 2H, H-28,29); 3.33 and 3.31 (2 × s, 2 × 3H, H-30,31). ^13^C-NMR (DMSO-d_6_): 171.0 (C-5); 157.9 (C-3); 156.5 (C-16); 154.3 (C-22); 153.2 (C-18); 148.8 (C-23); 147.3 (C-20); 140.6 (C-13); 129.9 (C-11); 127.3 (C-10); 125.4 (C-14); 125.1 (C-12); 119.8 (C-9); 109.4 (C-25); 108.6 (C-21); 104.3 (C-8); 103.8 (C-24); 78.3 (C-7); 70.6 and 70.5 (C-26,27); 68.9 and 68.6 (C-28,29); 58.9 (two coalesced lines, C-30,31) ppm.

Spectroscopic data of 13: MS: 603.8 *m*/*z* [M + 1^+^]; HR-MS: 604.0517 *m*/*z* (most abundant [M + 1^+^] isotopic peak; theoretical value: 604.0516). IR (neat, ATR): 3479 (vw), 3318 (vw), 3123 (vw), 3074 (vw), 2919 (m), 2852 (w), 2817 (vw), 2202 (m), 1625 (m), 1575 (m), 1511 (m), 1456 (m), 1426 (s), 1342 (w), 1278 (vw), 1247 (w), 1221 (w), 1181 (m), 1124 (w), 1093 (w), 1065 (w), 1026 (w), 947 (w), 928 (w), 888 (w), 858 (m), 784 (m), 675 (w), 574 (w), 542 (w), 457 (w) cm^−1^. ^1^H-NMR (DMSO-d_6_): 9.51 (s, 1H, H-15); 8.50 (s, 1H, H-18); 8.24 (br s, 1H, H-14); 7.97 (br d *J* ∼ 8 Hz, 1H, H-12); 7.64 (s, 1H, H-24); 7.50 (t, *J* ∼ 8.0 Hz, 1H, H-11); 7.42 (br d, *J* = 8 Hz, 1H, H-10); 7.20 (s, 1H, H-21); 4.26 (m, 4H, H-26,27); 3.75 and 3.71 (two partly overlapping t's, *J* ∼ 6 Hz for each, 2 × 2H, H-28,29); 3.33 and 3.31 (2 × s, 2 × 3H, H-30,31). ^13^C-NMR (DMSO-d_6_): 170.6 (C-5); 156.5 (C-16); 154.3 (C-22); 153.2 (C-18); 148.8 (C-23); 147.3 (C-20); 140.6 (C-13); 132.4 (C-3); 129.9 (C-11); 127.3 (C-10); 125.4 (C-14); 125.1 (C-12); 119.8 (C-9); 109.4 (C-25); 108.6 (C-21); 104.6 (C-8); 103.8 (C-24); 78.0 (C-7); 70.60 and 70.54 (C-26,27); 68.9 and 68.6 (C-28,29); 58.87 and 58.82 (C-30,31) ppm.

#### Isomer mixture of *N*,*N*′-(((1,2,4-thiadiazole-3,5-diyl)bis(ethyne-2,1-diyl))bis(3,1-phenylene))bis(6,7-bis(2-methoxyethoxy)quinazolin-4-amine) and *N*,*N*′-(((1,3,4-thiadiazole-2,5-diyl)bis(ethyne-2,1-diyl))bis(3,1-phenylene))bis(6,7-bis(2-methoxyethoxy)quinazolin-4-amine) (14/15)

Procedure A: a mixture of 12 (1 mmol, 0.5 g) or 13 (1 mmol, 0.6 g), erlotinib (1.5 mmol, 0.6 g), Pd[P(*t*-Bu)_3_]_2_ (10 mol%, 0.05 g), CuI (10 mol%, 0.02 g) and K_3_PO_4_ (1.5 mmol, 0.32 g) in freshly distilled DMF (3 mL) was stirred vigorously at 95 °C for 15 h under a nitrogen atmosphere. The reaction was monitored by TLC using hexane/ethyl acetate (30 : 70) as the eluent. After completion of the reaction, the resulting mixture was quenched by water (30 mL), and extracted with chloroform (3 × 20 mL). The combined organic layers were washed with water, dried over anhydrous MgSO_4_, and concentrated under reduced pressure. The residue was purified by column chromatography on silica gel using hexane/ethyl acetate (30 : 70) as eluent to afford the pure white product 14/15 (yield: 0.61 g, 70%, mp: 132–134 °C).

Procedure B: a mixture of 2 (1 mmol, 0.16 g) or 3 (1 mmol, 0.34 g), erlotinib (3 mmol, 1.2 g), Pd[P(*t*-Bu)_3_]_2_ (10 mol%, 0.05 g), CuI (10 mol%, 0.02 g) and K_3_PO_4_ (3 mmol, 0.64 g) in freshly distilled DMF (3 mL) was stirred vigorously at 95 °C for 20 h under a nitrogen atmosphere. The reaction was monitored by TLC using hexane/ethyl acetate (30 : 70) as the eluent. After completion of the reaction, the resulting mixture was quenched by water (30 mL), and extracted with chloroform (3 × 20 mL). The combined organic layers were washed with water, dried over anhydrous MgSO_4_, and concentrated under reduced pressure. The residue was purified by column chromatography on silica gel using hexane/ethyl acetate (30 : 70) as eluent to afford the pure white product 14/15 (yield: 0.53 g, 61%). Mp: 132–134 °C.
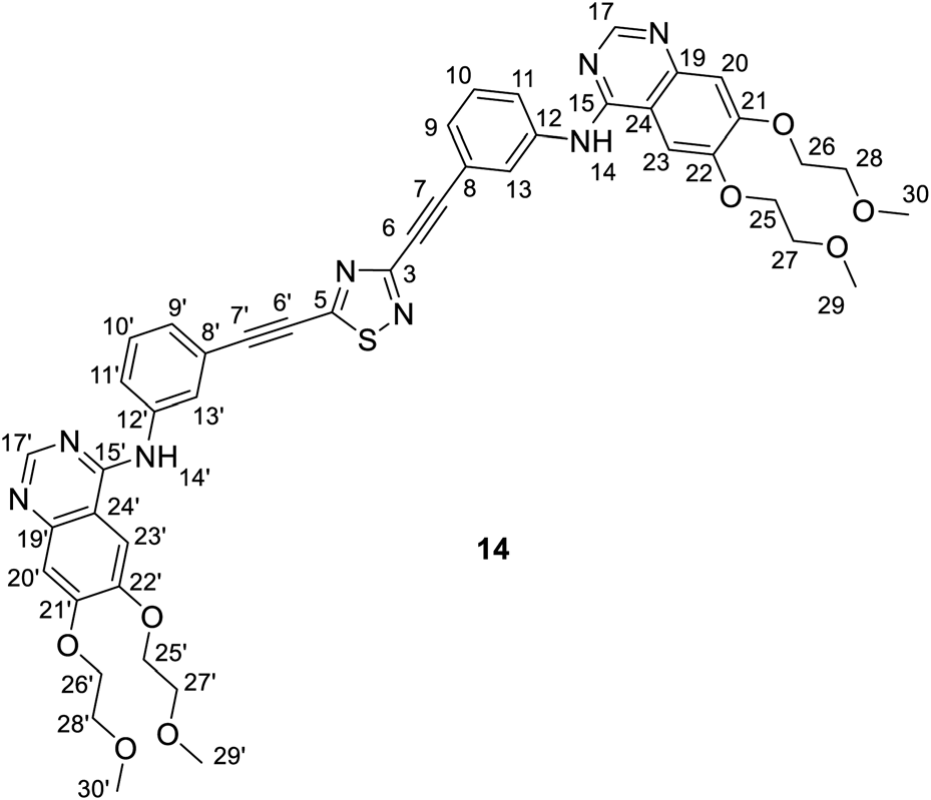

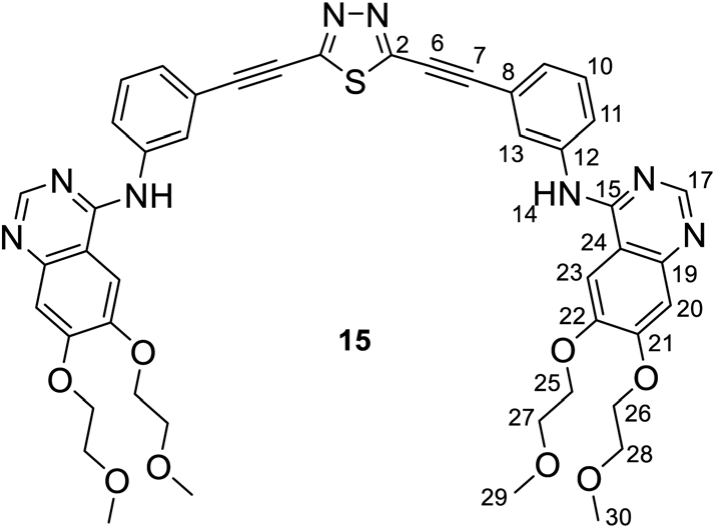


Spectroscopic data of a *ca.* 3/2 mixture of 14/15: MS: 869.4 [M + 1^+^], 435.4 [(M + 2)^2+^] *m*/*z*; HR-MS: 869.3080 *m*/*z* (most abundant [M + 1^+^] isotopic peak; theoretical value: 869.3081). IR (neat, ATR): 3483 (vw), 3447 (vw), 3318 (vw), 3272 (vw), 3063 (vw), 2976 (vw), 2926 (w), 2883 (w), 2820 (w), 2227 (s), 1625 (m), 1576 (m), 1507 (m), 1428 (s), 1391 (m), 1281 (w), 1242 (m), 1212 (m), 1124 (m), 1068 (w), 1028 (m), 933 (w), 894 (vw), 858 (m), 784 (m), 716 (vw), 684 (w), 658 (w), 579 (w), 549 (w), 465 (w) cm^−1^.

NMR data of 14: ^1^H-NMR (DMSO-d_6_): 9.56 (s, 1H, H-14′); 9.53 (s, 1H, H-14); 8.51 (s, 1H, H-17′); 8.50 (s, 1H, H-17); 8.27 (br s, 1H, H-13′); 8.25 (br s, 1H, H-13); 7.99 (br d, *J* ∼ 8 Hz, 1H, H-11′); 7.96 (br d, *J* ∼ 8 Hz, 1H, H-11); 7.85 (two coalesced s', 2H, H-23,23′); 7.52 (t, *J* ∼ 8.0 Hz, 1H, H-10′); 7.49 (t, *J* ∼ 8.0 Hz, 1H, H-10); 7.44 (br d, *J* ∼ 8 Hz, 1H, H-9′); 7.39 (br d, *J* ∼ 8 Hz, 1H, H-9); 7.20 (two coalesced s', 2H, H-20,20′); 4.26 (m, 8H, H-25,25′,26,26′); 3.75 and 3.71 (m, 8H, H-27,27′,28′); 3.33 and 3.31 (2 × s, 2 × 6H, H-29,29′,30,30′). ^13^C-NMR (DMSO-d_6_): 169.1 (C-5); 156.7 (C-3); 156.6 (two coalesced lines, C-15,15′); 154.31 and 154.27 (C-21,21′); 153.2 (two coalesced lines, C-17,17′); 148.76 (two coalesced lines, C-22,22′); 147.59 and 147.55 (C-19,19′); 140.44 (two coalesced lines, C-12,12′); 129.9 (C-10′); 129.8 (C-10); 127.16 and 127.23 (C-9,9′); 126.4 (two coalesced lines, C-13,13′); 125.0 (C-11′); 124.3 (C-11); 121.0 (C-8); 119.6 (C-8′); 109.4 (two coalesced lines, C-24,24′); 108.7 (two coalesced lines, C-20,20′); 103.8 (two coalesced lines, C-23,23′); 82.4 (C-7,7′); 73.8 (C-6,6′); 70.60 and 70.57 (C-25,26′,25′,26′); 68.96 and 68.62 (C-27,28,27′,28′); 58.92 and 58.85 (C-29,30,29′,30′) ppm.

NMR data of 15: ^1^H-NMR (DMSO-d_6_): 9.50 (s, 1H, H-14); 8.48 (s, 1H, H-17); 8.12 (br s, 1H, H-13); 7.89 (br d, *J* ∼ 8 Hz, 1H, H-11); 7.83 (s, 1H, H-23); 7.43 (t, *J* ∼ 8.0 Hz, 1H, H-10); 7.31 (br d, *J* ∼ 8 Hz, 1H, H-9); 7.19 (s, 1H, H-20); 4.26 (m, 4H, H-25,26); 3.75 and 3.71 (m, 4H, H-27,28); 3.33 and 3.31 (2 × s, 2 × 3H, H-29,30). ^13^C-NMR (DMSO-d_6_): 156.7 (C-2); 156.6; (C-15); 154.19 (C-21); 153.2 (C-17); 148.68 (C-22); 147.5 (C-19); 140.4 (C-12); 129.7 (C-10); 127.6 (C-9); 126.3 (C-13); 124.1 (C-11); 121.3 (C-8); 109.4 (C-24); 108.5 (C-20); 103.7 (C-23); 82.4 (C-7); 73.8 (C-6); 70.60 and 70.57 (C-25,26); 68.96 and 68.62 (C-27,28); 58.92 and 58.85 (C-29,30) ppm.

For compounds 14 and 15 the signals from nuclei H-20,20′, H-23,23′, H-25,25′-H-30,30′ and C-19,19′-C-30,30′ are exactly coalesced or almost completely overlapped.

### Crystallography

Single crystals of 4–6, 8, 10, 11 suitable for single crystal X-ray diffractometry were grown from chloroform (4, 6, 5, 11) or chloroform/ethanol 50/50 (v/v) mixture (8, 10) by slow evaporation of the solvent at room temperature. Crystals were removed from a vial and immediately covered with a layer of silicone oil. A single crystal was selected, mounted on a glass rod on a copper pin, and placed in the cold N_2_ stream provided by an Oxford Cryosystems cryostream. XRD data collection was performed for 4–6, 8, 10, 11 on a Bruker APEX II diffractometer with the use of an IμS microsource (Incoatec microfocus) sealed tube of Mo Kα radiation (*λ* = 0.71073 Å) and a CCD area detector. Empirical absorption corrections were applied using SADABS or TWINABS.^33,34^ The structures were solved with the use of the intrinsic phasing option in SHELXT and refined by the full-matrix least-squares procedures in SHELXL.^35–37^ The space group assignments and structural solutions were evaluated using PLATON.^38,39^ Non-hydrogen atoms were refined anisotropically. Hydrogen atoms were located in calculated positions corresponding to standard bond lengths and angles. Substitutional disorder in compound 8 for atoms S1 and N2 in the thiadiazolidine ring was handled by modeling the occupancies of the individual orientations using free variables to refine the respective occupancy of the affected fragments (PART).^40^ Compound 6 was refined using the TWIN option in SHELXL as a 2-component twin (−1 0 0 0–1 0 0 0 1). Compound 4 was refined using the TWIN option in SHELXL as a 2-component twin (−1 0 0 0–1 0 0 0 1). Electrostatic non-covalent intermolecular interactions,^41–44^ van der Waals contacts (C–H⋯X),^45–47^ and halide interactions (X⋯N, X⋯S, S⋯S, X⋯X)^48–51^ were determined by the programs Mercury^52^ and Diamond^53^ using the centroids and planes and other features of these programs. All values for published compounds were based on a Cambridge Structural Database^54^ search and all values for presented and published compounds fall within expected ranges. All crystal structures representations were made with the program Diamond with all atoms displayed as 30% ellipsoids. Table 3 contains crystallographic data and details of measurements and refinement for compounds 4–6, 8, 10, 11. CCDC 2034611–2034616 contain the supplementary crystallographic data for 11, 6, 8, 10, 5, and 4, respectively.

### Measurement of cytostatic effect by MTT assay

*In vitro* cytostatic effect of the compounds was studied on A2058 human melanoma,^55^ A431 human epidermoid carcinoma,^56^ U87 human glioma,^57^ HepG2 human hepatocellular carcinoma^58^ and prostatic adenocarcinoma^58^ cells. A2058 and HepG2 cells were cultured in RPMI-1640 medium supplemented with 10% FCS (fetal calf serum, Sigma Ltd.), 2 mM l-glutamine, penicillin–streptomycin antibiotics mixture (50 IU mL^−1^ and 50 μg mL^−1^, respectively). A431, U87 and PC-3 cells were cultured in DMEM medium supplemented with 10% FBS, 2 mM l-glutamine, penicillin–streptomycin antibiotics mixture (50 IU mL^−1^ and 50 μg mL^−1^, respectively), 1 mM sodium pyruvate and 1% non-essential amino acid mixture. The cultures were maintained at 37 °C in a humidified atmosphere with 5% CO_2_.

Protocol 1: the cells were grown to confluency and were distributed into 96-well tissue culture plates with an initial cell number of 5.0 × 10^3^ per well. After 24 h of incubation at 37 °C, the cells were treated with the compounds in 200 μL final volume containing 1.0 v/v% DMSO. The cells were incubated with the compounds at 0.4–50 μM concentration range for 20 h, whereas control cells were treated with serum-free medium (RPMI-1640) only or with DMSO (*c* = 1.0 v/v%) at 37 °C for 20 h. After incubation, the cells were washed twice with serum-free RPMI-1640 medium. To determine the *in vitro* cytostatic effect, the cells were further cultured for 72 hours in 10% serum-containing medium. 3-(4,5-dimethylthiazol-2-yl)-2,5-diphenyltetrazolium bromide solution, MTT-solution, (45 μL, 2 mg mL^−1^, final concentration: 0.37 mg mL^−1^) was added to each well. The respiratory chain^60,61^ and other electron transport systems^62^ reduce MTT and thereby form non-water-soluble violet formazan crystals within the cell.^63^

The amount of these crystals can be determined spectrophotometrically and serves as an estimate for the number of mitochondria and hence the number of living cells in the well.^64^ After 3 hours of incubation the cells were centrifuged for 5 minutes at 900 g and the supernatant was removed. The obtained formazan crystals were dissolved in DMSO (100 mL) and optical density (OD) of the samples was measured at *λ* = 540 and 620 nm, respectively, using ELISA Reader (iEMS Reader, Labsystems, Finland). OD_620_ values were subtracted from OD_540_ values. The percent of cytostasis was calculated by using the following equation:Cytostatic effect (%) = [1 − (OD_treated_/OD_control_)] × 100

Values OD_treated_ and OD_control_ correspond to the optical densities of the treated and the control cells, respectively. In each case, two independent experiments were carried out with 4 parallel measurements. The 50% inhibitory concentration (IC_50_) values were determined from the dose-response curves. The curves were defined using Microcal™ Origin2018 software: cytostasis was plotted as a function of concentration, fitted to a sigmoidal curve, and based on this curve, the half-maximal inhibitory concentration (IC_50_) value was determined. IC_50_ represents the concentration of a compound that is required for 50% inhibition *in vitro* and expressed in micromolar units.

Protocol 2 and 3: cells were divided into 96 well tissue-culture plates in 200 μL culture medium with the initial cell number of 5000 cells per well. The compounds were dissolved in DMSO and then diluted with fresh culture medium (final DMSO concentration was 1% in each well) and they were added to the cells at 0.016, 0.08, 0.4, 2.0 and 10 μM final concentration. Cells were incubated with the compounds at 37 °C for 72 hours (protocol 2). The same layout of plates was parallelly treated – culture medium was removed, then compounds dissolved in medium containing 2.5% FBS were added to the wells, without washing – 3 times, in every 24 hours (protocol 3). After that, cell viability was determined by MTT-assay using 0.37 mg mL^−1^ final concentration of MTT, in each well. After 3 hours of incubation with MTT the absorbance was measured with ELISA-reader (Labsystems MS Reader) at 540 nm and 620 nm as reference wavelengths. IC_50_ values were determined from the dose-response curves using the same method as described in Protocol 1.

## Conclusions

In summary, we have demonstrated a flexible route for the synthesis of 1,2,4-thiadiazole hybrids by cross-coupling halogen derivatives of 1,2,4-thiadiazoles with erlotinib, ethynylferrocene, phenylacetylene, or ferroceneboronic acid, as well as investigated the *in vitro* antiproliferative and cytotoxic activity of these compounds on five tumorous cell lines (U87, A2058, A431, HepG2 and PC-3). The structures of all investigated compounds were confirmed by NMR, IR and mass spectroscopy, as well as single crystal X-ray diffraction. Ten compounds of the investigated fourteen exhibited cytostatic effect against at least one of the investigated cell lines with IC_50_ value below 50 μM. Three compounds, 3, 13, and 14/15, exhibited marked cytostatic effect against A431 cells with IC_50_ values of 5.5, 5.8 and 5.6 μM, respectively. 3, 13, and especially 14/15 were effective against PC-3 cells with IC_50_ values of 3.1, 4.5 and 0.4 μM, respectively. The isomer mixture 14/15 revealed outstanding antiproliferative effect against U87, A2058, and HepG2 cells showing IC_50_ values of 1.7, 1.2, and 1.6 μM, respectively. These compounds may serve as new leads for developing new anticancer agents.

## Conflicts of interest

There are no conflicts to declare.

## Supplementary Material

RA-011-D1RA05095H-s001

RA-011-D1RA05095H-s002
