# Supplementary material for: Synthesis, structure and in vitro antiproliferative effects of alkyne-linked 1,2,4-thiadiazole hybrids including erlotinib- and ferrocene-containing derivatives
Source: RSC Adv. 2021 Aug 25;11(46):28685–97. doi: 10.1039/d1ra05095h (PMC9038148; doi:10.1039/d1ra05095h)
Supplement: RA-011-D1RA05095H-s001 [file RA-011-D1RA05095H-s001.pdf]

Electronic Supplementary Material (ESI) for RSC Advances.
This journal is © The Royal Society of Chemistry 2021

Synthesis, structure and *in vitro* antiproliferative effect of alkyne-linked 1,2,4-thiadiazole hybrids including Erlotinib- and ferrocene-containing derivatives

Mohammed Boulhaoua,[a] Tibor Pasinszki,[*b] Ana Torvisco,[c] Rita Oláh-szabó,[d] Szilvia Bősze [d] and Antal Csámpai [*e]

[a] *ELTE Eötvös Loránd University, Institute of Chemistry, Department of Inorganic Chemistry, H-1117 Budapest, Hungary.*

[b] *Fiji National University, College of Engineering, Science and Technology, Department of Chemistry, P.O.Box 3722, Samabula, Suva, Fiji.*

[c] *Graz University of Technology, Institute of Inorganic Chemistry, Stremayrgasse 9/V, 8010 Graz, Austria.*

[d] *MTA-ELTE Research Group of Peptide Chemistry, Pázmány P. sétány 1/A, H-1117 Budapest, Hungary.*

[e] *ELTE Eötvös Loránd University, Institute of Chemistry, Department of Organic Chemistry, H-1117 Budapest, Hungary.*

[*] *Corresponding authors e-mail addresses: tibor.pasinszki@fnu.ac.fj, antal.csampai@ttk.elte.hu*

Supporting material

Content:

Table S1. Reaction of 3,5-dihalogeno-1,2,4-thiadiazoles (**2**,**3**) with ethynylferrocene page 2
Table S2. Reaction of 3,5-dihalogeno-1,2,4-thiadiazole (**2**,**3**) with erlotinib page 3
Table S3. Reaction of 3,5-dichloro-1,2,4-thiadiazole (**2**) with ferroceneboronic acid page 4
Figure S1−9. Mass spectra of compounds **4**, **5**, **6**, **8**, **10**, **11**, **12**, **13**, and **14/15** page 5–9
Figure S10. ATR-IR spectra of compounds **4**, **5**, **6**, **8**, **10**, **11**, **12**, **13**, and **14/15** page 10
Figure S11−16. Structure and crystal packing of **4**, **5**, **6**, **8**, **10**, and **11** page 11–16
Table S4-6. Geometric parameters of **4, 5,** and **6** page 17−26
Table S7-9. Geometric parameters of **8, 10,** and **11** page 27−36
Table S10. van der Waals radii (r_w) and values of experimental halide interactions page 37
Table S11. Intermolecular interactions of **4**, **5**, and **6** page 37
Table S12. Intermolecular interactions of **8**, **10**, and **11** page 38
Figure S17-22. ^{1}H NMR spectra of compounds **4**, **5**, **6**, **8**, **10**, **13** page 39−41
Figure S23-30. ^{13}C NMR spectra of compounds **4**, **5**, **6**, **8**, **10**, **11**, **13**, and **14/15** page 42−45
Figure S31−34. HMBC NMR spectra of compounds **8**, **10**, **13**, and **14/15** page 46−47
Figure S35−37. HSQC NMR spectra of compounds **8**, **10**, and **14/15** page 48−49
Figure S38−43. HR-MS spectra of compounds **12**, **13**, and **14/15** page 50−55

Table S1: Sonogashira coupling reaction of 3,5-dihalogeno-1,2,4-thiadiazole with ethynylferrocene

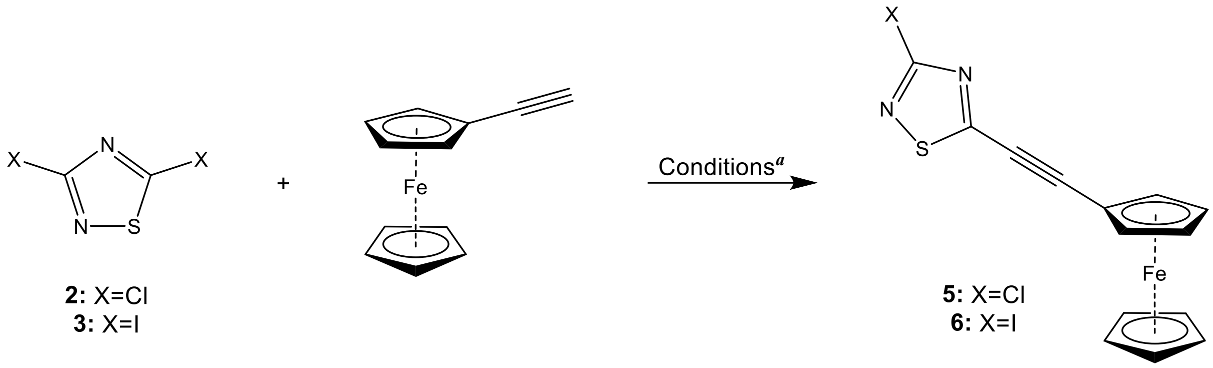

Entry	X	Catalyst	Base	Solvent	Temp (°C)	Yield (%)[b]
**1**	I	$Pd(PPh_3)_4$/CuI	K_3PO_4	THF	50	0
**2**	I	$PdCl_2(PPh_3)_2$/CuI	K_3PO_4	THF	50	15
**3**	I	$PdCl_2(PPh_3)_2$/CuI	Et_3N	THF	50	44
**4**	I	$PdCl_2(PPh_3)_2$/CuI	Et_3N	Et_3N	50	62
**5**	I	$PdCl_2(PPh_3)_2$/CuI	Et_3N	Toluene	50	70
**6**	I	$PdCl_2(PPh_3)_2$/CuI	$NH(i\text{-}Pr)_2$	Toluene	50	87
**7**	Cl	$PdCl_2(PPh_3)_2$/CuI	$NH(i\text{-}Pr)_2$	Toluene	50	82

[a] Reaction conditions: A mixture of **2** or **3** (1 mmol), ethynylferrocene (1.1 mmol), catalyst (3 mol%), CuI (3 mol%), and base (1.1 mmol) was reacted in THF, Et_3N, or toluene (5 ml) at 50 °C for 6 h under a nitrogen atmosphere.
[b] Isolated yield.

Table 2: Sonogashira coupling reaction of 3,5-dihalogeno-1,2,4-thiadiazole with erlotinib

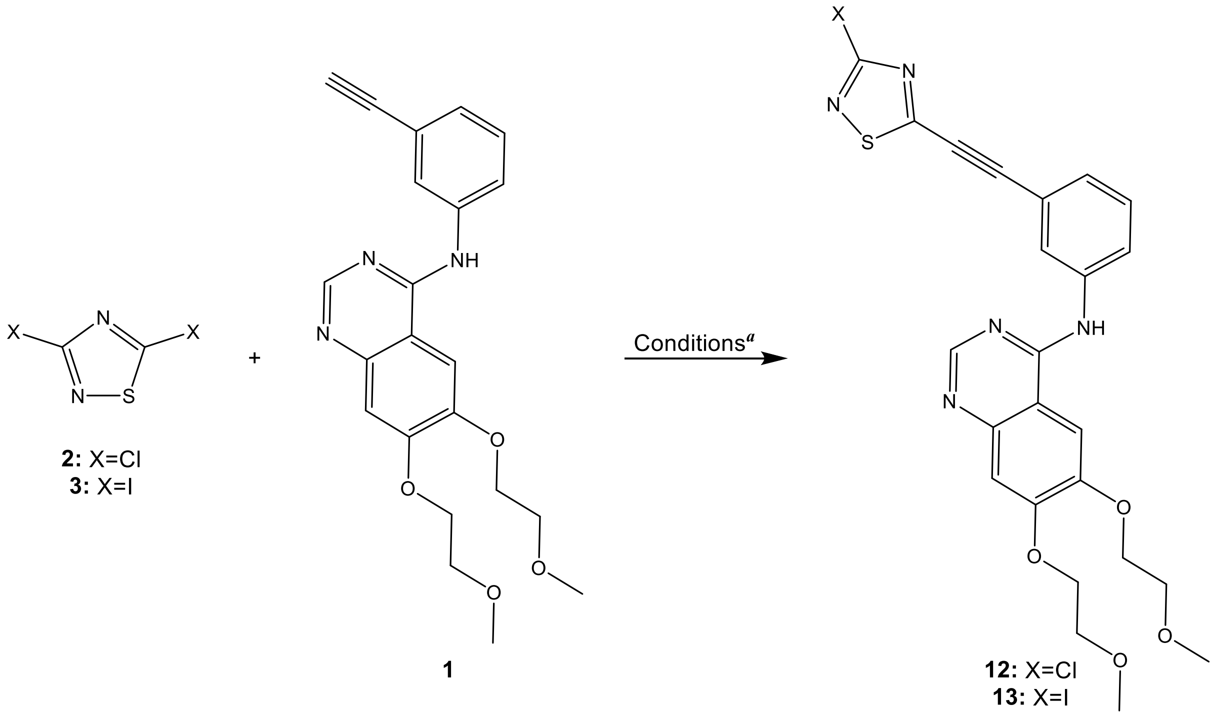

Entry	X	Catalyst	Base	Solvent	Temp (°C)	Yield (%)[b]
1	I	$PdCl_2(PPh_3)_2$/CuI	DIPA	Toluene	50	0
2	I	$PdCl_2(PPh_3)_2$/CuI	DIPA	Toluene	80	13
3	I	$PdCl_2(PPh_3)_2$/CuI	DIPA	DMF	80	22
4	I	$PdCl_2(PPh_3)_2$/CuI	K_3PO_4	DMF	80	48
5	I	$Pd[P(t\text{-}Bu)_3]_2$/CuI	K_3PO_4	DMF	80	75
6	Cl	$Pd[P(t\text{-}Bu)_3]_2$/CuI	K_3PO_4	DMF	80	71

[a] Reaction conditions: A mixture of **2** or **3** (1 mmol), **1** (1.1 mmol), catalyst (10 mol%), CuI (10 mol%), and base (1.1 mmol) was reacted in toluene or DMF (3 ml) at 80 °C for 12 h under a nitrogen atmosphere.

[b] Isolated yield.

Table 3: Suzuki coupling reaction of 3,5-dichloro-1,2,4-thiadiazole with ferroceneboronic acid

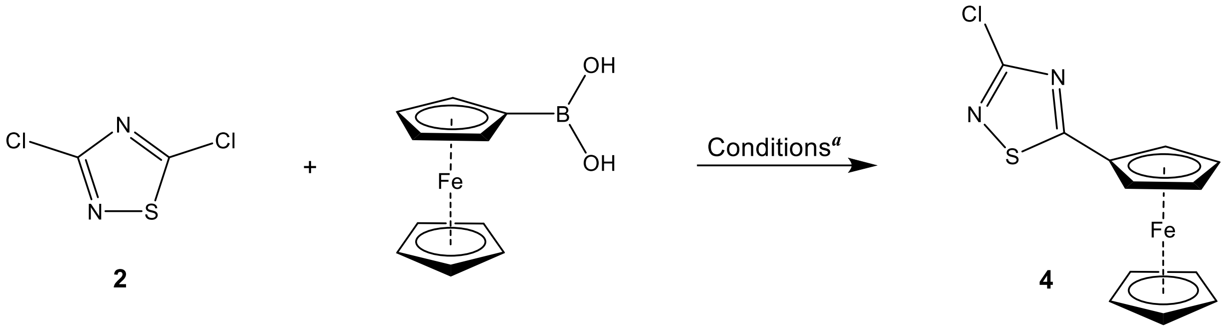

Entry	Catalyst	Ligand	Base	Solvent	Temp (°C)	Yield (%)[b]
1	$Pd(PPh_3)_4$	-	K_2CO_3	Toluene	Reflux	0
2	$Pd(PPh_3)_4$	-	K_2CO_3	Dioxane	Reflux	0
3	$PdCl_2(PPh_3)_2$	-	K_2CO_3 (2M)	Toluene	RT	0
4	$PdCl_2(PPh_3)_2$	-	K_2CO_3 (2M)	Toluene	Reflux	22
5	$PdCl_2(PPh_3)_2$	-	K_2CO_3	THF	Reflux	0
6	$PdCl_2(PPh_3)_2$	-	$KHCO_3$	Dioxane/H_2O (4 :1)	Reflux	0
7	$PdCl_2(PPh_3)_2$	-	K_2CO_3	Dioxane	Reflux	48
6	$Pd(dppf)Cl_2$	-	K_2CO_3	Dioxane	Reflux	56
7	$Pd(OAc)_2$	PPh_3	K_2CO_3	Dioxane	Reflux	78

[a] Reaction conditions: a mixture of **2** (1 mmol), ferroceneboronic acid (1.5 mmol), catalyst (5 mol%), PPh_3 (15 mol%), and base (3 mmol) was refluxed in toluene, THF, or dioxane (5 ml) for 14 h under a nitrogen atmosphere.
[b] Isolated yield.

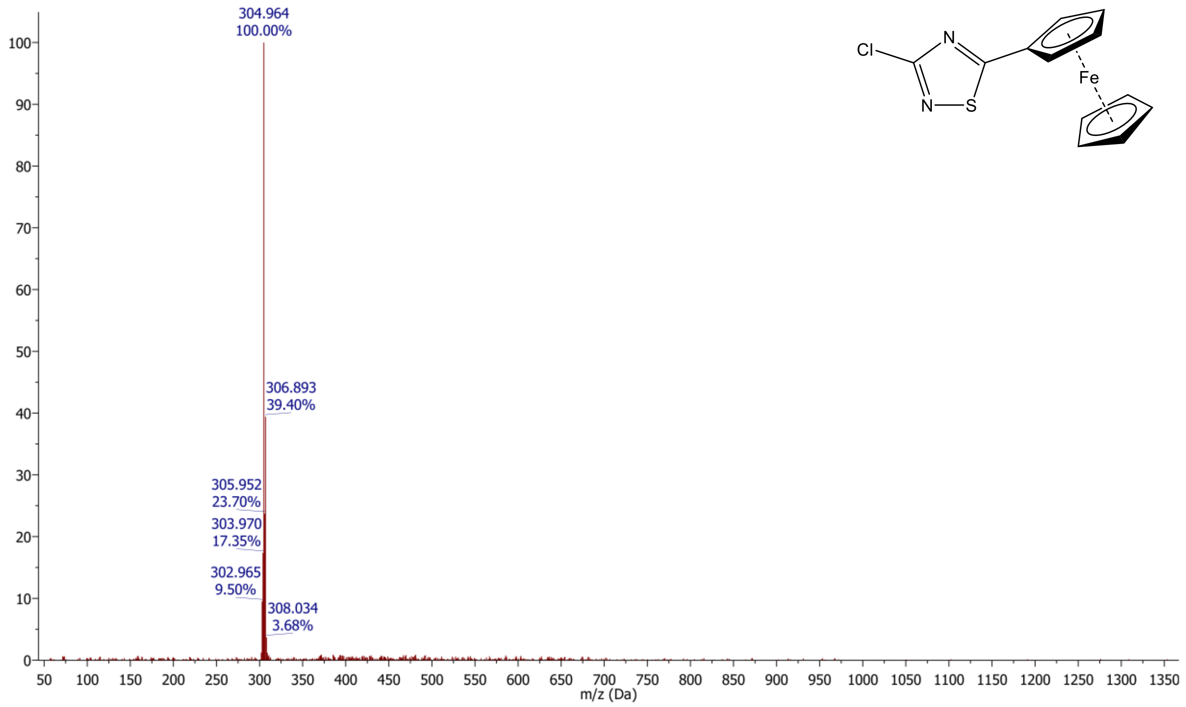

Figure S1. Mass spectrum of **4** (mass of the most abundant isotope: 303.95 Da)

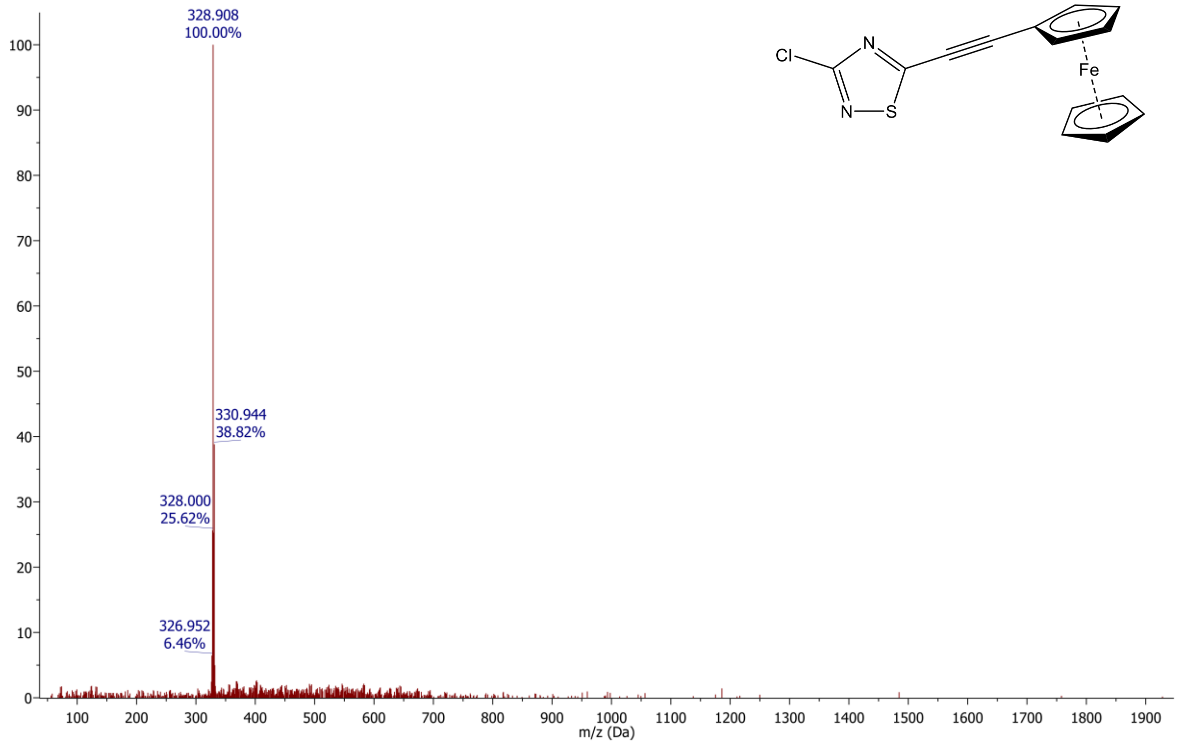

Figure S2. Mass spectrum of **5** (mass of the most abundant isotope: 327.95 Da)

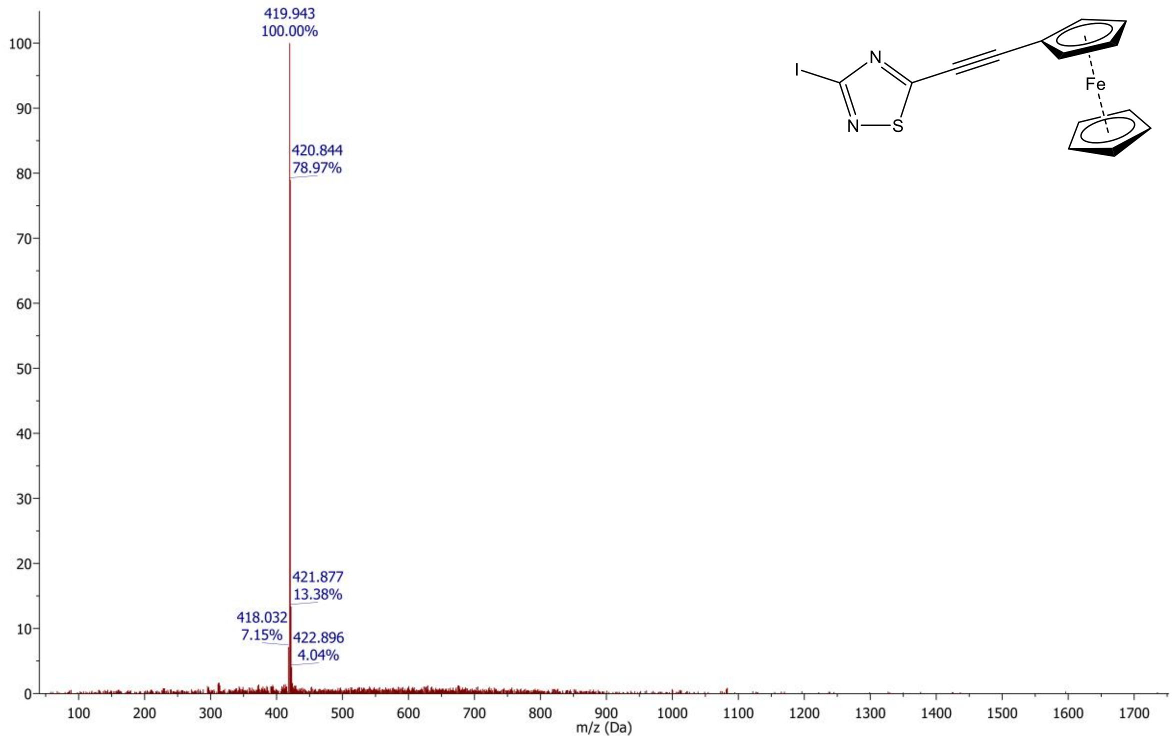

Figure S3. Mass spectrum of **6** (mass of the most abundant isotope: 419.89 Da)

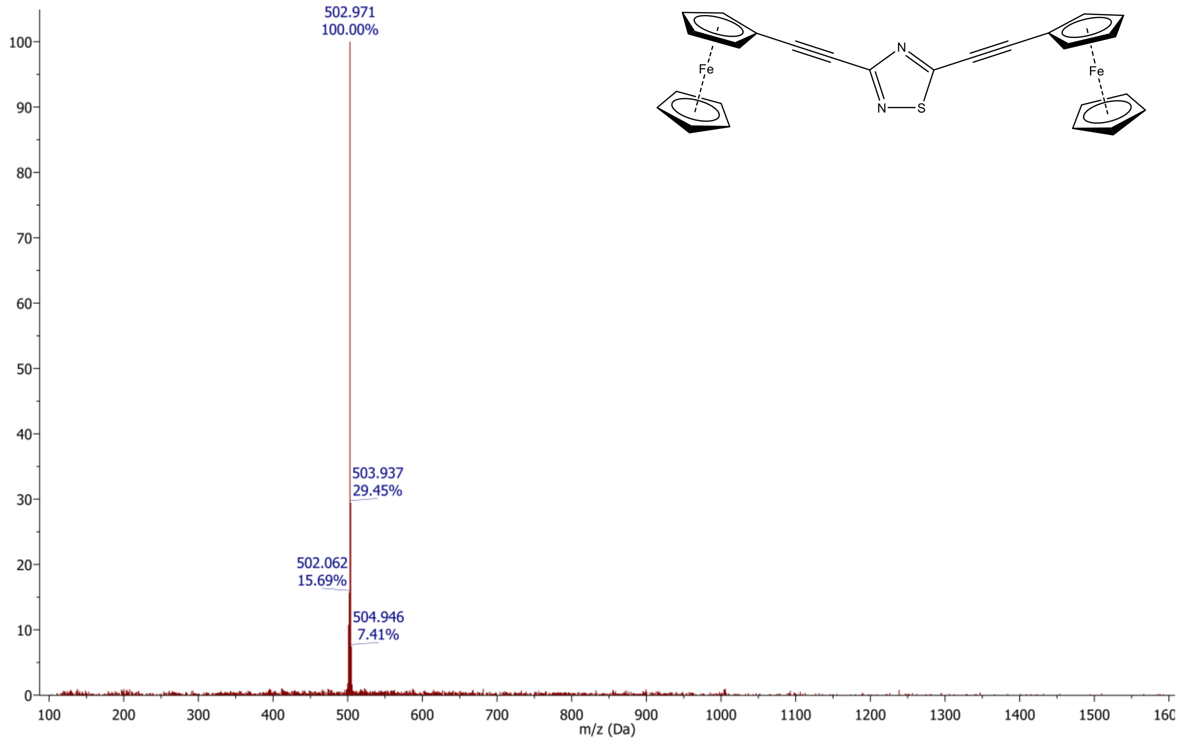

Figure S4. Mass spectrum of **8** (mass of the most abundant isotope: 501.99 Da)

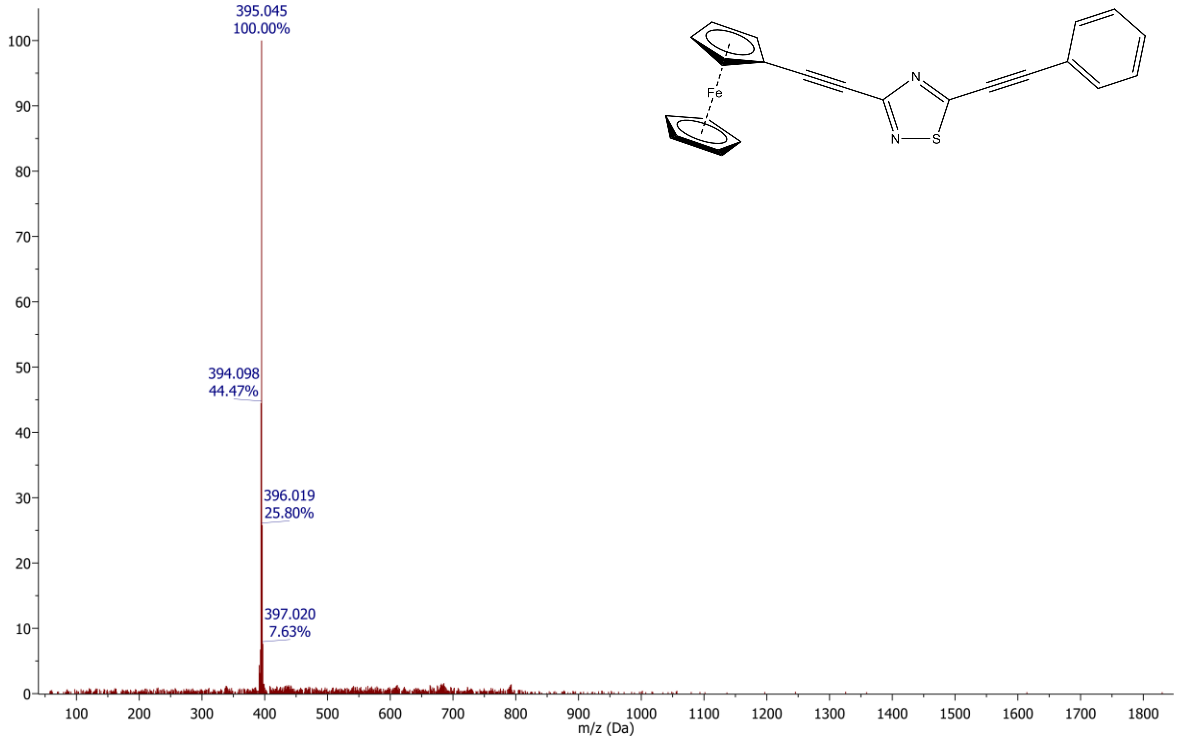

Figure S5. Mass spectrum of **10** (mass of the most abundant isotope: 394.02 Da)

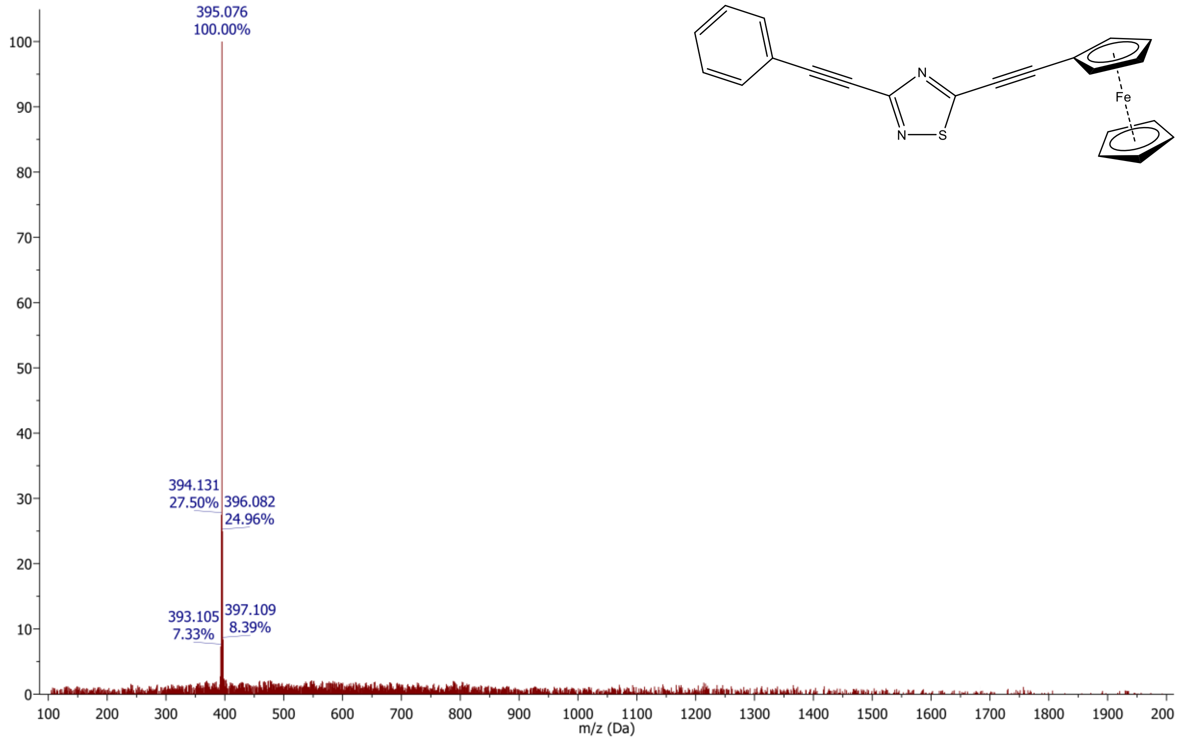

Figure S6. Mass spectrum of **11** (mass of the most abundant isotope: 394.02 Da)

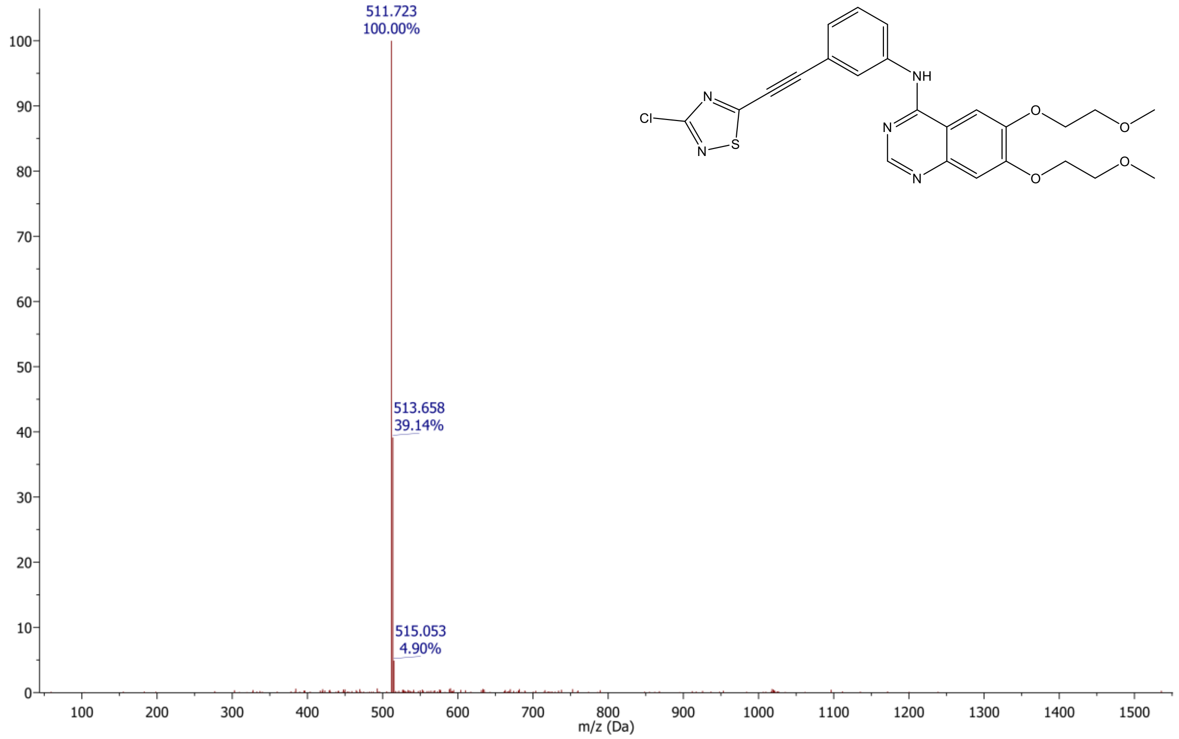

Figure S7. Mass spectrum of **12** (mass of the most abundant isotope: 511.11 Da)

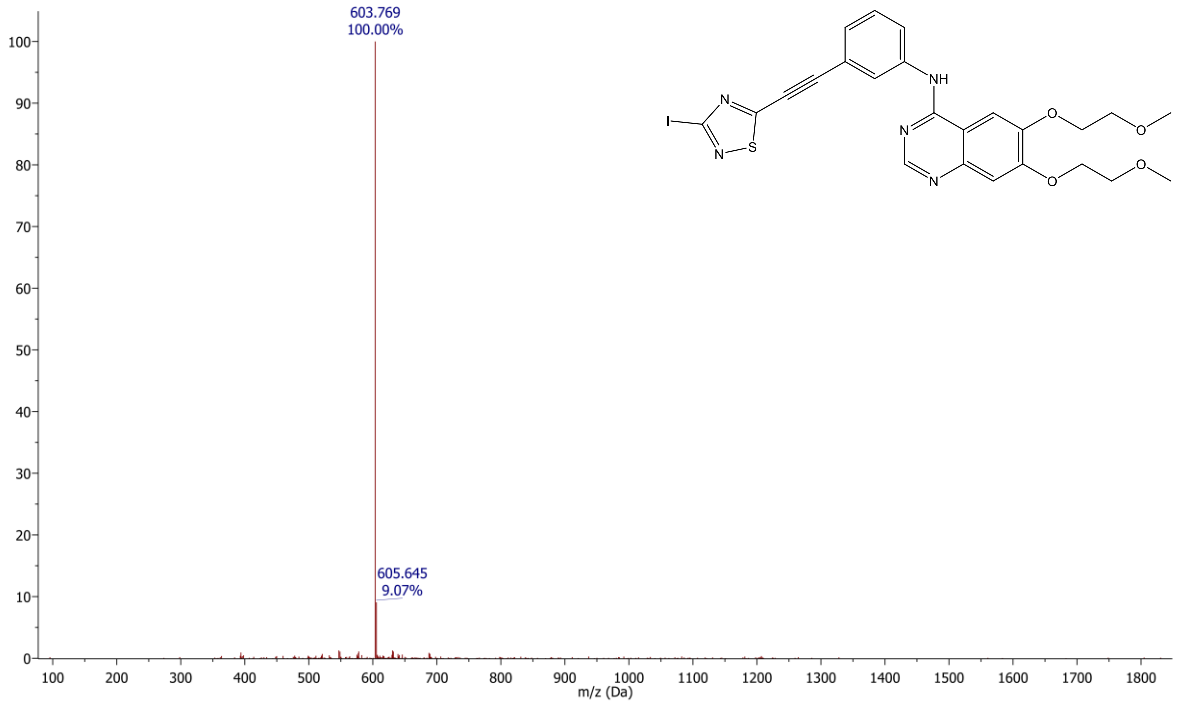

Figure S8. Mass spectrum of **13** (mass of the most abundant isotope: 603.04 Da)

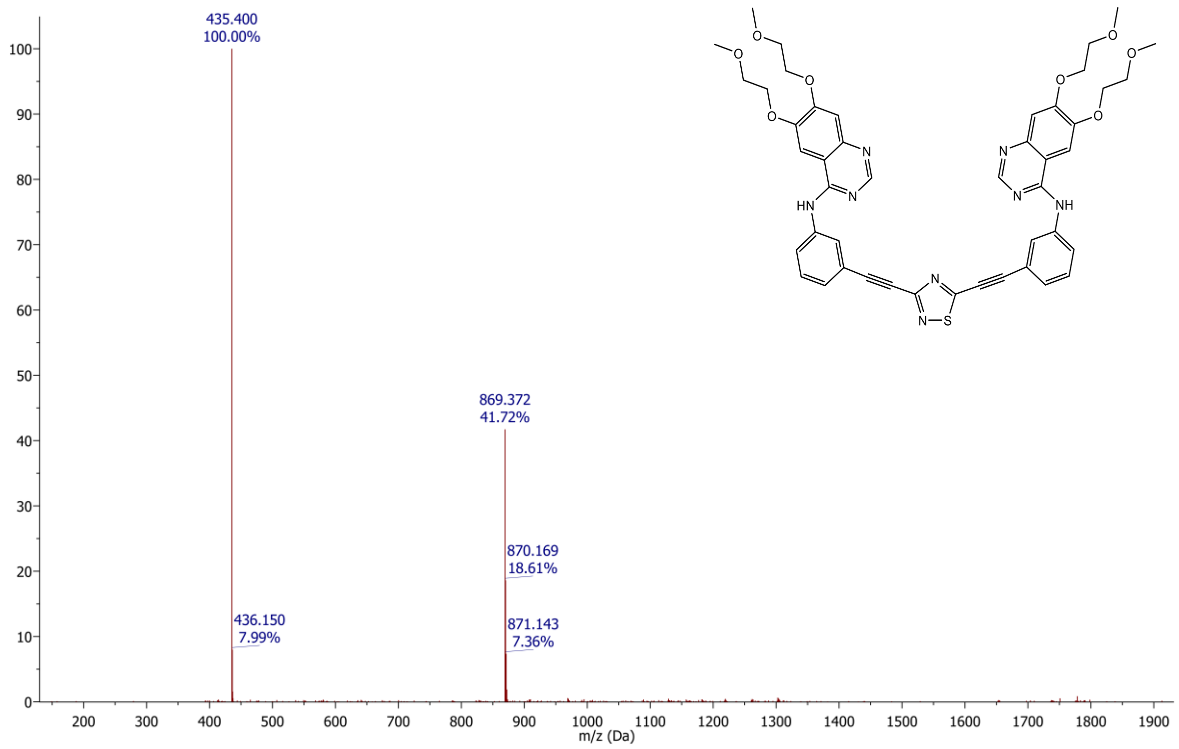

Figure S9. Mass spectrum of **14**/**15** (mass of the most abundant isotope: 868.30 Da)

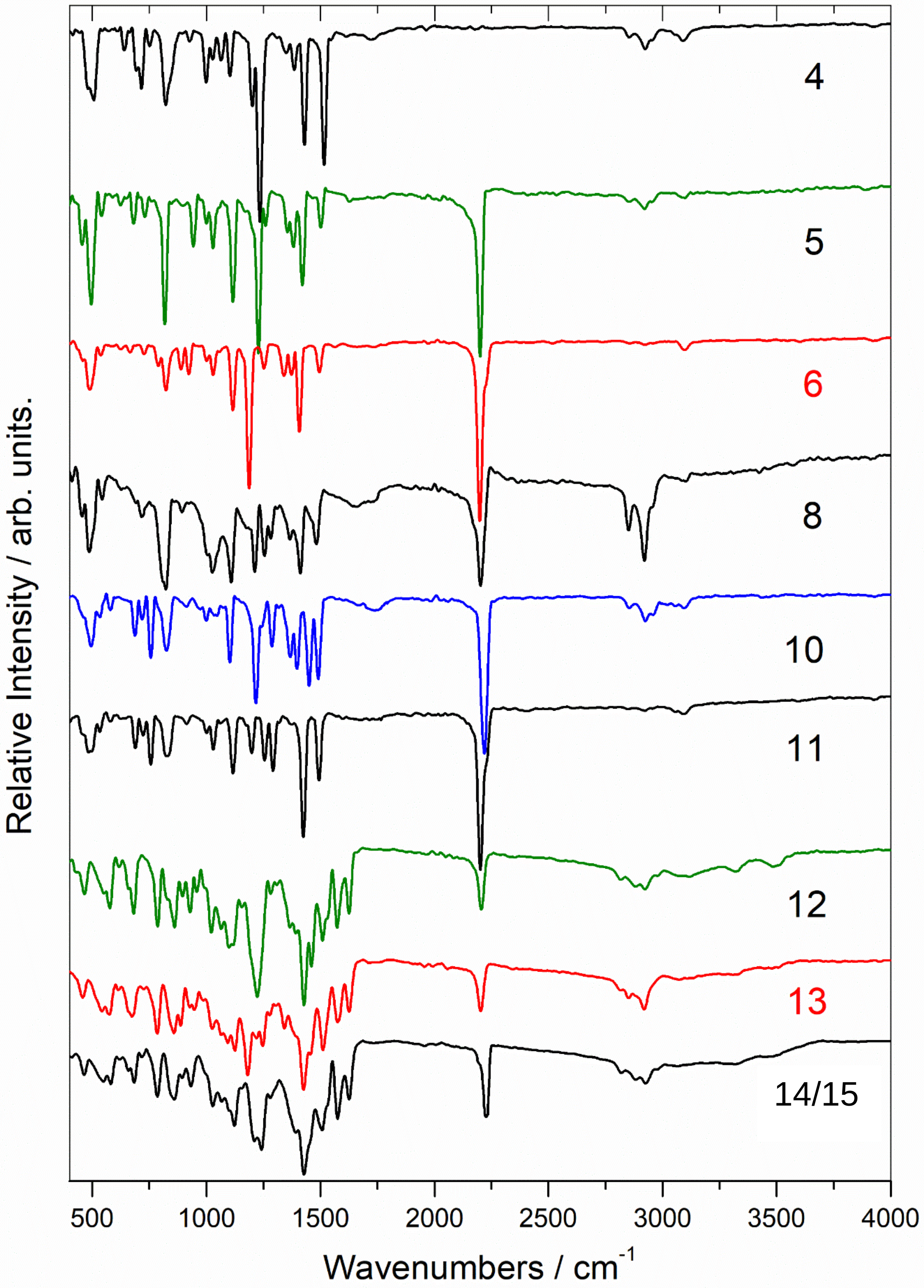

Figure S10. ATR-FTIR spectra of synthesized compounds (neat, solid)

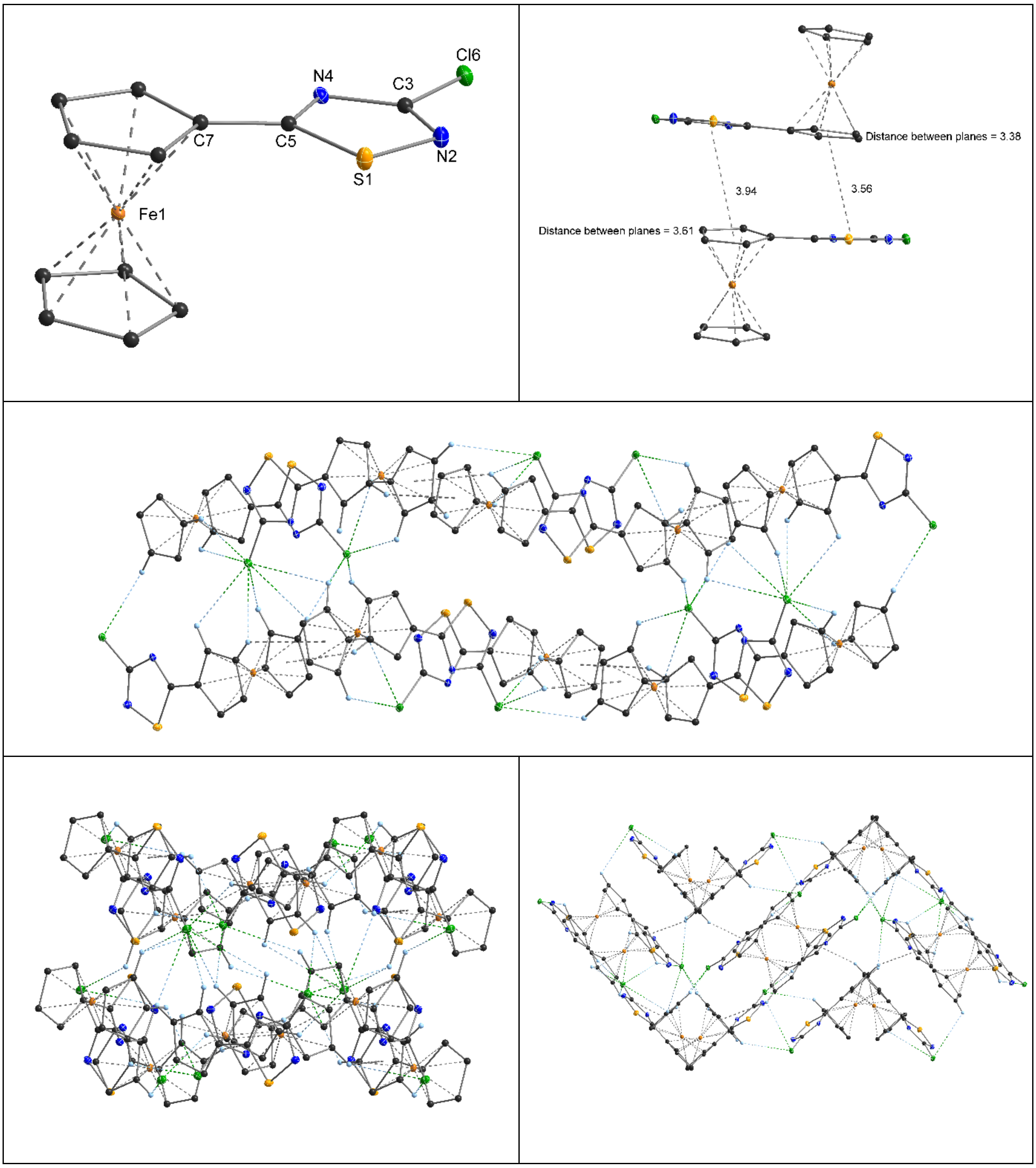

Figure S11. Structure and crystal packing of **4**. All atoms are shown as 30% shaded ellipsoids.

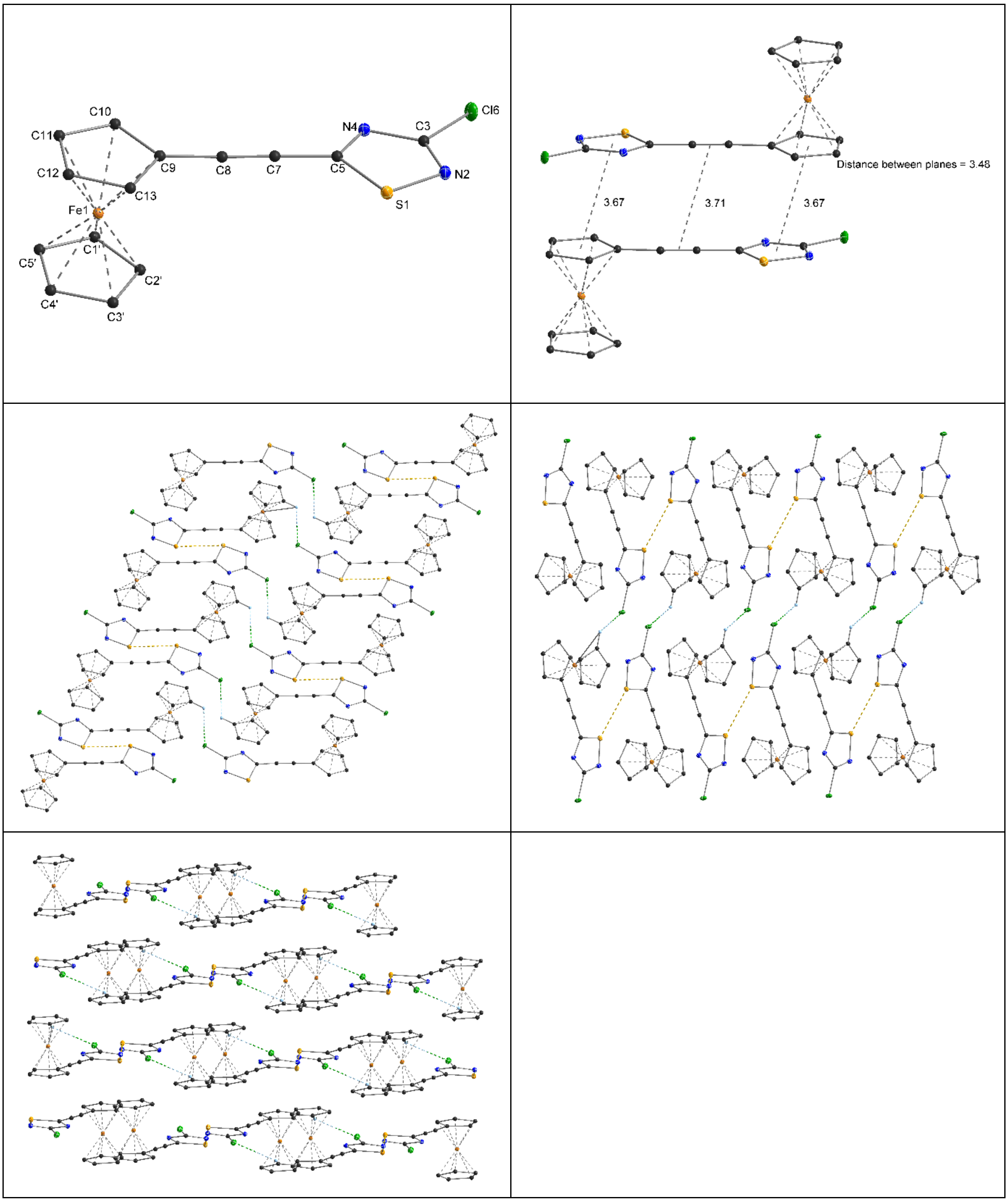

Figure S12. Structure and crystal packing of **5**. All atoms are shown as 30% shaded ellipsoids.

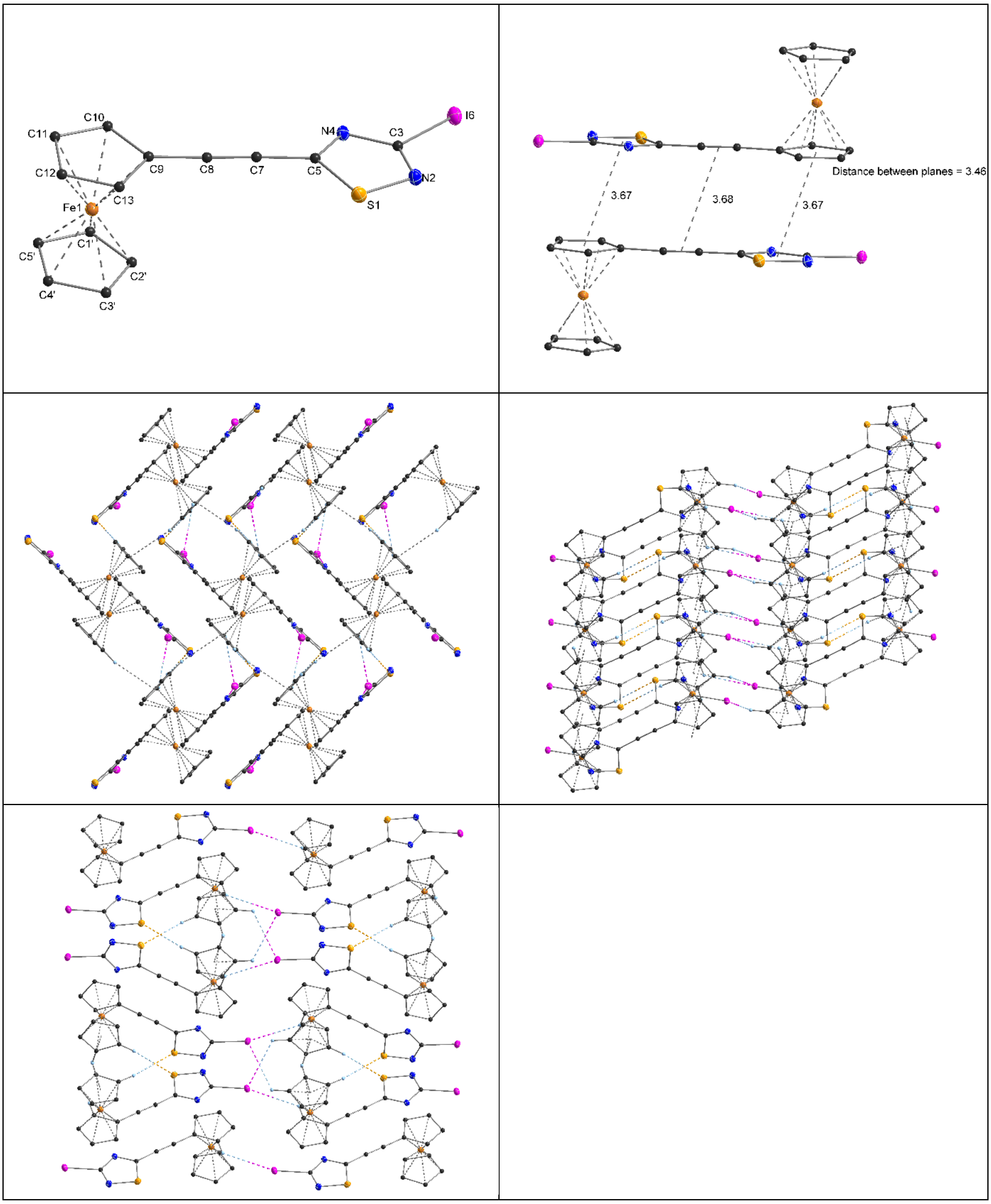

Figure S13. Structure and crystal packing of **6**. All atoms are shown as 30% shaded ellipsoids.

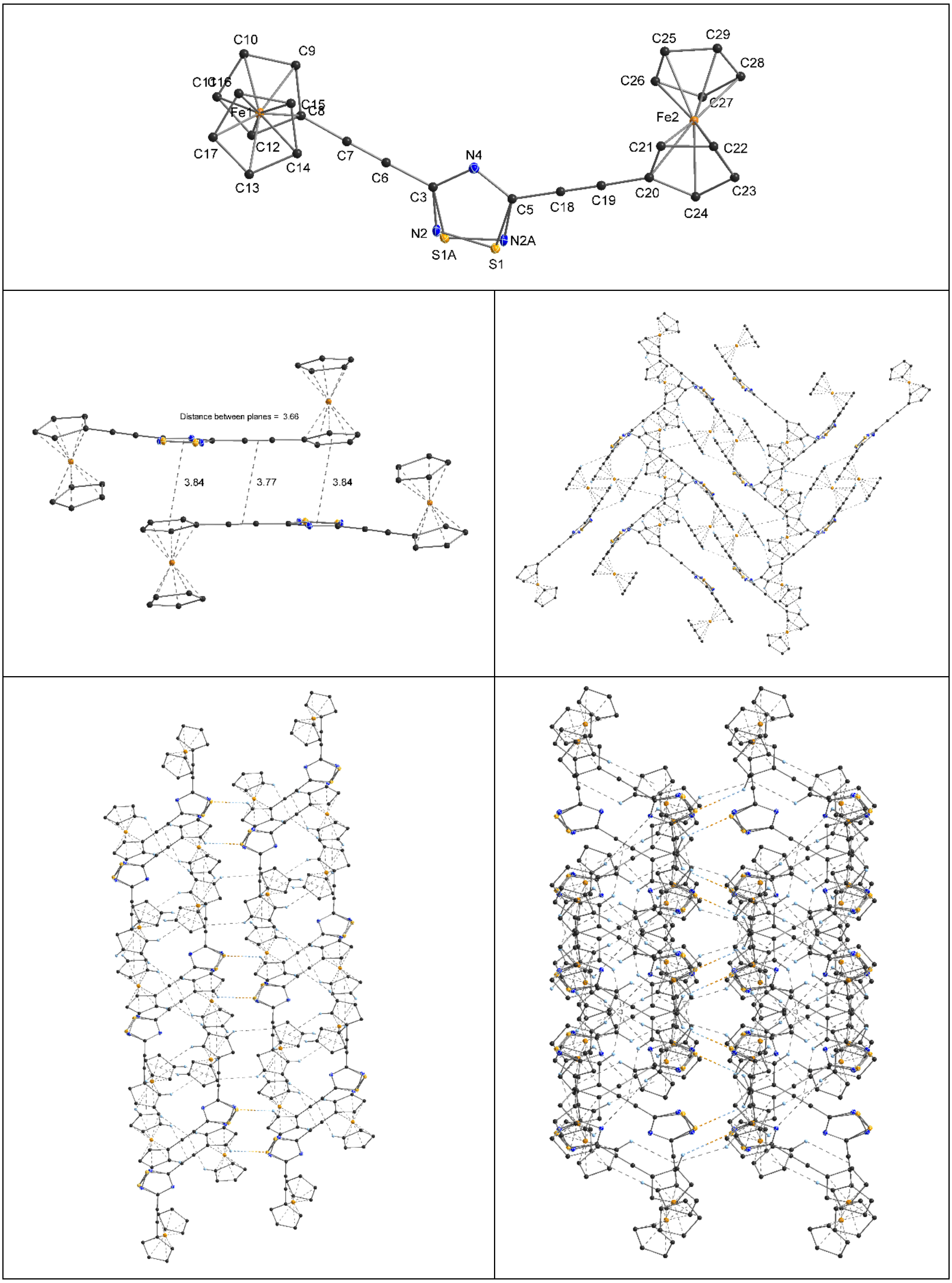

Figure S14. Structure and crystal packing of **8**. All atoms are shown as 30% shaded ellipsoids.

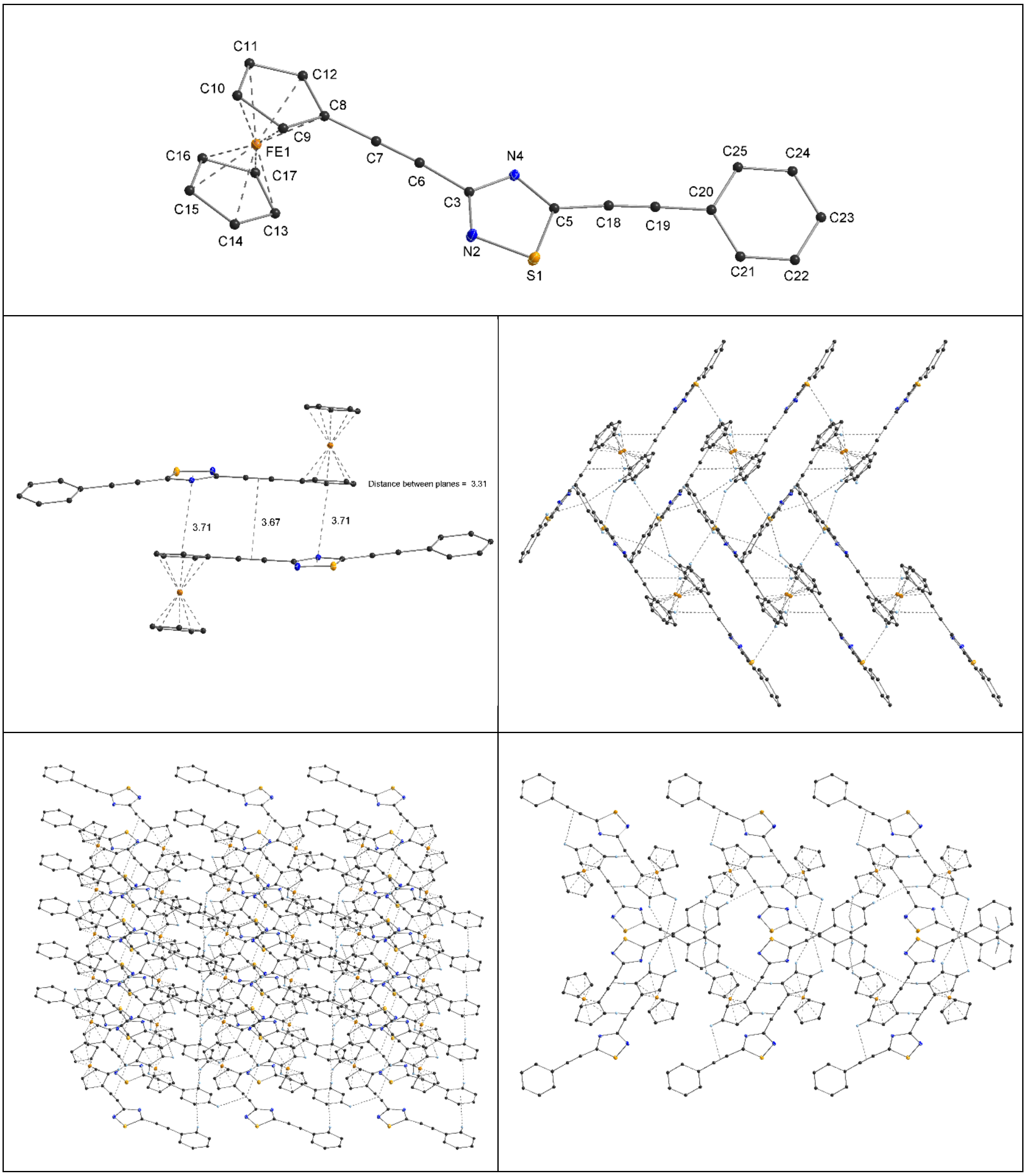

Figure S15. Structure and crystal packing of **10**. All atoms are shown as 30% shaded ellipsoids. MPF

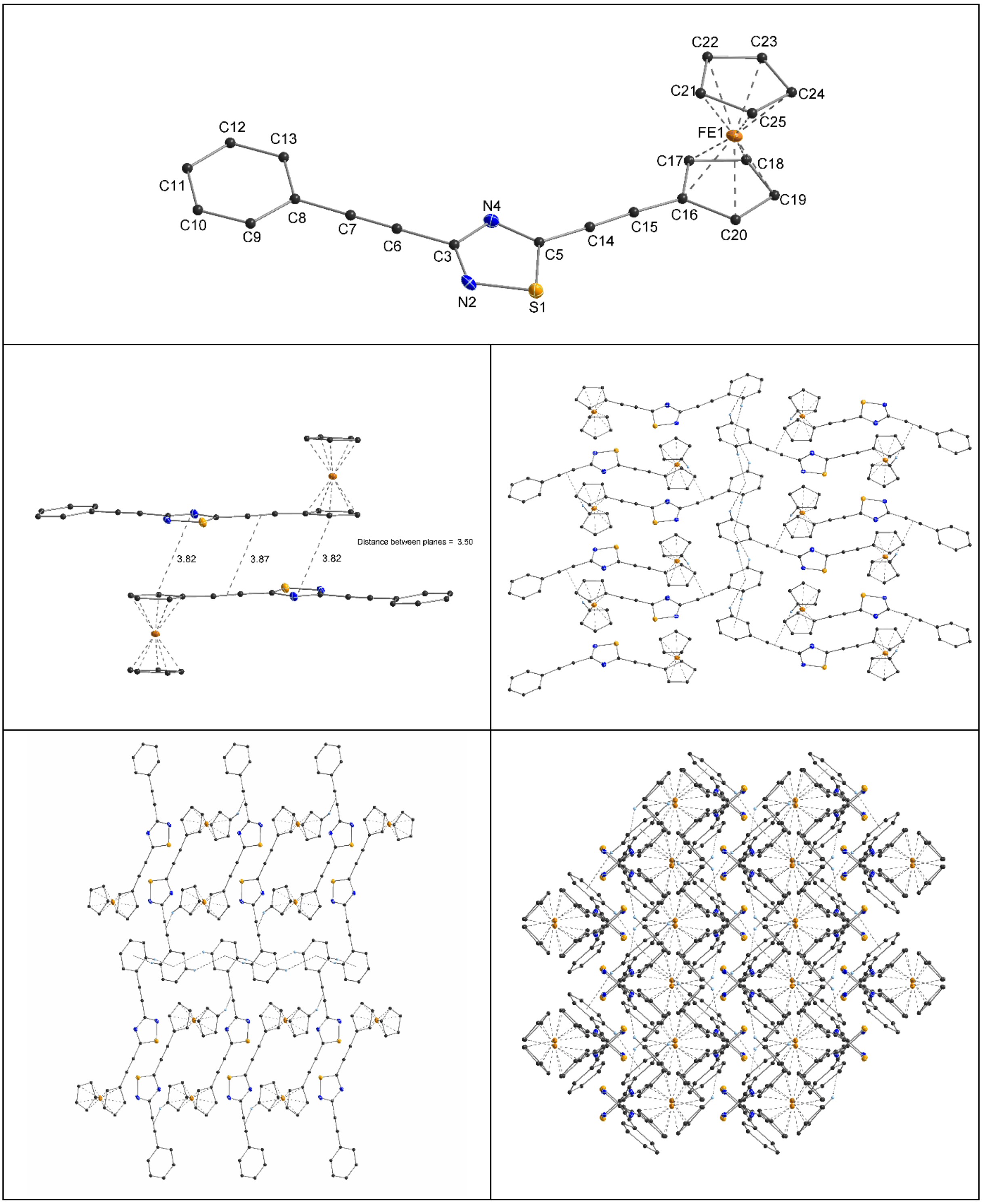

Figure S16. Structure and crystal packing of **11**. All atoms are shown as 30% shaded ellipsoids. MFA

Table 4. Geometric parameters of **4** (bond lengths in Å and bond angles in °)

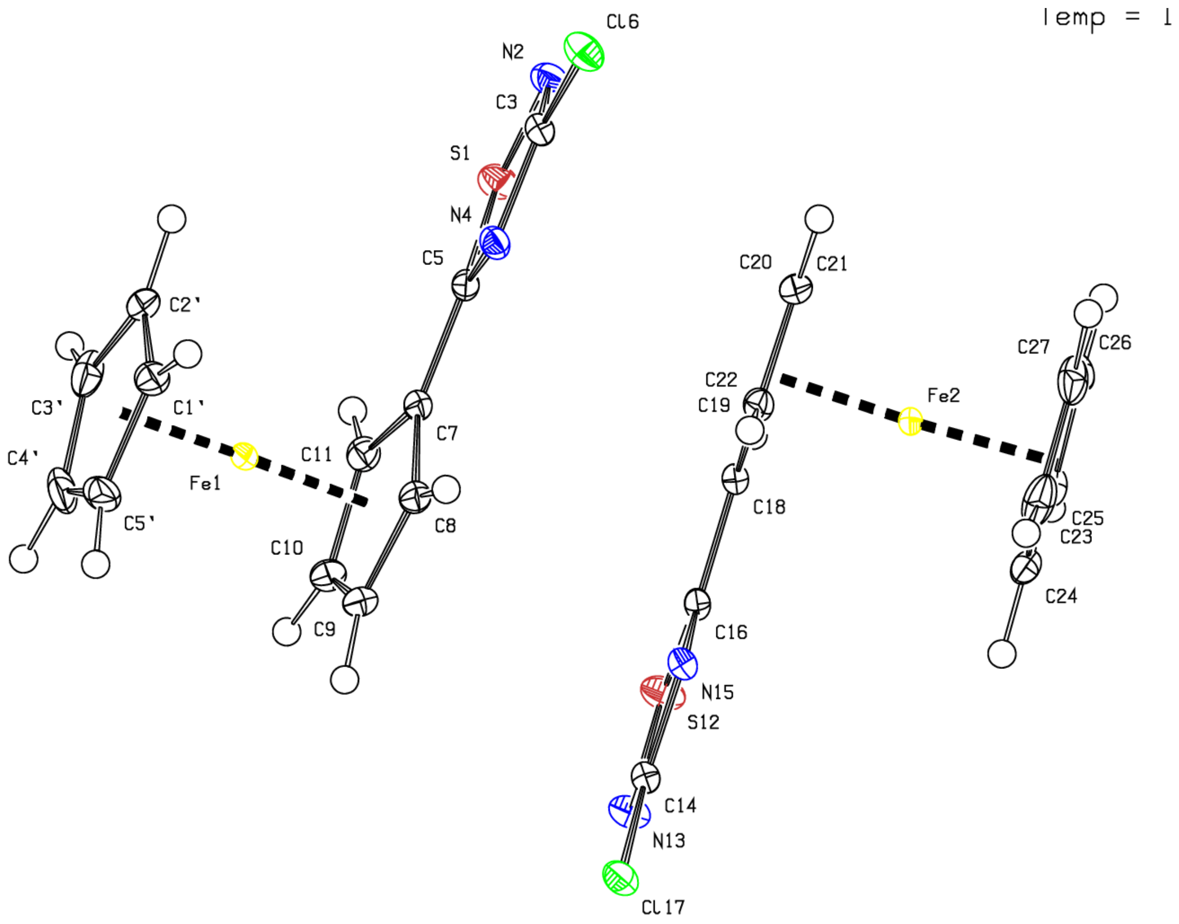

Fe1—C7	2.0362 (11)	Fe2—C27	2.0389 (12)
Fe1—C8	2.0452 (12)	Fe2—C18	2.0408 (11)
Fe1—C11	2.0474 (11)	Fe2—C19	2.0411 (11)
Fe1—C4'	2.0497 (12)	Fe2—C26	2.0424 (11)
Fe1—C1'	2.0520 (12)	Fe2—C22	2.0451 (11)
Fe1—C5'	2.0533 (12)	Fe2—C23	2.0502 (12)
Fe1—C2'	2.0559 (11)	Fe2—C21	2.0551 (11)
Fe1—C10	2.0573 (12)	Fe2—C20	2.0552 (11)
Fe1—C3'	2.0575 (12)	Fe2—C24	2.0560 (12)
Fe1—C9	2.0594 (12)	Fe2—C25	2.0602 (11)
S1—N2	1.6635 (11)	S12—N13	1.6608 (11)
S1—C5	1.7267 (11)	S12—C16	1.7240 (11)
N2—C3	1.3089 (15)	N13—C14	1.3077 (15)
C3—N4	1.3589 (14)	C14—N15	1.3601 (14)
C3—Cl6	1.7209 (12)	C14—Cl17	1.7199 (12)
N4—C5	1.3192 (14)	N15—C16	1.3249 (15)
C5—C7	1.4487 (15)	C16—C18	1.4483 (15)
C7—C8	1.4368 (15)	C18—C19	1.4382 (15)
C7—C11	1.4389 (16)	C18—C22	1.4403 (16)
C8—C9	1.4243 (17)	C19—C20	1.4235 (16)
C8—H8	1.0000	C19—H19	1.0000

C9—C10	1.4268 (19)	C20—C21	1.4278 (17)
C9—H9	1.0000	C20—H20	1.0000
C10—C11	1.4272 (17)	C21—C22	1.4269 (16)
C10—H10	1.0000	C21—H21	1.0000
C11—H11	1.0000	C22—H22	1.0000
C1'—C5'	1.4248 (17)	C23—C27	1.422 (2)
C1'—C2'	1.4271 (17)	C23—C24	1.429 (2)
C1'—H1'	1.0000	C23—H23	1.0000
C2'—C3'	1.4225 (17)	C24—C25	1.4234 (17)
C2'—H2'	1.0000	C24—H24	1.0000
C3'—C4'	1.424 (2)	C25—C26	1.4233 (17)
C3'—H3'	1.0000	C25—H25	1.0000
C4'—C5'	1.423 (2)	C26—C27	1.4175 (18)
C4'—H4'	1.0000	C26—H26	1.0000
C5'—H5'	1.0000	C27—H27	1.0000
C7—Fe1—C8	41.22 (4)	C27—Fe2—C18	155.14 (5)
C7—Fe1—C11	41.26 (5)	C27—Fe2—C19	118.04 (5)
C8—Fe1—C11	69.36 (5)	C18—Fe2—C19	41.26 (4)
C7—Fe1—C4'	165.45 (6)	C27—Fe2—C26	40.65 (5)
C8—Fe1—C4'	151.72 (6)	C18—Fe2—C26	163.81 (5)
C11—Fe1—C4'	126.65 (5)	C19—Fe2—C26	152.84 (5)
C7—Fe1—C1'	119.48 (5)	C27—Fe2—C22	160.30 (5)
C8—Fe1—C1'	108.26 (5)	C18—Fe2—C22	41.28 (4)
C11—Fe1—C1'	153.61 (5)	C19—Fe2—C22	69.38 (5)
C4'—Fe1—C1'	68.15 (5)	C26—Fe2—C22	125.03 (5)
C7—Fe1—C5'	152.97 (5)	C27—Fe2—C23	40.71 (6)
C8—Fe1—C5'	118.13 (5)	C18—Fe2—C23	121.83 (5)
C11—Fe1—C5'	164.27 (5)	C19—Fe2—C23	106.61 (5)
C4'—Fe1—C5'	40.59 (6)	C26—Fe2—C23	68.35 (5)
C1'—Fe1—C5'	40.62 (5)	C22—Fe2—C23	158.39 (5)
C7—Fe1—C2'	108.71 (5)	C27—Fe2—C21	122.26 (5)
C8—Fe1—C2'	128.45 (5)	C18—Fe2—C21	68.75 (4)
C11—Fe1—C2'	119.11 (5)	C19—Fe2—C21	68.64 (5)
C4'—Fe1—C2'	68.21 (5)	C26—Fe2—C21	106.17 (5)
C1'—Fe1—C2'	40.66 (5)	C22—Fe2—C21	40.73 (4)
C5'—Fe1—C2'	68.44 (5)	C23—Fe2—C21	159.30 (6)
C7—Fe1—C10	68.73 (5)	C27—Fe2—C20	104.18 (5)
C8—Fe1—C10	68.68 (5)	C18—Fe2—C20	68.81 (4)
C11—Fe1—C10	40.69 (5)	C19—Fe2—C20	40.67 (5)
C4'—Fe1—C10	107.06 (5)	C26—Fe2—C20	118.04 (5)
C1'—Fe1—C10	164.82 (5)	C22—Fe2—C20	68.82 (5)
C5'—Fe1—C10	126.54 (5)	C23—Fe2—C20	122.78 (5)
C2'—Fe1—C10	152.60 (5)	C21—Fe2—C20	40.65 (5)
C7—Fe1—C3'	127.99 (5)	C27—Fe2—C24	68.59 (5)
C8—Fe1—C3'	166.41 (5)	C18—Fe2—C24	110.09 (5)

C11—Fe1—C3'	107.64 (5)	C19—Fe2—C24	126.12 (5)
C4'—Fe1—C3'	40.58 (6)	C26—Fe2—C24	68.45 (5)
C1'—Fe1—C3'	68.17 (5)	C22—Fe2—C24	123.55 (5)
C5'—Fe1—C3'	68.36 (6)	C23—Fe2—C24	40.73 (6)
C2'—Fe1—C3'	40.46 (5)	C21—Fe2—C24	157.76 (5)
C10—Fe1—C3'	118.27 (5)	C20—Fe2—C24	161.27 (5)
C7—Fe1—C9	68.59 (5)	C27—Fe2—C25	68.26 (5)
C8—Fe1—C9	40.61 (5)	C18—Fe2—C25	127.91 (5)
C11—Fe1—C9	68.59 (5)	C19—Fe2—C25	164.39 (5)
C4'—Fe1—C9	117.91 (5)	C26—Fe2—C25	40.60 (5)
C1'—Fe1—C9	127.65 (5)	C22—Fe2—C25	109.77 (5)
C5'—Fe1—C9	107.33 (5)	C23—Fe2—C25	68.11 (5)
C2'—Fe1—C9	166.01 (5)	C21—Fe2—C25	121.56 (5)
C10—Fe1—C9	40.56 (5)	C20—Fe2—C25	154.65 (5)
C3'—Fe1—C9	151.89 (5)	C24—Fe2—C25	40.46 (5)
N2—S1—C5	93.16 (5)	N13—S12—C16	93.06 (5)
C3—N2—S1	105.60 (8)	C14—N13—S12	105.95 (8)
N2—C3—N4	122.38 (10)	N13—C14—N15	122.23 (10)
N2—C3—Cl6	120.04 (9)	N13—C14—Cl17	119.59 (9)
N4—C3—Cl6	117.58 (9)	N15—C14—Cl17	118.18 (8)
C5—N4—C3	107.57 (9)	C16—N15—C14	107.31 (9)
N4—C5—C7	123.36 (10)	N15—C16—C18	123.63 (10)
N4—C5—S1	111.29 (8)	N15—C16—S12	111.44 (8)
C7—C5—S1	125.29 (8)	C18—C16—S12	124.91 (9)
C8—C7—C11	108.14 (10)	C19—C18—C22	107.80 (9)
C8—C7—C5	123.58 (10)	C19—C18—C16	124.02 (10)
C11—C7—C5	128.26 (10)	C22—C18—C16	128.15 (10)
C8—C7—Fe1	69.72 (6)	C19—C18—Fe2	69.38 (6)
C11—C7—Fe1	69.79 (6)	C22—C18—Fe2	69.52 (6)
C5—C7—Fe1	124.74 (8)	C16—C18—Fe2	124.96 (8)
C9—C8—C7	107.53 (10)	C20—C19—C18	107.94 (10)
C9—C8—Fe1	70.23 (7)	C20—C19—Fe2	70.20 (6)
C7—C8—Fe1	69.05 (6)	C18—C19—Fe2	69.36 (6)
C9—C8—H8	126.2	C20—C19—H19	126.0
C7—C8—H8	126.2	C18—C19—H19	126.0
Fe1—C8—H8	126.2	Fe2—C19—H19	126.0
C8—C9—C10	108.53 (10)	C19—C20—C21	108.20 (10)
C8—C9—Fe1	69.16 (6)	C19—C20—Fe2	69.13 (6)
C10—C9—Fe1	69.64 (7)	C21—C20—Fe2	69.67 (6)
C8—C9—H9	125.7	C19—C20—H20	125.9
C10—C9—H9	125.7	C21—C20—H20	125.9
Fe1—C9—H9	125.7	Fe2—C20—H20	125.9
C9—C10—C11	108.34 (10)	C22—C21—C20	108.53 (10)
C9—C10—Fe1	69.80 (7)	C22—C21—Fe2	69.26 (6)
C11—C10—Fe1	69.28 (6)	C20—C21—Fe2	69.68 (6)
C9—C10—H10	125.8	C22—C21—H21	125.7

C11—C10—H10	125.8	C20—C21—H21	125.7
Fe1—C10—H10	125.8	Fe2—C21—H21	125.7
C10—C11—C7	107.45 (10)	C21—C22—C18	107.52 (10)
C10—C11—Fe1	70.03 (7)	C21—C22—Fe2	70.01 (6)
C7—C11—Fe1	68.95 (6)	C18—C22—Fe2	69.20 (6)
C10—C11—H11	126.3	C21—C22—H22	126.2
C7—C11—H11	126.3	C18—C22—H22	126.2
Fe1—C11—H11	126.3	Fe2—C22—H22	126.2
C5'—C1'—C2'	108.25 (11)	C27—C23—C24	108.03 (11)
C5'—C1'—Fe1	69.74 (7)	C27—C23—Fe2	69.22 (7)
C2'—C1'—Fe1	69.82 (7)	C24—C23—Fe2	69.85 (7)
C5'—C1'—H1'	125.9	C27—C23—H23	126.0
C2'—C1'—H1'	125.9	C24—C23—H23	126.0
Fe1—C1'—H1'	125.9	Fe2—C23—H23	126.0
C3'—C2'—C1'	107.85 (11)	C25—C24—C23	107.61 (11)
C3'—C2'—Fe1	69.83 (7)	C25—C24—Fe2	69.93 (6)
C1'—C2'—Fe1	69.52 (6)	C23—C24—Fe2	69.42 (7)
C3'—C2'—H2'	126.1	C25—C24—H24	126.2
C1'—C2'—H2'	126.1	C23—C24—H24	126.2
Fe1—C2'—H2'	126.1	Fe2—C24—H24	126.2
C2'—C3'—C4'	107.92 (12)	C26—C25—C24	108.14 (11)
C2'—C3'—Fe1	69.71 (7)	C26—C25—Fe2	69.03 (6)
C4'—C3'—Fe1	69.42 (7)	C24—C25—Fe2	69.61 (6)
C2'—C3'—H3'	126.0	C26—C25—H25	125.9
C4'—C3'—H3'	126.0	C24—C25—H25	125.9
Fe1—C3'—H3'	126.0	Fe2—C25—H25	125.9
C5'—C4'—C3'	108.39 (11)	C27—C26—C25	108.12 (11)
C5'—C4'—Fe1	69.84 (7)	C27—C26—Fe2	69.54 (7)
C3'—C4'—Fe1	70.00 (7)	C25—C26—Fe2	70.37 (6)
C5'—C4'—H4'	125.8	C27—C26—H26	125.9
C3'—C4'—H4'	125.8	C25—C26—H26	125.9
Fe1—C4'—H4'	125.8	Fe2—C26—H26	125.9
C4'—C5'—C1'	107.59 (12)	C26—C27—C23	108.10 (11)
C4'—C5'—Fe1	69.57 (7)	C26—C27—Fe2	69.81 (7)
C1'—C5'—Fe1	69.64 (7)	C23—C27—Fe2	70.07 (7)
C4'—C5'—H5'	126.2	C26—C27—H27	126.0
C1'—C5'—H5'	126.2	C23—C27—H27	126.0
Fe1—C5'—H5'	126.2	Fe2—C27—H27	126.0
C5—S1—N2—C3	0.37 (9)	C16—S12—N13—C14	0.22 (9)
S1—N2—C3—N4	-0.08 (14)	S12—N13—C14—N15	0.12 (14)
S1—N2—C3—Cl6	-179.70 (6)	S12—N13—C14—Cl17	-179.59 (6)
N2—C3—N4—C5	-0.38 (15)	N13—C14—N15—C16	-0.50 (15)
Cl6—C3—N4—C5	179.25 (8)	Cl17—C14—N15—C16	179.21 (8)
C3—N4—C5—C7	177.97 (10)	C14—N15—C16—C18	179.28 (10)
C3—N4—C5—S1	0.63 (11)	C14—N15—C16—S12	0.62 (11)

N2—S1—C5—N4	-0.62 (9)	N13—S12—C16—N15	-0.52 (9)
N2—S1—C5—C7	-177.90 (10)	N13—S12—C16—C18	-179.16 (10)
N4—C5—C7—C8	-9.96 (17)	N15—C16—C18—C19	-1.36 (17)
S1—C5—C7—C8	167.01 (9)	S12—C16—C18—C19	177.13 (8)
N4—C5—C7—C11	168.06 (11)	N15—C16—C18—C22	176.34 (10)
S1—C5—C7—C11	-14.97 (16)	S12—C16—C18—C22	-5.18 (16)
N4—C5—C7—Fe1	77.41 (13)	N15—C16—C18—Fe2	85.98 (13)
S1—C5—C7—Fe1	-105.62 (10)	S12—C16—C18—Fe2	-95.54 (11)
C11—C7—C8—C9	0.53 (12)	C22—C18—C19—C20	0.73 (12)
C5—C7—C8—C9	178.90 (10)	C16—C18—C19—C20	178.82 (10)
Fe1—C7—C8—C9	59.96 (8)	Fe2—C18—C19—C20	59.83 (8)
C11—C7—C8—Fe1	-59.43 (8)	C22—C18—C19—Fe2	-59.11 (7)
C5—C7—C8—Fe1	118.94 (10)	C16—C18—C19—Fe2	118.99 (10)
C7—C8—C9—C10	-0.55 (13)	C18—C19—C20—C21	-0.42 (13)
Fe1—C8—C9—C10	58.65 (8)	Fe2—C19—C20—C21	58.88 (8)
C7—C8—C9—Fe1	-59.21 (8)	C18—C19—C20—Fe2	-59.31 (7)
C8—C9—C10—C11	0.37 (13)	C19—C20—C21—C22	-0.04 (13)
Fe1—C9—C10—C11	58.73 (8)	Fe2—C20—C21—C22	58.51 (8)
C8—C9—C10—Fe1	-58.36 (8)	C19—C20—C21—Fe2	-58.55 (8)
C9—C10—C11—C7	-0.04 (13)	C20—C21—C22—C18	0.49 (12)
Fe1—C10—C11—C7	59.01 (8)	Fe2—C21—C22—C18	59.26 (7)
C9—C10—C11—Fe1	-59.05 (8)	C20—C21—C22—Fe2	-58.77 (8)
C8—C7—C11—C10	-0.30 (12)	C19—C18—C22—C21	-0.75 (12)
C5—C7—C11—C10	-178.57 (11)	C16—C18—C22—C21	-178.74 (10)
Fe1—C7—C11—C10	-59.69 (8)	Fe2—C18—C22—C21	-59.77 (8)
C8—C7—C11—Fe1	59.39 (8)	C19—C18—C22—Fe2	59.02 (7)
C5—C7—C11—Fe1	-118.88 (11)	C16—C18—C22—Fe2	-118.97 (11)
C5'—C1'—C2'—C3'	0.18 (13)	C27—C23—C24—C25	0.87 (13)
Fe1—C1'—C2'—C3'	59.54 (8)	Fe2—C23—C24—C25	59.75 (8)
C5'—C1'—C2'—Fe1	-59.36 (8)	C27—C23—C24—Fe2	-58.87 (8)
C1'—C2'—C3'—C4'	-0.25 (13)	C23—C24—C25—C26	-1.00 (13)
Fe1—C2'—C3'—C4'	59.10 (8)	Fe2—C24—C25—C26	58.43 (8)
C1'—C2'—C3'—Fe1	-59.35 (8)	C23—C24—C25—Fe2	-59.43 (8)
C2'—C3'—C4'—C5'	0.22 (14)	C24—C25—C26—C27	0.74 (13)
Fe1—C3'—C4'—C5'	59.50 (9)	Fe2—C25—C26—C27	59.53 (8)
C2'—C3'—C4'—Fe1	-59.28 (8)	C24—C25—C26—Fe2	-58.79 (8)
C3'—C4'—C5'—C1'	-0.11 (14)	C25—C26—C27—C23	-0.20 (13)
Fe1—C4'—C5'—C1'	59.50 (8)	Fe2—C26—C27—C23	59.85 (8)
C3'—C4'—C5'—Fe1	-59.61 (9)	C25—C26—C27—Fe2	-60.05 (8)
C2'—C1'—C5'—C4'	-0.05 (13)	C24—C23—C27—C26	-0.42 (14)
Fe1—C1'—C5'—C4'	-59.45 (8)	Fe2—C23—C27—C26	-59.69 (8)
C2'—C1'—C5'—Fe1	59.40 (8)	C24—C23—C27—Fe2	59.27 (8)

Table 5. Geometric parameters of **5** (bond lengths in Å and bond angles in °) MGC

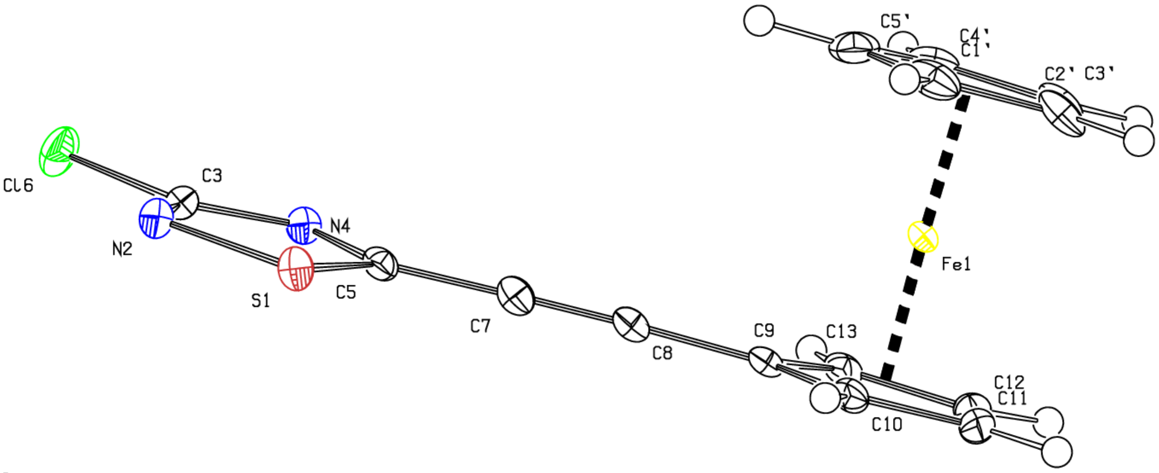

Fe1—C13	2.0359 (15)	C2'—C3'	1.427 (2)
Fe1—C9	2.0362 (14)	C2'—H2'	1.0000
Fe1—C10	2.0405 (15)	C3'—C4'	1.418 (2)
Fe1—C3'	2.0407 (15)	C3'—H3'	1.0000
Fe1—C4'	2.0423 (15)	C4'—C5'	1.427 (2)
Fe1—C5'	2.0446 (16)	C4'—H4'	1.0000
Fe1—C1'	2.0460 (16)	C5—C7	1.414 (2)
Fe1—C12	2.0462 (15)	C5'—H5'	1.0000
Fe1—C2'	2.0473 (15)	C7—C8	1.204 (2)
Fe1—C11	2.0518 (15)	C8—C9	1.419 (2)
Cl6—C3	1.7186 (15)	C9—C13	1.442 (2)
S1—N2	1.6563 (13)	C9—C10	1.443 (2)
S1—C5	1.7278 (15)	C10—C11	1.423 (2)
N2—C3	1.3032 (19)	C10—H10	1.0000
N4—C5	1.3202 (19)	C11—C12	1.422 (2)
N4—C3	1.3632 (18)	C11—H11	1.0000
C1'—C2'	1.423 (2)	C12—C13	1.418 (2)
C1'—C5'	1.425 (2)	C12—H12	1.0000
C1'—H1'	1.0000	C13—H13	1.0000
C13—Fe1—C9	41.48 (6)	C3'—C2'—H2'	126.0
C13—Fe1—C10	69.84 (6)	Fe1—C2'—H2'	126.0
C9—Fe1—C10	41.47 (6)	N2—C3—N4	122.30 (13)
C13—Fe1—C3'	121.25 (6)	N2—C3—Cl6	120.00 (11)
C9—Fe1—C3'	159.22 (6)	N4—C3—Cl6	117.70 (11)
C10—Fe1—C3'	156.92 (6)	C4'—C3'—C2'	108.29 (14)
C13—Fe1—C4'	105.81 (6)	C4'—C3'—Fe1	69.73 (8)
C9—Fe1—C4'	123.67 (6)	C2'—C3'—Fe1	69.82 (8)
C10—Fe1—C4'	161.59 (6)	C4'—C3'—H3'	125.9
C3'—Fe1—C4'	40.66 (6)	C2'—C3'—H3'	125.9
C13—Fe1—C5'	122.10 (7)	Fe1—C3'—H3'	125.9
C9—Fe1—C5'	108.64 (6)	C3'—C4'—C5'	107.77 (14)

C10—Fe1—C5'	125.33 (6)	C3'—C4'—Fe1	69.61 (8)
C3'—Fe1—C5'	68.47 (7)	C5'—C4'—Fe1	69.66 (9)
C4'—Fe1—C5'	40.86 (6)	C3'—C4'—H4'	126.1
C13—Fe1—C1'	159.05 (6)	C5'—C4'—H4'	126.1
C9—Fe1—C1'	123.52 (6)	Fe1—C4'—H4'	126.1
C10—Fe1—C1'	108.54 (6)	N4—C5—C7	124.99 (13)
C3'—Fe1—C1'	68.65 (6)	N4—C5—S1	111.77 (11)
C4'—Fe1—C1'	68.80 (7)	C7—C5—S1	123.23 (11)
C5'—Fe1—C1'	40.77 (7)	C1'—C5'—C4'	108.20 (14)
C13—Fe1—C12	40.64 (6)	C1'—C5'—Fe1	69.67 (9)
C9—Fe1—C12	68.75 (6)	C4'—C5'—Fe1	69.48 (9)
C10—Fe1—C12	68.87 (6)	C1'—C5'—H5'	125.9
C3'—Fe1—C12	105.43 (6)	C4'—C5'—H5'	125.9
C4'—Fe1—C12	120.15 (6)	Fe1—C5'—H5'	125.9
C5'—Fe1—C12	156.94 (7)	C8—C7—C5	178.02 (16)
C1'—Fe1—C12	159.72 (7)	C7—C8—C9	178.28 (15)
C13—Fe1—C2'	158.04 (6)	C8—C9—C13	126.16 (13)
C9—Fe1—C2'	159.02 (6)	C8—C9—C10	125.88 (13)
C10—Fe1—C2'	122.02 (6)	C13—C9—C10	107.94 (12)
C3'—Fe1—C2'	40.86 (6)	C8—C9—Fe1	125.63 (10)
C4'—Fe1—C2'	68.65 (7)	C13—C9—Fe1	69.25 (8)
C5'—Fe1—C2'	68.46 (7)	C10—C9—Fe1	69.43 (8)
C1'—Fe1—C2'	40.70 (7)	C11—C10—C9	107.18 (13)
C12—Fe1—C2'	122.34 (7)	C11—C10—Fe1	70.08 (8)
C13—Fe1—C11	68.75 (6)	C9—C10—Fe1	69.10 (8)
C9—Fe1—C11	68.70 (6)	C11—C10—H10	126.4
C10—Fe1—C11	40.68 (6)	C9—C10—H10	126.4
C3'—Fe1—C11	120.66 (6)	Fe1—C10—H10	126.4
C4'—Fe1—C11	155.90 (6)	C12—C11—C10	108.69 (13)
C5'—Fe1—C11	161.67 (7)	C12—C11—Fe1	69.49 (8)
C1'—Fe1—C11	124.44 (7)	C10—C11—Fe1	69.23 (8)
C12—Fe1—C11	40.60 (6)	C12—C11—H11	125.6
C2'—Fe1—C11	107.15 (7)	C10—C11—H11	125.6
N2—S1—C5	92.72 (7)	Fe1—C11—H11	125.6
C3—N2—S1	106.27 (10)	C13—C12—C11	108.77 (13)
C5—N4—C3	106.93 (12)	C13—C12—Fe1	69.29 (8)
C2'—C1'—C5'	107.83 (14)	C11—C12—Fe1	69.92 (9)
C2'—C1'—Fe1	69.70 (9)	C13—C12—H12	125.6
C5'—C1'—Fe1	69.56 (9)	C11—C12—H12	125.6
C2'—C1'—H1'	126.1	Fe1—C12—H12	125.6
C5'—C1'—H1'	126.1	C12—C13—C9	107.40 (13)
Fe1—C1'—H1'	126.1	C12—C13—Fe1	70.07 (8)
C1'—C2'—C3'	107.91 (14)	C9—C13—Fe1	69.27 (8)
C1'—C2'—Fe1	69.60 (8)	C12—C13—H13	126.3
C3'—C2'—Fe1	69.32 (8)	C9—C13—H13	126.3
C1'—C2'—H2'	126.0	Fe1—C13—H13	126.3

C5—S1—N2—C3	0.44 (11)	Fe1—C4'—C5'—C1'	59.10 (10)
C5'—C1'—C2'—C3'	-0.40 (17)	C3'—C4'—C5'—Fe1	-59.40 (10)
Fe1—C1'—C2'—C3'	58.93 (10)	C8—C9—C10—C11	-179.79 (13)
C5'—C1'—C2'—Fe1	-59.33 (10)	C13—C9—C10—C11	-1.30 (15)
S1—N2—C3—N4	-0.50 (17)	Fe1—C9—C10—C11	-60.02 (10)
S1—N2—C3—Cl6	179.65 (8)	C8—C9—C10—Fe1	-119.77 (14)
C5—N4—C3—N2	0.25 (18)	C13—C9—C10—Fe1	58.71 (9)
C5—N4—C3—Cl6	-179.89 (10)	C9—C10—C11—C12	0.98 (16)
C1'—C2'—C3'—C4'	0.22 (17)	Fe1—C10—C11—C12	-58.41 (10)
Fe1—C2'—C3'—C4'	59.32 (10)	C9—C10—C11—Fe1	59.39 (10)
C1'—C2'—C3'—Fe1	-59.11 (10)	C10—C11—C12—C13	-0.29 (16)
C2'—C3'—C4'—C5'	0.05 (16)	Fe1—C11—C12—C13	-58.54 (10)
Fe1—C3'—C4'—C5'	59.43 (10)	C10—C11—C12—Fe1	58.25 (10)
C2'—C3'—C4'—Fe1	-59.38 (10)	C11—C12—C13—C9	-0.53 (16)
C3—N4—C5—C7	-179.19 (13)	Fe1—C12—C13—C9	-59.45 (9)
C3—N4—C5—S1	0.13 (14)	C11—C12—C13—Fe1	58.92 (10)
N2—S1—C5—N4	-0.35 (11)	C8—C9—C13—C12	179.61 (13)
N2—S1—C5—C7	178.99 (12)	C10—C9—C13—C12	1.13 (15)
C2'—C1'—C5'—C4'	0.43 (17)	Fe1—C9—C13—C12	59.96 (10)
Fe1—C1'—C5'—C4'	-58.99 (10)	C8—C9—C13—Fe1	119.66 (14)
C2'—C1'—C5'—Fe1	59.42 (10)	C10—C9—C13—Fe1	-58.82 (10)
C3'—C4'—C5'—C1'	-0.30 (16)		

Table 6. Geometric parameters of **6** (bond lengths in Å and bond angles in °) MGI

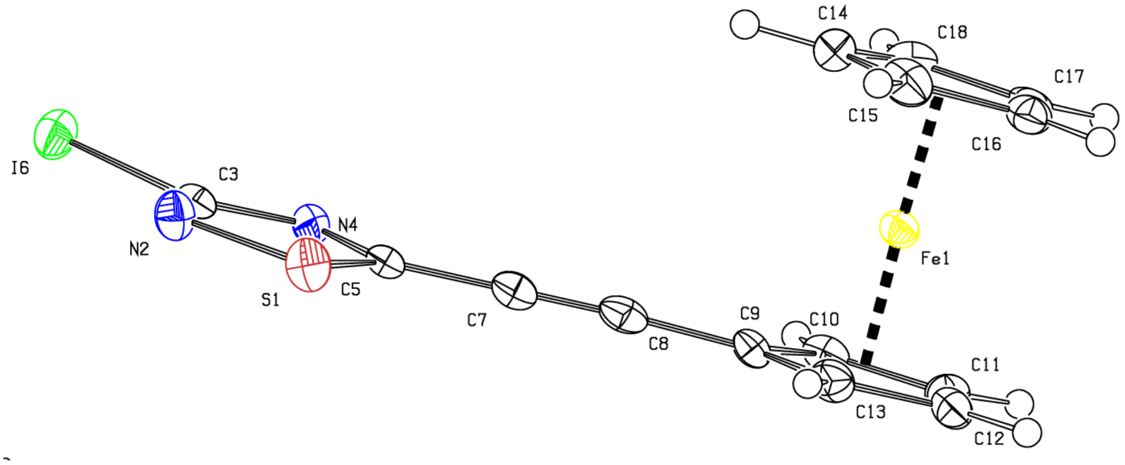

I6—C3	2.083 (5)	C9—C13	1.436 (6)
Fe1—C10	2.032 (5)	C9—C10	1.446 (6)
Fe1—C9	2.037 (4)	C10—C11	1.410 (7)
Fe1—C13	2.038 (5)	C10—H10	1.0000
Fe1—C17	2.040 (4)	C11—C12	1.428 (7)
Fe1—C16	2.042 (5)	C11—H11	1.0000
Fe1—C14	2.049 (5)	C12—C13	1.417 (7)
Fe1—C15	2.053 (5)	C12—H12	1.0000

Fe1—C12	2.054 (5)	C13—H13	1.0000
Fe1—C11	2.060 (5)	C14—C15	1.416 (7)
Fe1—C18	2.060 (5)	C14—C18	1.429 (7)
S1—N2	1.664 (4)	C14—H14	1.0000
S1—C5	1.723 (5)	C15—C16	1.423 (7)
N2—C3	1.306 (6)	C15—H15	1.0000
N4—C5	1.329 (6)	C16—C17	1.425 (7)
N4—C3	1.366 (6)	C16—H16	1.0000
C5—C7	1.422 (6)	C17—C18	1.423 (7)
C7—C8	1.199 (6)	C17—H17	1.0000
C8—C9	1.423 (7)	C18—H18	1.0000
C10—Fe1—C9	41.64 (18)	C13—C9—C10	107.3 (4)
C10—Fe1—C13	69.56 (19)	C8—C9—Fe1	125.8 (3)
C9—Fe1—C13	41.26 (18)	C13—C9—Fe1	69.4 (3)
C10—Fe1—C17	122.16 (19)	C10—C9—Fe1	69.0 (2)
C9—Fe1—C17	160.55 (19)	C11—C10—C9	107.7 (4)
C13—Fe1—C17	155.80 (19)	C11—C10—Fe1	70.9 (3)
C10—Fe1—C16	158.51 (19)	C9—C10—Fe1	69.3 (3)
C9—Fe1—C16	157.87 (19)	C11—C10—H10	126.1
C13—Fe1—C16	121.01 (19)	C9—C10—H10	126.1
C17—Fe1—C16	40.85 (18)	Fe1—C10—H10	126.1
C10—Fe1—C14	123.1 (2)	C10—C11—C12	108.7 (4)
C9—Fe1—C14	109.37 (19)	C10—C11—Fe1	68.8 (3)
C13—Fe1—C14	125.7 (2)	C12—C11—Fe1	69.5 (3)
C17—Fe1—C14	68.35 (19)	C10—C11—H11	125.6
C16—Fe1—C14	68.11 (19)	C12—C11—H11	125.6
C10—Fe1—C15	159.2 (2)	Fe1—C11—H11	125.6
C9—Fe1—C15	123.06 (19)	C13—C12—C11	108.2 (4)
C13—Fe1—C15	108.2 (2)	C13—C12—Fe1	69.1 (3)
C17—Fe1—C15	68.60 (19)	C11—C12—Fe1	69.9 (3)
C16—Fe1—C15	40.66 (19)	C13—C12—H12	125.9
C14—Fe1—C15	40.37 (19)	C11—C12—H12	125.9
C10—Fe1—C12	68.68 (19)	Fe1—C12—H12	125.9
C9—Fe1—C12	68.68 (18)	C12—C13—C9	108.0 (4)
C13—Fe1—C12	40.50 (18)	C12—C13—Fe1	70.4 (3)
C17—Fe1—C12	119.94 (19)	C9—C13—Fe1	69.3 (3)
C16—Fe1—C12	106.26 (19)	C12—C13—H13	126.0
C14—Fe1—C12	161.2 (2)	C9—C13—H13	126.0
C15—Fe1—C12	123.8 (2)	Fe1—C13—H13	126.0
C10—Fe1—C11	40.30 (19)	C15—C14—C18	108.7 (4)
C9—Fe1—C11	68.55 (19)	C15—C14—Fe1	70.0 (3)
C13—Fe1—C11	68.4 (2)	C18—C14—Fe1	70.1 (3)
C17—Fe1—C11	105.82 (19)	C15—C14—H14	125.6
C16—Fe1—C11	122.49 (19)	C18—C14—H14	125.6
C14—Fe1—C11	157.6 (2)	Fe1—C14—H14	125.6
C15—Fe1—C11	159.6 (2)	C14—C15—C16	107.6 (4)

C12—Fe1—C11	40.61 (19)	C14—C15—Fe1	69.7 (3)
C10—Fe1—C18	107.2 (2)	C16—C15—Fe1	69.2 (3)
C9—Fe1—C18	125.1 (2)	C14—C15—H15	126.2
C13—Fe1—C18	162.5 (2)	C16—C15—H15	126.2
C17—Fe1—C18	40.61 (19)	Fe1—C15—H15	126.2
C16—Fe1—C18	68.38 (19)	C15—C16—C17	108.2 (4)
C14—Fe1—C18	40.68 (19)	C15—C16—Fe1	70.1 (3)
C15—Fe1—C18	68.4 (2)	C17—C16—Fe1	69.5 (3)
C12—Fe1—C18	155.8 (2)	C15—C16—H16	125.9
C11—Fe1—C18	120.9 (2)	C17—C16—H16	125.9
N2—S1—C5	93.0 (2)	Fe1—C16—H16	125.9
C3—N2—S1	106.0 (3)	C18—C17—C16	108.1 (4)
C5—N4—C3	107.0 (4)	C18—C17—Fe1	70.4 (3)
N2—C3—N4	122.3 (4)	C16—C17—Fe1	69.6 (3)
N2—C3—I6	119.6 (3)	C18—C17—H17	126.0
N4—C3—I6	118.0 (3)	C16—C17—H17	126.0
N4—C5—C7	124.4 (4)	Fe1—C17—H17	126.0
N4—C5—S1	111.6 (3)	C17—C18—C14	107.3 (4)
C7—C5—S1	123.9 (4)	C17—C18—Fe1	69.0 (3)
C8—C7—C5	177.5 (5)	C14—C18—Fe1	69.3 (3)
C7—C8—C9	177.6 (5)	C17—C18—H18	126.3
C8—C9—C13	126.2 (4)	C14—C18—H18	126.3
C8—C9—C10	126.5 (4)	Fe1—C18—H18	126.3
C5—S1—N2—C3	0.3 (3)	C11—C12—C13—Fe1	-59.2 (3)
S1—N2—C3—N4	-0.3 (5)	C8—C9—C13—C12	-179.9 (4)
S1—N2—C3—I6	-178.1 (2)	C10—C9—C13—C12	-1.2 (5)
C5—N4—C3—N2	0.1 (6)	Fe1—C9—C13—C12	-60.0 (3)
C5—N4—C3—I6	177.9 (3)	C8—C9—C13—Fe1	-119.9 (5)
C3—N4—C5—C7	-177.5 (4)	C10—C9—C13—Fe1	58.8 (3)
C3—N4—C5—S1	0.1 (5)	C18—C14—C15—C16	-0.5 (5)
N2—S1—C5—N4	-0.2 (4)	Fe1—C14—C15—C16	59.0 (3)
N2—S1—C5—C7	177.4 (4)	C18—C14—C15—Fe1	-59.6 (3)
C8—C9—C10—C11	-179.5 (4)	C14—C15—C16—C17	0.0 (5)
C13—C9—C10—C11	1.8 (5)	Fe1—C15—C16—C17	59.3 (3)
Fe1—C9—C10—C11	60.8 (3)	C14—C15—C16—Fe1	-59.3 (3)
C8—C9—C10—Fe1	119.7 (5)	C15—C16—C17—C18	0.6 (5)
C13—C9—C10—Fe1	-59.1 (3)	Fe1—C16—C17—C18	60.2 (3)
C9—C10—C11—C12	-1.7 (5)	C15—C16—C17—Fe1	-59.6 (3)
Fe1—C10—C11—C12	58.1 (3)	C16—C17—C18—C14	-0.9 (5)
C9—C10—C11—Fe1	-59.8 (3)	Fe1—C17—C18—C14	58.8 (3)
C10—C11—C12—C13	1.0 (5)	C16—C17—C18—Fe1	-59.7 (3)
Fe1—C11—C12—C13	58.7 (3)	C15—C14—C18—C17	0.9 (5)
C10—C11—C12—Fe1	-57.7 (3)	Fe1—C14—C18—C17	-58.6 (3)
C11—C12—C13—C9	0.2 (5)	C15—C14—C18—Fe1	59.5 (3)
Fe1—C12—C13—C9	59.3 (3)		

Table 7. Geometric parameters of **8** (bond lengths in Å and bond angles in °) MGD

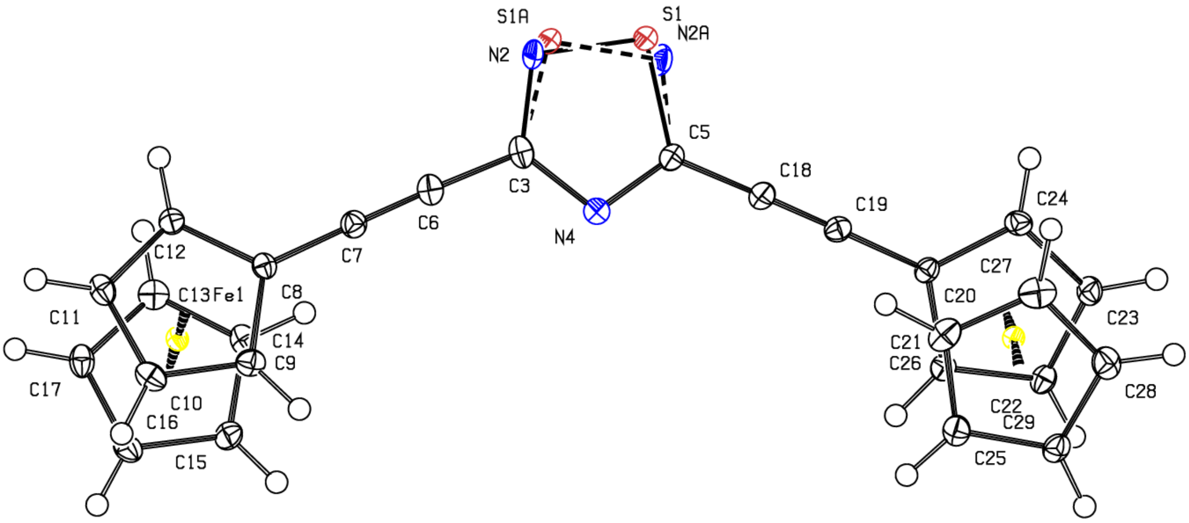

Fe1—C9	2.0400 (12)	C10—H10	0.9500
Fe1—C8	2.0413 (12)	C11—C12	1.4224 (17)
Fe1—C15	2.0423 (12)	C11—H11	0.9500
Fe1—C12	2.0428 (12)	C12—H12	0.9500
Fe1—C14	2.0437 (12)	C13—C14	1.4224 (19)
Fe1—C11	2.0498 (12)	C13—C17	1.4255 (18)
Fe1—C10	2.0498 (12)	C13—H13	0.9500
Fe1—C16	2.0509 (12)	C14—C15	1.4262 (18)
Fe1—C17	2.0520 (12)	C14—H14	0.9500
Fe1—C13	2.0524 (12)	C15—C16	1.4298 (18)
Fe2—C20	2.0249 (12)	C15—H15	0.9500
Fe2—C21	2.0281 (12)	C16—C17	1.4227 (19)
Fe2—C25	2.0377 (12)	C16—H16	0.9500
Fe2—C26	2.0439 (12)	C17—H17	0.9500
Fe2—C29	2.0442 (12)	C18—C19	1.2032 (17)
Fe2—C24	2.0458 (12)	C19—C20	1.4220 (16)
Fe2—C27	2.0463 (13)	C20—C21	1.4406 (17)
Fe2—C28	2.0496 (13)	C20—C24	1.4411 (17)
Fe2—C22	2.0525 (12)	C21—C22	1.4240 (17)
Fe2—C23	2.0597 (12)	C21—H21	0.9500
S1—N2	1.653 (6)	C22—C23	1.4264 (18)
S1—C5	1.7059 (16)	C22—H22	0.9500
N2—C3	1.381 (7)	C23—C24	1.4233 (17)
N4—C5	1.3368 (16)	C23—H23	0.9500
N4—C3	1.3589 (17)	C24—H24	0.9500
C3—C6	1.4237 (17)	C25—C26	1.4226 (19)
C3—S1A	1.609 (5)	C25—C29	1.4268 (17)
C5—N2A	1.374 (9)	C25—H25	0.9500
C5—C18	1.4176 (17)	C26—C27	1.427 (2)
C6—C7	1.2075 (17)	C26—H26	0.9500

C7—C8	1.4219 (16)	C27—C28	1.426 (2)
C8—C9	1.4378 (17)	C27—H27	0.9500
C8—C12	1.4396 (17)	C28—C29	1.4247 (19)
C9—C10	1.4226 (18)	C28—H28	0.9500
C9—H9	0.9500	C29—H29	0.9500
C10—C11	1.4258 (19)	S1A—N2A	1.644 (10)
C9—Fe1—C8	41.26 (5)	C8—C9—H9	126.1
C9—Fe1—C15	106.07 (5)	Fe1—C9—H9	126.0
C8—Fe1—C15	122.53 (5)	C9—C10—C11	108.38 (11)
C9—Fe1—C12	69.38 (5)	C9—C10—Fe1	69.28 (7)
C8—Fe1—C12	41.28 (5)	C11—C10—Fe1	69.64 (7)
C15—Fe1—C12	159.85 (5)	C9—C10—H10	125.8
C9—Fe1—C14	121.59 (5)	C11—C10—H10	125.8
C8—Fe1—C14	107.05 (5)	Fe1—C10—H10	126.8
C15—Fe1—C14	40.86 (5)	C12—C11—C10	108.41 (11)
C12—Fe1—C14	123.65 (5)	C12—C11—Fe1	69.40 (7)
C9—Fe1—C11	68.78 (5)	C10—C11—Fe1	69.65 (7)
C8—Fe1—C11	68.80 (5)	C12—C11—H11	125.8
C15—Fe1—C11	157.65 (5)	C10—C11—H11	125.8
C12—Fe1—C11	40.68 (5)	Fe1—C11—H11	126.7
C14—Fe1—C11	160.35 (5)	C11—C12—C8	107.72 (11)
C9—Fe1—C10	40.71 (5)	C11—C12—Fe1	69.93 (7)
C8—Fe1—C10	68.79 (5)	C8—C12—Fe1	69.30 (7)
C15—Fe1—C10	121.37 (5)	C11—C12—H12	126.1
C12—Fe1—C10	68.74 (5)	C8—C12—H12	126.1
C14—Fe1—C10	157.47 (6)	Fe1—C12—H12	126.2
C11—Fe1—C10	40.71 (5)	C14—C13—C17	108.06 (11)
C9—Fe1—C16	122.33 (5)	C14—C13—Fe1	69.35 (7)
C8—Fe1—C16	159.15 (5)	C17—C13—Fe1	69.66 (7)
C15—Fe1—C16	40.89 (5)	C14—C13—H13	126.0
C12—Fe1—C16	158.06 (5)	C17—C13—H13	126.0
C14—Fe1—C16	68.57 (5)	Fe1—C13—H13	126.6
C11—Fe1—C16	122.19 (5)	C13—C14—C15	108.15 (11)
C10—Fe1—C16	107.02 (5)	C13—C14—Fe1	70.01 (7)
C9—Fe1—C17	159.06 (5)	C15—C14—Fe1	69.52 (7)
C8—Fe1—C17	158.65 (5)	C13—C14—H14	125.9
C15—Fe1—C17	68.64 (5)	C15—C14—H14	125.9
C12—Fe1—C17	122.53 (5)	Fe1—C14—H14	126.1
C14—Fe1—C17	68.49 (5)	C14—C15—C16	107.72 (11)
C11—Fe1—C17	107.98 (5)	C14—C15—Fe1	69.62 (7)
C10—Fe1—C17	123.43 (5)	C16—C15—Fe1	69.88 (7)
C16—Fe1—C17	40.58 (5)	C14—C15—H15	126.1
C9—Fe1—C13	158.16 (5)	C16—C15—H15	126.1
C8—Fe1—C13	122.47 (5)	Fe1—C15—H15	125.9
C15—Fe1—C13	68.57 (5)	C17—C16—C15	108.06 (11)
C12—Fe1—C13	107.96 (5)	C17—C16—Fe1	69.75 (7)

C14—Fe1—C13	40.64 (5)	C15—C16—Fe1	69.23 (7)
C11—Fe1—C13	124.18 (5)	C17—C16—H16	126.0
C10—Fe1—C13	160.20 (5)	C15—C16—H16	126.0
C16—Fe1—C13	68.34 (5)	Fe1—C16—H16	126.6
C17—Fe1—C13	40.65 (5)	C16—C17—C13	108.01 (11)
C20—Fe2—C21	41.64 (5)	C16—C17—Fe1	69.67 (7)
C20—Fe2—C25	119.71 (5)	C13—C17—Fe1	69.69 (7)
C21—Fe2—C25	104.09 (5)	C16—C17—H17	126.0
C20—Fe2—C26	106.44 (5)	C13—C17—H17	126.0
C21—Fe2—C26	121.71 (5)	Fe1—C17—H17	126.2
C25—Fe2—C26	40.80 (5)	C19—C18—C5	178.54 (14)
C20—Fe2—C29	155.46 (5)	C18—C19—C20	179.02 (13)
C21—Fe2—C29	118.99 (5)	C19—C20—C21	126.46 (11)
C25—Fe2—C29	40.92 (5)	C19—C20—C24	125.81 (11)
C26—Fe2—C29	68.69 (5)	C21—C20—C24	107.61 (10)
C20—Fe2—C24	41.46 (5)	C19—C20—Fe2	122.86 (8)
C21—Fe2—C24	69.61 (5)	C21—C20—Fe2	69.30 (7)
C25—Fe2—C24	157.52 (5)	C24—C20—Fe2	70.05 (7)
C26—Fe2—C24	123.08 (5)	C22—C21—C20	107.75 (10)
C29—Fe2—C24	161.03 (5)	C22—C21—Fe2	70.50 (7)
C20—Fe2—C27	124.34 (5)	C20—C21—Fe2	69.06 (7)
C21—Fe2—C27	159.91 (5)	C22—C21—H21	126.1
C25—Fe2—C27	68.73 (6)	C20—C21—H21	126.1
C26—Fe2—C27	40.85 (6)	Fe2—C21—H21	125.9
C29—Fe2—C27	68.62 (5)	C21—C22—C23	108.46 (11)
C24—Fe2—C27	109.48 (5)	C21—C22—Fe2	68.66 (7)
C20—Fe2—C28	161.90 (5)	C23—C22—Fe2	69.97 (7)
C21—Fe2—C28	155.93 (5)	C21—C22—H22	125.8
C25—Fe2—C28	68.72 (5)	C23—C22—H22	125.8
C26—Fe2—C28	68.65 (6)	Fe2—C22—H22	127.2
C29—Fe2—C28	40.73 (5)	C24—C23—C22	108.35 (10)
C24—Fe2—C28	125.52 (5)	C24—C23—Fe2	69.19 (6)
C27—Fe2—C28	40.74 (6)	C22—C23—Fe2	69.44 (7)
C20—Fe2—C22	69.14 (5)	C24—C23—H23	125.8
C21—Fe2—C22	40.84 (5)	C22—C23—H23	125.8
C25—Fe2—C22	121.40 (5)	Fe2—C23—H23	127.1
C26—Fe2—C22	158.12 (5)	C23—C24—C20	107.83 (10)
C29—Fe2—C22	105.98 (5)	C23—C24—Fe2	70.24 (7)
C24—Fe2—C22	68.63 (5)	C20—C24—Fe2	68.49 (6)
C27—Fe2—C22	158.87 (5)	C23—C24—H24	126.1
C28—Fe2—C22	121.99 (5)	C20—C24—H24	126.1
C20—Fe2—C23	69.04 (5)	Fe2—C24—H24	126.7
C21—Fe2—C23	68.91 (5)	C26—C25—C29	108.08 (11)
C25—Fe2—C23	158.92 (5)	C26—C25—Fe2	69.84 (7)
C26—Fe2—C23	159.66 (5)	C29—C25—Fe2	69.78 (7)
C29—Fe2—C23	123.74 (5)	C26—C25—H25	126.0

C24—Fe2—C23	40.57 (5)	C29—C25—H25	126.0
C27—Fe2—C23	124.28 (5)	Fe2—C25—H25	126.0
C28—Fe2—C23	109.16 (5)	C25—C26—C27	107.96 (11)
C22—Fe2—C23	40.59 (5)	C25—C26—Fe2	69.37 (7)
N2—S1—C5	94.1 (3)	C27—C26—Fe2	69.67 (7)
C3—N2—S1	105.7 (5)	C25—C26—H26	126.0
C5—N4—C3	108.01 (11)	C27—C26—H26	126.0
N4—C3—N2	119.7 (3)	Fe2—C26—H26	126.5
N4—C3—C6	120.68 (12)	C28—C27—C26	108.00 (12)
N2—C3—C6	119.6 (3)	C28—C27—Fe2	69.75 (7)
N4—C3—S1A	112.40 (19)	C26—C27—Fe2	69.49 (7)
C6—C3—S1A	126.9 (2)	C28—C27—H27	126.0
N4—C5—N2A	118.8 (4)	C26—C27—H27	126.0
N4—C5—C18	123.42 (11)	Fe2—C27—H27	126.3
N2A—C5—C18	117.8 (4)	C29—C28—C27	107.96 (12)
N4—C5—S1	112.43 (10)	C29—C28—Fe2	69.43 (7)
C18—C5—S1	124.14 (10)	C27—C28—Fe2	69.50 (7)
C7—C6—C3	177.62 (14)	C29—C28—H28	126.0
C6—C7—C8	178.93 (14)	C27—C28—H28	126.0
C7—C8—C9	125.37 (11)	Fe2—C28—H28	126.6
C7—C8—C12	126.89 (11)	C28—C29—C25	107.99 (11)
C9—C8—C12	107.71 (10)	C28—C29—Fe2	69.84 (7)
C7—C8—Fe1	125.35 (8)	C25—C29—Fe2	69.30 (7)
C9—C8—Fe1	69.33 (7)	C28—C29—H29	126.0
C12—C8—Fe1	69.41 (7)	C25—C29—H29	126.0
C10—C9—C8	107.78 (11)	Fe2—C29—H29	126.4
C10—C9—Fe1	70.01 (7)	C3—S1A—N2A	96.1 (4)
C8—C9—Fe1	69.42 (7)	C5—N2A—S1A	104.7 (7)
C10—C9—H9	126.1		
C5—S1—N2—C3	-0.3 (5)	C14—C13—C17—C16	-0.43 (14)
C5—N4—C3—N2	-1.0 (4)	Fe1—C13—C17—C16	-59.34 (9)
C5—N4—C3—C6	178.71 (11)	C14—C13—C17—Fe1	58.91 (8)
C5—N4—C3—S1A	-1.3 (3)	C19—C20—C21—C22	-176.38 (11)
S1—N2—C3—N4	0.8 (6)	C24—C20—C21—C22	-0.33 (13)
S1—N2—C3—C6	-178.9 (2)	Fe2—C20—C21—C22	-60.15 (8)
C3—N4—C5—N2A	1.7 (6)	C19—C20—C21—Fe2	-116.23 (12)
C3—N4—C5—C18	-177.80 (12)	C24—C20—C21—Fe2	59.82 (8)
C3—N4—C5—S1	0.74 (14)	C20—C21—C22—C23	0.45 (13)
N2—S1—C5—N4	-0.3 (3)	Fe2—C21—C22—C23	-58.80 (8)
N2—S1—C5—C18	178.2 (3)	C20—C21—C22—Fe2	59.24 (8)
C7—C8—C9—C10	-179.12 (11)	C21—C22—C23—C24	-0.40 (14)
C12—C8—C9—C10	-0.74 (13)	Fe2—C22—C23—C24	-58.39 (8)
Fe1—C8—C9—C10	-59.75 (8)	C21—C22—C23—Fe2	57.99 (8)
C7—C8—C9—Fe1	-119.37 (12)	C22—C23—C24—C20	0.19 (13)
C12—C8—C9—Fe1	59.00 (8)	Fe2—C23—C24—C20	-58.35 (8)
C8—C9—C10—C11	0.54 (14)	C22—C23—C24—Fe2	58.54 (8)

Fe1—C9—C10—C11	-58.83 (9)	C19—C20—C24—C23	176.17 (11)
C8—C9—C10—Fe1	59.37 (8)	C21—C20—C24—C23	0.08 (13)
C9—C10—C11—C12	-0.13 (14)	Fe2—C20—C24—C23	59.44 (8)
Fe1—C10—C11—C12	-58.73 (8)	C19—C20—C24—Fe2	116.73 (12)
C9—C10—C11—Fe1	58.60 (9)	C21—C20—C24—Fe2	-59.35 (8)
C10—C11—C12—C8	-0.33 (13)	C29—C25—C26—C27	0.35 (14)
Fe1—C11—C12—C8	-59.22 (8)	Fe2—C25—C26—C27	-59.17 (9)
C10—C11—C12—Fe1	58.89 (8)	C29—C25—C26—Fe2	59.53 (8)
C7—C8—C12—C11	179.01 (11)	C25—C26—C27—C28	-0.34 (14)
C9—C8—C12—C11	0.66 (13)	Fe2—C26—C27—C28	-59.33 (9)
Fe1—C8—C12—C11	59.61 (8)	C25—C26—C27—Fe2	58.99 (9)
C7—C8—C12—Fe1	119.39 (12)	C26—C27—C28—C29	0.20 (14)
C9—C8—C12—Fe1	-58.95 (8)	Fe2—C27—C28—C29	-58.97 (9)
C17—C13—C14—C15	0.16 (14)	C26—C27—C28—Fe2	59.16 (9)
Fe1—C13—C14—C15	59.26 (9)	C27—C28—C29—C25	0.02 (14)
C17—C13—C14—Fe1	-59.10 (8)	Fe2—C28—C29—C25	-58.99 (8)
C13—C14—C15—C16	0.17 (14)	C27—C28—C29—Fe2	59.01 (9)
Fe1—C14—C15—C16	59.74 (9)	C26—C25—C29—C28	-0.23 (14)
C13—C14—C15—Fe1	-59.57 (8)	Fe2—C25—C29—C28	59.33 (9)
C14—C15—C16—C17	-0.44 (14)	C26—C25—C29—Fe2	-59.56 (8)
Fe1—C15—C16—C17	59.14 (9)	N4—C3—S1A—N2A	0.5 (5)
C14—C15—C16—Fe1	-59.58 (9)	C6—C3—S1A—N2A	-179.5 (4)
C15—C16—C17—C13	0.54 (14)	N4—C5—N2A—S1A	-1.3 (9)
Fe1—C16—C17—C13	59.36 (8)	C18—C5—N2A—S1A	178.2 (4)
C15—C16—C17—Fe1	-58.82 (9)	C3—S1A—N2A—C5	0.4 (7)

Table 8. Geometric parameters of **10** (bond lengths in Å and bond angles in °) MPF

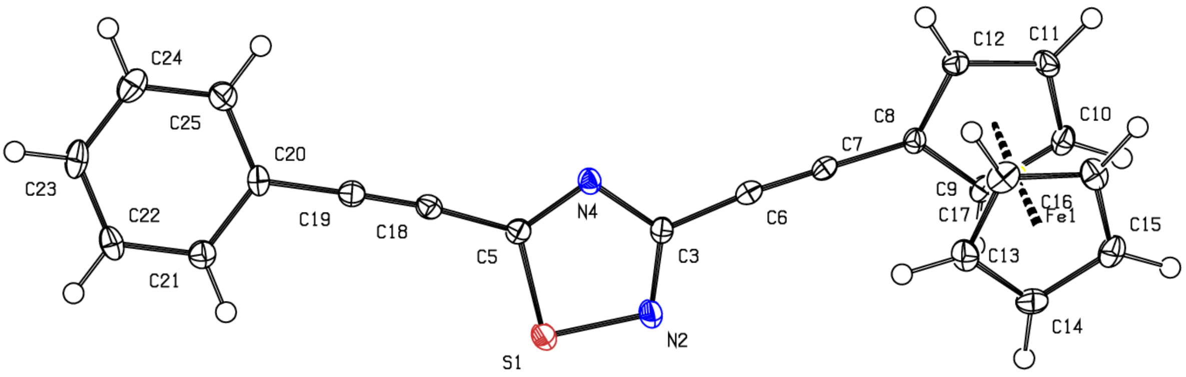

Fe1—C9	2.040 (3)	C11—H11	1.0000
Fe1—C15	2.043 (3)	C12—H12	1.0000
Fe1—C8	2.046 (2)	C13—C14	1.420 (4)
Fe1—C16	2.046 (2)	C13—C17	1.422 (4)
Fe1—C12	2.049 (3)	C13—H13	1.0000
Fe1—C17	2.054 (3)	C14—C15	1.424 (4)
Fe1—C10	2.057 (3)	C14—H14	1.0000

Fe1—C14	2.059 (3)	C15—C16	1.434 (4)
Fe1—C11	2.059 (2)	C15—H15	1.0000
Fe1—C13	2.059 (3)	C16—C17	1.421 (4)
S1—N2	1.649 (2)	C16—H16	1.0000
S1—C5	1.727 (3)	C17—H17	1.0000
N2—C3	1.329 (3)	C18—C19	1.195 (4)
N4—C5	1.321 (3)	C19—C20	1.434 (4)
N4—C3	1.376 (3)	C20—C25	1.397 (4)
C3—C6	1.431 (4)	C20—C21	1.402 (4)
C5—C18	1.424 (4)	C21—C22	1.385 (4)
C6—C7	1.202 (4)	C21—H21	0.9500
C7—C8	1.428 (4)	C22—C23	1.389 (4)
C8—C9	1.440 (4)	C22—H22	0.9500
C8—C12	1.441 (3)	C23—C24	1.389 (4)
C9—C10	1.424 (4)	C23—H23	0.9500
C9—H9	1.0000	C24—C25	1.387 (4)
C10—C11	1.422 (4)	C24—H24	0.9500
C10—H10	1.0000	C25—H25	0.9500
C11—C12	1.421 (4)		
C9—Fe1—C15	122.22 (11)	C11—C10—C9	108.4 (2)
C9—Fe1—C8	41.28 (10)	C11—C10—Fe1	69.86 (14)
C15—Fe1—C8	159.70 (10)	C9—C10—Fe1	69.05 (14)
C9—Fe1—C16	158.10 (10)	C11—C10—H10	125.8
C15—Fe1—C16	41.06 (10)	C9—C10—H10	125.8
C8—Fe1—C16	158.43 (10)	Fe1—C10—H10	125.8
C9—Fe1—C12	69.21 (10)	C12—C11—C10	108.5 (2)
C15—Fe1—C12	157.04 (11)	C12—C11—Fe1	69.36 (14)
C8—Fe1—C12	41.22 (10)	C10—C11—Fe1	69.71 (14)
C16—Fe1—C12	121.26 (11)	C12—C11—H11	125.7
C9—Fe1—C17	160.04 (11)	C10—C11—H11	125.7
C15—Fe1—C17	68.39 (11)	Fe1—C11—H11	125.7
C8—Fe1—C17	123.53 (11)	C11—C12—C8	107.9 (2)
C16—Fe1—C17	40.56 (11)	C11—C12—Fe1	70.14 (14)
C12—Fe1—C17	107.79 (11)	C8—C12—Fe1	69.29 (14)
C9—Fe1—C10	40.66 (10)	C11—C12—H12	126.1
C15—Fe1—C10	106.29 (11)	C8—C12—H12	126.1
C8—Fe1—C10	68.70 (10)	Fe1—C12—H12	126.1
C16—Fe1—C10	121.61 (10)	C14—C13—C17	108.3 (2)
C12—Fe1—C10	68.40 (10)	C14—C13—Fe1	69.81 (15)
C17—Fe1—C10	158.19 (11)	C17—C13—Fe1	69.58 (15)
C9—Fe1—C14	107.89 (11)	C14—C13—H13	125.9
C15—Fe1—C14	40.62 (10)	C17—C13—H13	125.9
C8—Fe1—C14	124.26 (10)	Fe1—C13—H13	125.9
C16—Fe1—C14	68.61 (11)	C13—C14—C15	107.9 (2)
C12—Fe1—C14	160.93 (10)	C13—C14—Fe1	69.84 (15)

C17—Fe1—C14	68.13 (11)	C15—C14—Fe1	69.11 (15)
C10—Fe1—C14	122.47 (11)	C13—C14—H14	126.1
C9—Fe1—C11	68.53 (11)	C15—C14—H14	126.1
C15—Fe1—C11	121.12 (11)	Fe1—C14—H14	126.1
C8—Fe1—C11	68.63 (10)	C14—C15—C16	108.1 (2)
C16—Fe1—C11	106.01 (11)	C14—C15—Fe1	70.27 (15)
C12—Fe1—C11	40.49 (10)	C16—C15—Fe1	69.58 (14)
C17—Fe1—C11	122.81 (11)	C14—C15—H15	126.0
C10—Fe1—C11	40.43 (10)	C16—C15—H15	126.0
C14—Fe1—C11	157.55 (11)	Fe1—C15—H15	126.0
C9—Fe1—C13	123.92 (11)	C17—C16—C15	107.5 (2)
C15—Fe1—C13	68.16 (11)	C17—C16—Fe1	70.02 (14)
C8—Fe1—C13	109.12 (10)	C15—C16—Fe1	69.37 (14)
C16—Fe1—C13	68.31 (11)	C17—C16—H16	126.2
C12—Fe1—C13	124.56 (11)	C15—C16—H16	126.2
C17—Fe1—C13	40.46 (11)	Fe1—C16—H16	126.2
C10—Fe1—C13	159.12 (11)	C16—C17—C13	108.3 (2)
C14—Fe1—C13	40.35 (11)	C16—C17—Fe1	69.42 (14)
C11—Fe1—C13	159.81 (11)	C13—C17—Fe1	69.96 (15)
N2—S1—C5	92.77 (12)	C16—C17—H17	125.9
C3—N2—S1	107.66 (17)	C13—C17—H17	125.9
C5—N4—C3	108.2 (2)	Fe1—C17—H17	125.9
N2—C3—N4	119.5 (2)	C19—C18—C5	173.8 (3)
N2—C3—C6	119.1 (2)	C18—C19—C20	178.3 (3)
N4—C3—C6	121.4 (2)	C25—C20—C21	119.6 (2)
N4—C5—C18	127.3 (2)	C25—C20—C19	121.5 (2)
N4—C5—S1	111.88 (19)	C21—C20—C19	118.9 (2)
C18—C5—S1	120.86 (19)	C22—C21—C20	119.8 (2)
C7—C6—C3	173.5 (3)	C22—C21—H21	120.1
C6—C7—C8	178.7 (3)	C20—C21—H21	120.1
C7—C8—C9	125.3 (2)	C21—C22—C23	120.4 (3)
C7—C8—C12	127.3 (2)	C21—C22—H22	119.8
C9—C8—C12	107.4 (2)	C23—C22—H22	119.8
C7—C8—Fe1	124.65 (18)	C22—C23—C24	120.1 (3)
C9—C8—Fe1	69.16 (14)	C22—C23—H23	120.0
C12—C8—Fe1	69.49 (14)	C24—C23—H23	120.0
C10—C9—C8	107.8 (2)	C25—C24—C23	120.1 (3)
C10—C9—Fe1	70.29 (15)	C25—C24—H24	120.0
C8—C9—Fe1	69.56 (14)	C23—C24—H24	120.0
C10—C9—H9	126.1	C24—C25—C20	120.1 (3)
C8—C9—H9	126.1	C24—C25—H25	119.9
Fe1—C9—H9	126.1	C20—C25—H25	119.9

Table 9. Geometric parameters of **11** (bond lengths in Å and bond angles in °) MFA

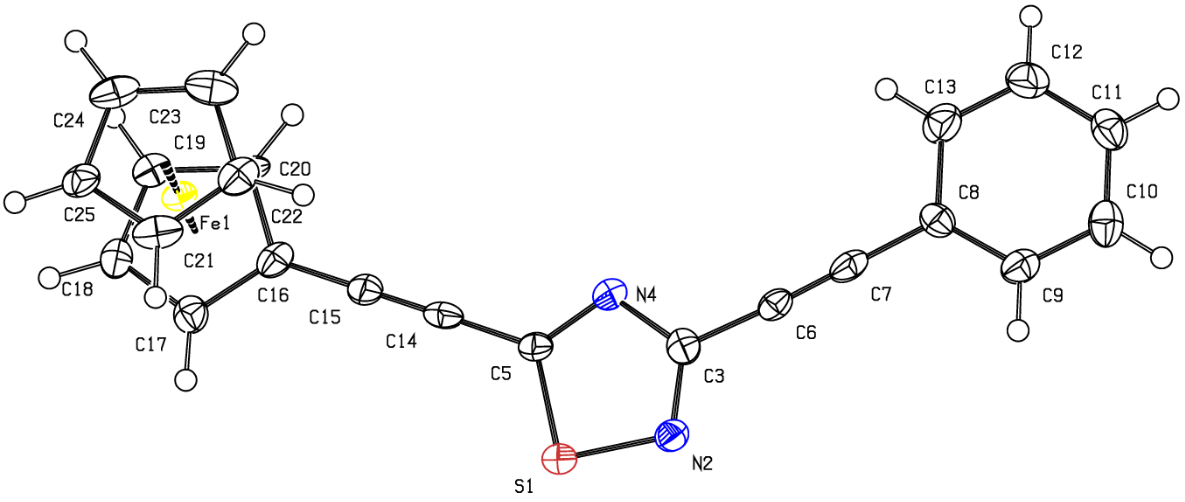

Fe1—C24	2.037 (8)	C11—H11	0.9500
Fe1—C20	2.037 (8)	C12—C13	1.372 (12)
Fe1—C25	2.043 (8)	C12—H12	0.9500
Fe1—C22	2.046 (8)	C13—H13	0.9500
Fe1—C17	2.046 (8)	C14—C15	1.210 (10)
Fe1—C16	2.049 (8)	C15—C16	1.406 (11)
Fe1—C19	2.049 (8)	C16—C17	1.439 (11)
Fe1—C21	2.054 (8)	C16—C20	1.454 (10)
Fe1—C18	2.057 (8)	C17—C18	1.418 (11)
Fe1—C23	2.063 (8)	C17—H17	1.0000
S1—N2	1.655 (6)	C18—C19	1.408 (11)
S1—C5	1.730 (8)	C18—H18	1.0000
N2—C3	1.330 (10)	C19—C20	1.415 (11)
N4—C5	1.299 (9)	C19—H19	1.0000
N4—C3	1.357 (9)	C20—H20	1.0000
C3—C6	1.444 (11)	C21—C22	1.408 (12)
C5—C14	1.434 (11)	C21—C25	1.413 (11)
C6—C7	1.190 (11)	C21—H21	1.0000
C7—C8	1.430 (11)	C22—C23	1.438 (12)
C8—C9	1.380 (11)	C22—H22	1.0000
C8—C13	1.431 (12)	C23—C24	1.409 (12)
C9—C10	1.371 (11)	C23—H23	1.0000
C9—H9	0.9500	C24—C25	1.418 (12)
C10—C11	1.395 (12)	C24—H24	1.0000
C10—H10	0.9500	C25—H25	1.0000
C11—C12	1.392 (12)		
C24—Fe1—C20	121.2 (3)	C13—C12—C11	120.3 (9)
C24—Fe1—C25	40.7 (3)	C13—C12—H12	119.9
C20—Fe1—C25	158.0 (3)	C11—C12—H12	119.9
C24—Fe1—C22	68.1 (3)	C12—C13—C8	119.9 (8)

C20—Fe1—C22	122.3 (3)	C12—C13—H13	120.0
C25—Fe1—C22	67.8 (3)	C8—C13—H13	120.0
C24—Fe1—C17	157.6 (3)	C15—C14—C5	179.1 (9)
C20—Fe1—C17	69.5 (3)	C14—C15—C16	178.0 (9)
C25—Fe1—C17	122.8 (3)	C15—C16—C17	126.1 (8)
C22—Fe1—C17	125.2 (3)	C15—C16—C20	126.7 (8)
C24—Fe1—C16	159.0 (4)	C17—C16—C20	107.2 (7)
C20—Fe1—C16	41.7 (3)	C15—C16—Fe1	126.5 (6)
C25—Fe1—C16	159.1 (3)	C17—C16—Fe1	69.3 (5)
C22—Fe1—C16	108.5 (3)	C20—C16—Fe1	68.7 (4)
C17—Fe1—C16	41.1 (3)	C18—C17—C16	107.6 (7)
C24—Fe1—C19	106.1 (3)	C18—C17—Fe1	70.2 (5)
C20—Fe1—C19	40.5 (3)	C16—C17—Fe1	69.5 (5)
C25—Fe1—C19	122.9 (3)	C18—C17—H17	126.2
C22—Fe1—C19	157.4 (3)	C16—C17—H17	126.2
C17—Fe1—C19	68.3 (3)	Fe1—C17—H17	126.2
C16—Fe1—C19	68.6 (3)	C19—C18—C17	108.9 (7)
C24—Fe1—C21	68.1 (3)	C19—C18—Fe1	69.7 (4)
C20—Fe1—C21	159.0 (3)	C17—C18—Fe1	69.4 (5)
C25—Fe1—C21	40.3 (3)	C19—C18—H18	125.6
C22—Fe1—C21	40.2 (3)	C17—C18—H18	125.6
C17—Fe1—C21	109.3 (3)	Fe1—C18—H18	125.6
C16—Fe1—C21	123.6 (3)	C18—C19—C20	109.1 (7)
C19—Fe1—C21	160.0 (3)	C18—C19—Fe1	70.2 (5)
C24—Fe1—C18	121.5 (3)	C20—C19—Fe1	69.3 (4)
C20—Fe1—C18	68.3 (3)	C18—C19—H19	125.4
C25—Fe1—C18	108.2 (3)	C20—C19—H19	125.4
C22—Fe1—C18	161.5 (3)	Fe1—C19—H19	125.4
C17—Fe1—C18	40.4 (3)	C19—C20—C16	107.2 (7)
C16—Fe1—C18	68.3 (3)	C19—C20—Fe1	70.2 (4)
C19—Fe1—C18	40.1 (3)	C16—C20—Fe1	69.6 (4)
C21—Fe1—C18	125.2 (3)	C19—C20—H20	126.4
C24—Fe1—C23	40.2 (3)	C16—C20—H20	126.4
C20—Fe1—C23	105.9 (3)	Fe1—C20—H20	126.4
C25—Fe1—C23	68.1 (4)	C22—C21—C25	108.0 (8)
C22—Fe1—C23	41.0 (3)	C22—C21—Fe1	69.6 (5)
C17—Fe1—C23	161.4 (3)	C25—C21—Fe1	69.4 (5)
C16—Fe1—C23	123.7 (4)	C22—C21—H21	126.0
C19—Fe1—C23	120.6 (3)	C25—C21—H21	126.0
C21—Fe1—C23	68.2 (3)	Fe1—C21—H21	126.0
C18—Fe1—C23	156.2 (3)	C21—C22—C23	108.4 (7)
N2—S1—C5	92.3 (3)	C21—C22—Fe1	70.2 (5)
C3—N2—S1	106.5 (5)	C23—C22—Fe1	70.1 (5)
C5—N4—C3	108.3 (7)	C21—C22—H22	125.8
N2—C3—N4	120.6 (7)	C23—C22—H22	125.8
N2—C3—C6	120.2 (7)	Fe1—C22—H22	125.8

N4—C3—C6	119.2 (7)	C24—C23—C22	106.8 (8)
N4—C5—C14	124.6 (7)	C24—C23—Fe1	68.9 (5)
N4—C5—S1	112.3 (5)	C22—C23—Fe1	68.9 (5)
C14—C5—S1	123.1 (6)	C24—C23—H23	126.6
C7—C6—C3	174.8 (8)	C22—C23—H23	126.6
C6—C7—C8	179.8 (9)	Fe1—C23—H23	126.6
C9—C8—C7	122.2 (8)	C23—C24—C25	108.7 (8)
C9—C8—C13	117.9 (8)	C23—C24—Fe1	70.9 (5)
C7—C8—C13	119.9 (7)	C25—C24—Fe1	69.9 (5)
C10—C9—C8	122.6 (8)	C23—C24—H24	125.6
C10—C9—H9	118.7	C25—C24—H24	125.6
C8—C9—H9	118.7	Fe1—C24—H24	125.6
C9—C10—C11	118.8 (8)	C21—C25—C24	108.1 (8)
C9—C10—H10	120.6	C21—C25—Fe1	70.3 (5)
C11—C10—H10	120.6	C24—C25—Fe1	69.4 (5)
C12—C11—C10	120.4 (8)	C21—C25—H25	126.0
C12—C11—H11	119.8	C24—C25—H25	126.0
C10—C11—H11	119.8	Fe1—C25—H25	126.0
C5—S1—N2—C3	0.2 (6)	Fe1—C18—C19—C20	58.5 (5)
S1—N2—C3—N4	-0.2 (9)	C17—C18—C19—Fe1	-58.5 (6)
S1—N2—C3—C6	-179.1 (6)	C18—C19—C20—C16	1.0 (9)
C5—N4—C3—N2	0.0 (10)	Fe1—C19—C20—C16	60.1 (5)
C5—N4—C3—C6	178.9 (7)	C18—C19—C20—Fe1	-59.1 (5)
C3—N4—C5—C14	178.2 (7)	C15—C16—C20—C19	179.2 (8)
C3—N4—C5—S1	0.2 (8)	C17—C16—C20—C19	-1.6 (8)
N2—S1—C5—N4	-0.3 (6)	Fe1—C16—C20—C19	-60.5 (5)
N2—S1—C5—C14	-178.3 (7)	C15—C16—C20—Fe1	-120.3 (8)
C7—C8—C9—C10	177.9 (8)	C17—C16—C20—Fe1	58.8 (5)
C13—C8—C9—C10	-1.0 (13)	C25—C21—C22—C23	1.0 (9)
C8—C9—C10—C11	0.5 (13)	Fe1—C21—C22—C23	60.0 (6)
C9—C10—C11—C12	-0.5 (13)	C25—C21—C22—Fe1	-59.0 (6)
C10—C11—C12—C13	1.0 (14)	C21—C22—C23—C24	-1.4 (9)
C11—C12—C13—C8	-1.4 (14)	Fe1—C22—C23—C24	58.7 (6)
C9—C8—C13—C12	1.4 (13)	C21—C22—C23—Fe1	-60.1 (6)
C7—C8—C13—C12	-177.5 (8)	C22—C23—C24—C25	1.2 (9)
C15—C16—C17—C18	-179.2 (8)	Fe1—C23—C24—C25	59.9 (6)
C20—C16—C17—C18	1.7 (9)	C22—C23—C24—Fe1	-58.7 (6)
Fe1—C16—C17—C18	60.1 (5)	C22—C21—C25—C24	-0.2 (9)
C15—C16—C17—Fe1	120.7 (8)	Fe1—C21—C25—C24	-59.3 (6)
C20—C16—C17—Fe1	-58.4 (5)	C22—C21—C25—Fe1	59.1 (6)
C16—C17—C18—C19	-1.1 (9)	C23—C24—C25—C21	-0.6 (9)
Fe1—C17—C18—C19	58.6 (6)	Fe1—C24—C25—C21	59.9 (6)
C16—C17—C18—Fe1	-59.7 (6)	C23—C24—C25—Fe1	-60.5 (6)
C17—C18—C19—C20	0.0 (9)		

Table 10. van der Waals radii (r_w) and values of experimental halide interactions.

	r_w[1] (Cl = 1.75) (Å)	Σ (r_{wN} + r_{wI}) (Cl···N) (Å)	Σ (r_{wS} + r_{wI}) (Cl···S) (Å)	Σ (r_{wI} + r_{wI}) (Cl···Cl)[2–4] (Å)	Experimental Range[a] (Å) (Cl···N) (Å)	Experimental Range[a] (Å) (Cl···S) (Å)	Experimental Range[a] (Å) (Cl···Cl) (Å)
**N**	1.55	3.30	—	—	2.08 – 3.29	—	—
**S**	1.80	—	3.55	—	—	3.23 – 3.53	—
**Cl**	1.75	—	—	3.52	—	—	3.05 – 3.5

[a] based on a CCDC search[4]

	r_w[1] (I = 1.98) (Å)	Σ (r_{wN} + r_{wI}) (I···N) (Å)	Σ (r_{wS} + r_{wI}) (I···S) (Å)	Σ (r_{wI} + r_{wI}) (I···I) (Å)	Experimental Range[a] (Å) (I···N) (Å)	Experimental Range (Å) (I···S) (Å)	Experimental Range (Å) (I···I) (Å)
**N**	1.55	3.53	—	—	2.78 − 3.52	—	—
**S**	1.80	—	3.78	—	—	3.16 − 3.76	—
**I**	1.98	—	—	3.96	—	—	3.15 − 3.96

[a] based on a CCDC search[5]

[1] Mantina, M.; Chamberlin, A.C.; Valero, L.; Cramer, C.J.; Truhlar, D.G., *J. Phys. Chem. A* **2009**, *113*, 5806.
[2] Desiraju, G. R.; Parthasarathy, R., The nature of halogen.cntdot..cntdot..cntdot.halogen interactions: are short halogen contacts due to specific attractive forces or due to close packing of nonspherical atoms? *J. Am. Chem. Soc.* **1989,** *111* (23), 8725-8726.
[3] Desiraju Gautam, R.; Ho, P. S.; Kloo, L.; Legon Anthony, C.; Marquardt, R.; Metrangolo, P.; Politzer, P.; Resnati, G.; Rissanen, K., Definition of the halogen bond (IUPAC Recommendations 2013). In *Pure Appl. Chem.*, **2013**; Vol. 85, p 1711.
[4] Metrangolo, P.; Resnati, G., Type II halogen...halogen contacts are halogen bonds. *IUCrJ* **2014,** 1 (1), 5-7.
[5] Allen, F. H., The Cambridge Structural Database: a quarter of a million crystal structures and rising. Acta Crystallogr., Sect. B **2002**, B58, 380-388.

Table 11. Intermolecular interactions of **4**, **5**, and **6**

	**4** X = Cl	**5** X = Cl	**6** X = I
**X···N (Å)**	Closest is 3.72 (above average)	Closest is 3.57 (above average)	Closest is 4.05 (above average)
**X···S (Å)**	Closest is 4.05 (above average)	----	---
**X···X (Å)**	Closest is 3.79 (above average)	Closest is 3.58 (above average)	Closest is 4.64 (above average)
**S···N (Å)** (2.78–3.35 Å)[a]	Closest is 3.57 (above average)	Closest is 3.70 (above average)	Closest is 4.17 (above average)
**S···S (Å)** (3.15–3.69 Å)[a]	Closest is 3.89 (above average)	3.59	Closest is 3.97 (above average)
**Distance between planes (Å)**	3.61<br>3.38	3.48	3.46
**Tdzi to Fc (centroid to centroid) distance (Å)**	d = 3.94, R = 2.02<br>d = 3.56, R = 1.12	d = 3.67, R = 1.17	d = 3.67, R = 1.22
**C≡C to C≡C (centroid to centroid) Distance (Å)**	---	d = 3.71, R = 1.29	d = 3.68, R = 1.25
**Edge to Face (Fc)C−H···*π* (Fc) (Å)**	2.69	---	2.77
**Edge to Face (Fc)C−H···*π* (C≡C) (Å)**		2.96, 3.01, 3.22	2.99, 3.01, 3.23
**C−H···X (Å)**	2.75 – 3.41	2.95	3.17–3.20, 3.33, 3.39
**C−H···N (Å)**	2.45 – 3.03	2.79, 2.80	2.92, 2.94
**C−H···S (Å)**	2.95	---	2.98
**Closest H···H Contacts (Å)**	2.19, 2.24, 2.38	2.29	2.39

[a] based on a CCDC search

Table 12. Intermolecular interactions of **8**, **10**, and **11**

	**8**	**10**	**11**
**S⋯N (Å)** (2.78–3.35 Å)[a]	Closest is 3.94 (above average)	Closest is 3.52 (above average)	---
**S⋯S (Å)** (3.15–3.69 Å)[a]	Closest is 3.92 (above average)	Closest is 3.86 (above average)	Closest is 3.82 (above average)
**Distance between planes (Å)**	3.66	3.31	3.50
**Tdzi to Fc (centroid to centroid) distance (Å)**	d = 3.84, R =1.16	d = 3.71, R = 1.68	d = 3.82, R =1.52
**C≡C to C≡C (centroid to centroid) Distance (Å)**	d = 3.77, R =0.90	d = 3.67, R = 1.59	d = 3.87, R = 1.65
**Edge to Face (Fc)C−H⋯*π* (Fc) (Å)**	2.87, 3.08, 3.12	---	---
**Edge to Face (Fc)C−H⋯*π* (C≡C) (Å)**	2.93, 2.98, 3.02, 3.05, 3.12	2.80, 2.93, 3.05, 3.18	2.99, 3.38
**Edge to Face (Fc)C−H⋯*π* (Ph) (Å)**		2.82	Closest is 3.73 (above average)
**Edge to Face (Ph)C−H⋯*π* (C≡C) (Å)**	---	2.85, 2.93, 3.26	Closest is 3.71 (above average)
**Edge to Face (Ph)C−H⋯*π* (Ph) (Å)**	---	2.78	2.73, 3.49
**C−H⋯N (Å)**	---	---	---
**C−H⋯S (Å)**	2.98	---	---
**Closest H⋯H Contacts (Å)**	---	2.27, 2.38	2.29, 2.36

[a] based on a CCDC search

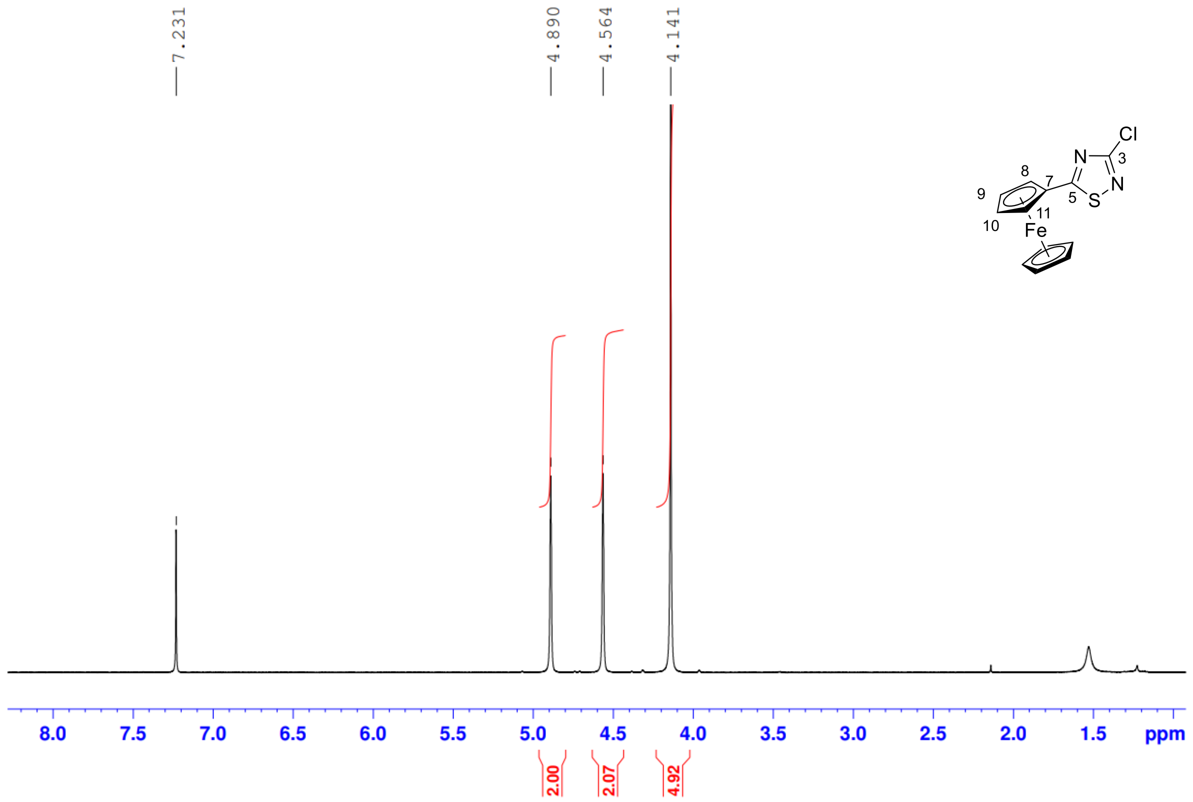

Figure S17. ^{1}H NMR spectrum of compound **4** in $CDCl_3$

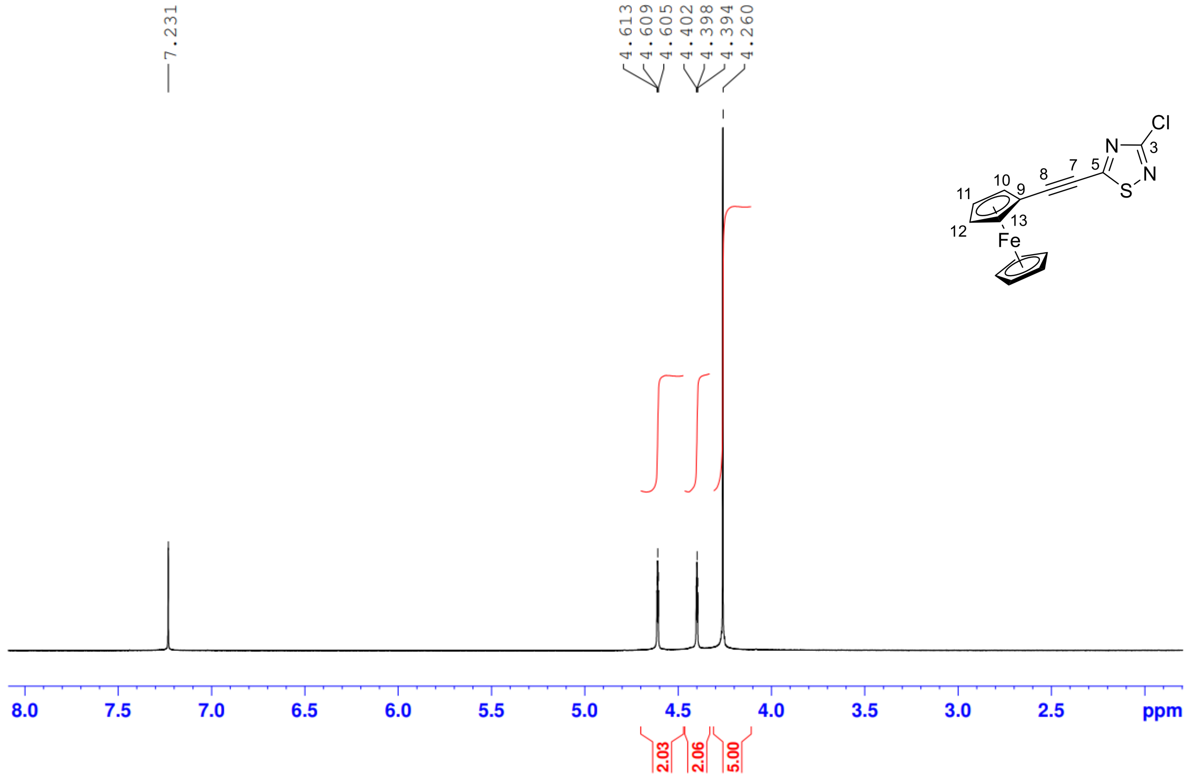

Figure S18. ^{1}H NMR spectrum of compound **5** in $CDCl_3$

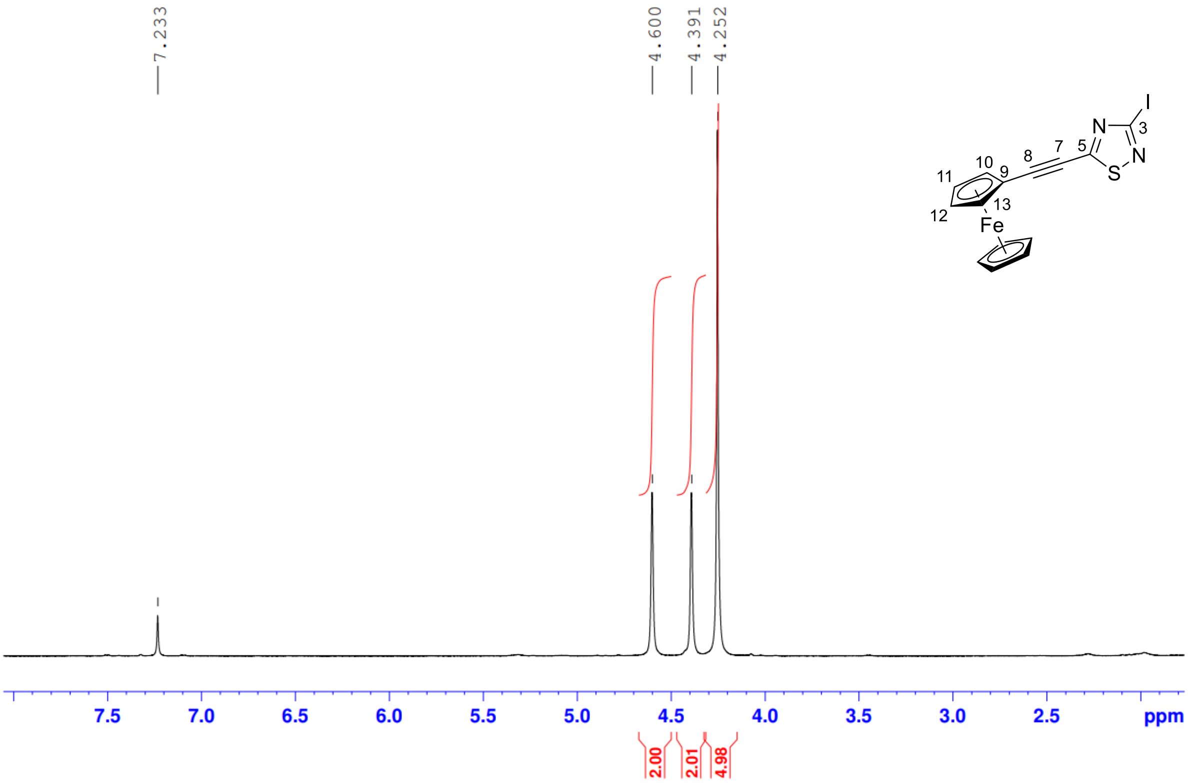

Figure S19. ^{1}H NMR spectrum of compound **6** in $CDCl_3$

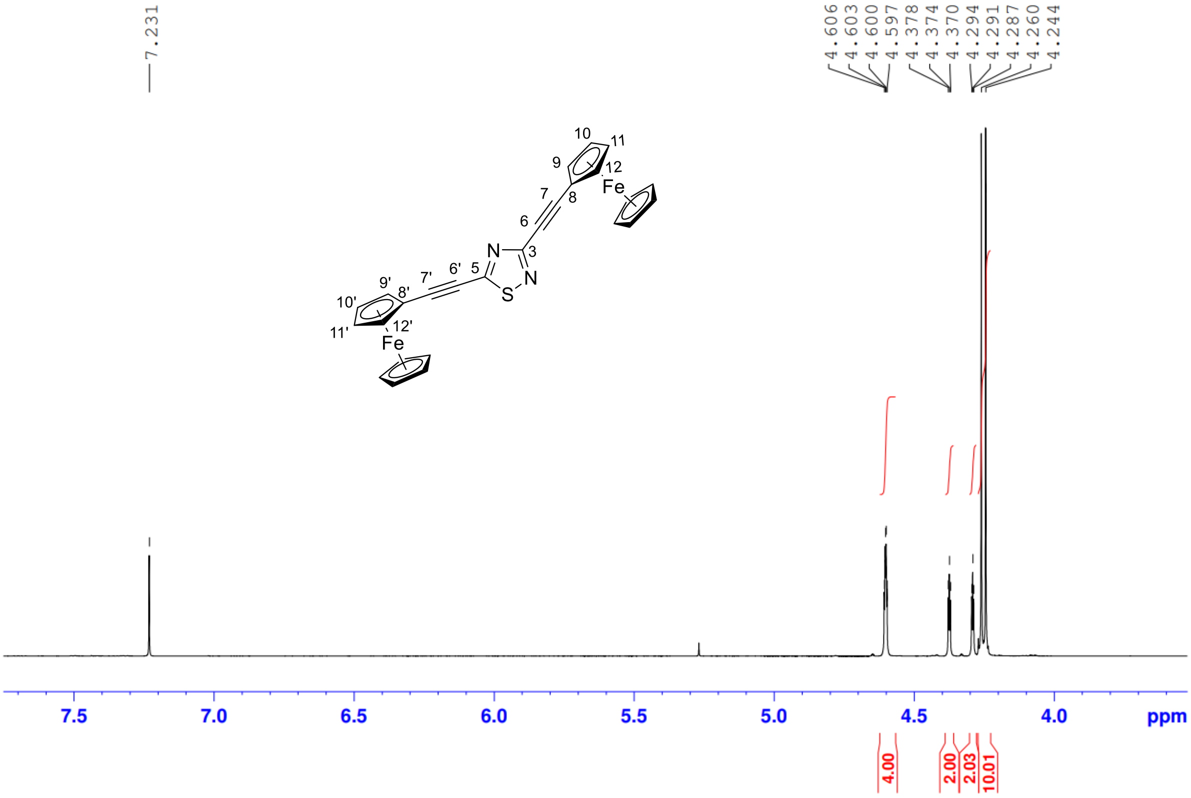

Figure S20. ^{1}H NMR spectrum of compound **8** in $CDCl_3$

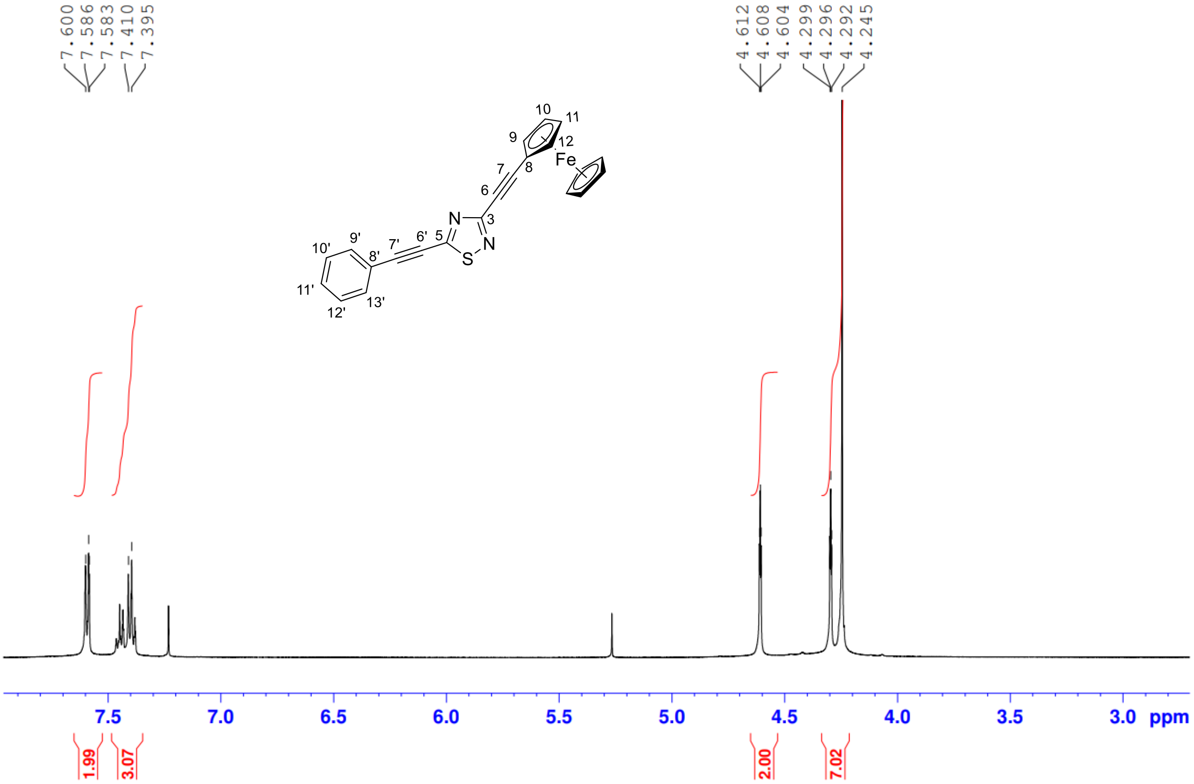

Figure S21. ^{1}H NMR spectrum of compound **10** in $CDCl_3$

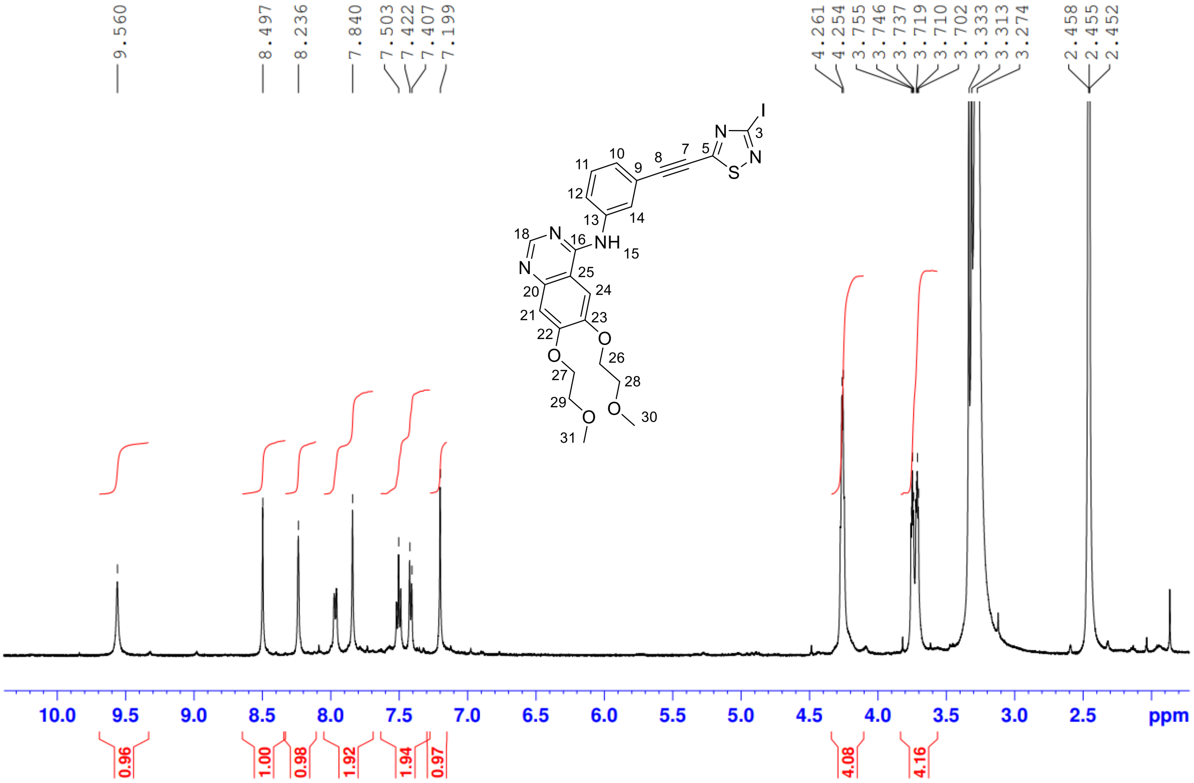

Figure S22. ^{1}H NMR spectrum of compound **13** in DMSO-d_6

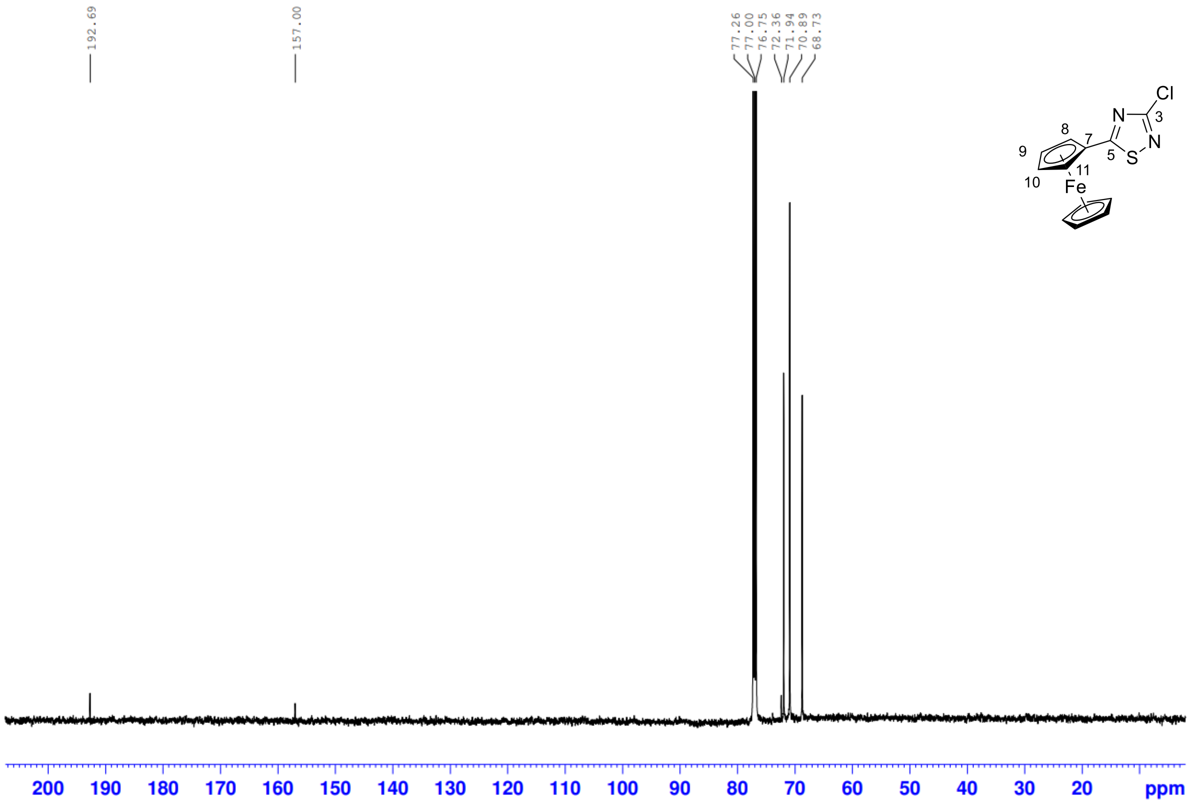

Figure S23. ^{13}C NMR spectrum of compound **4** in $CDCl_3$

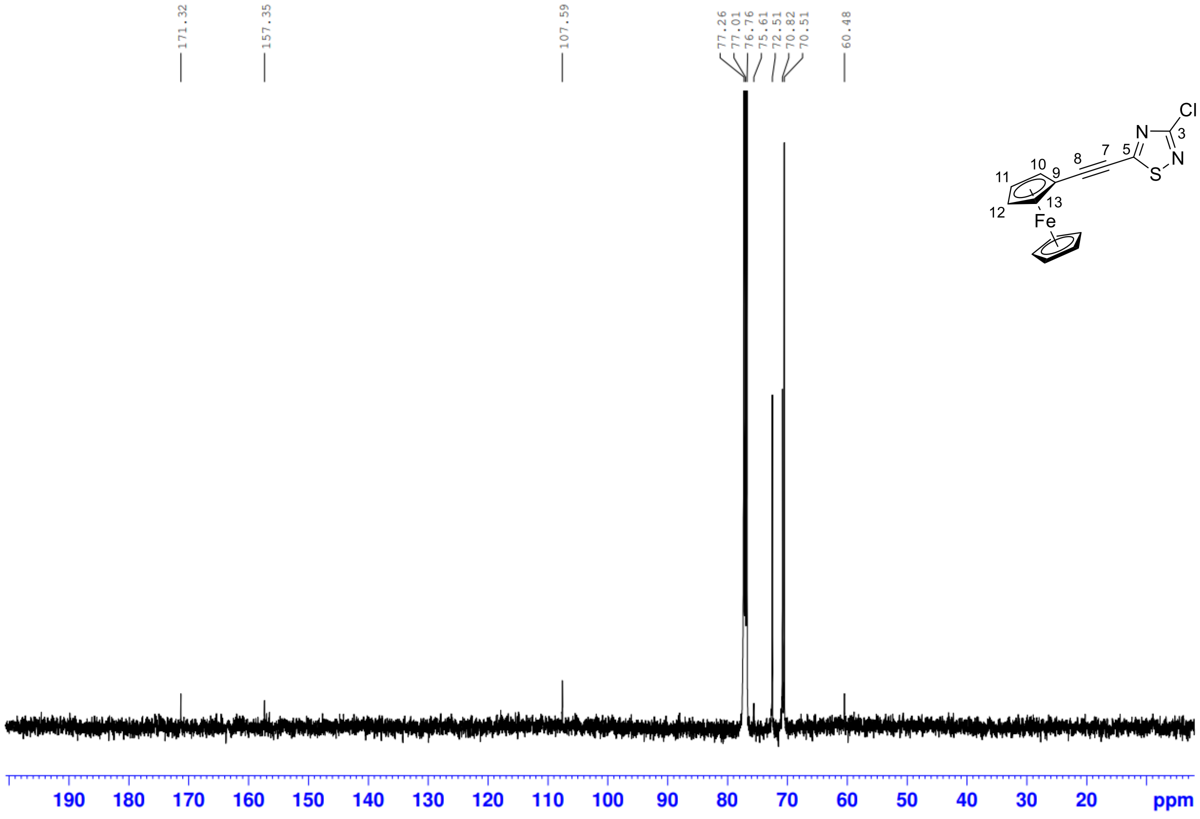

Figure S24. ^{13}C NMR spectrum of compound **5** in $CDCl_3$

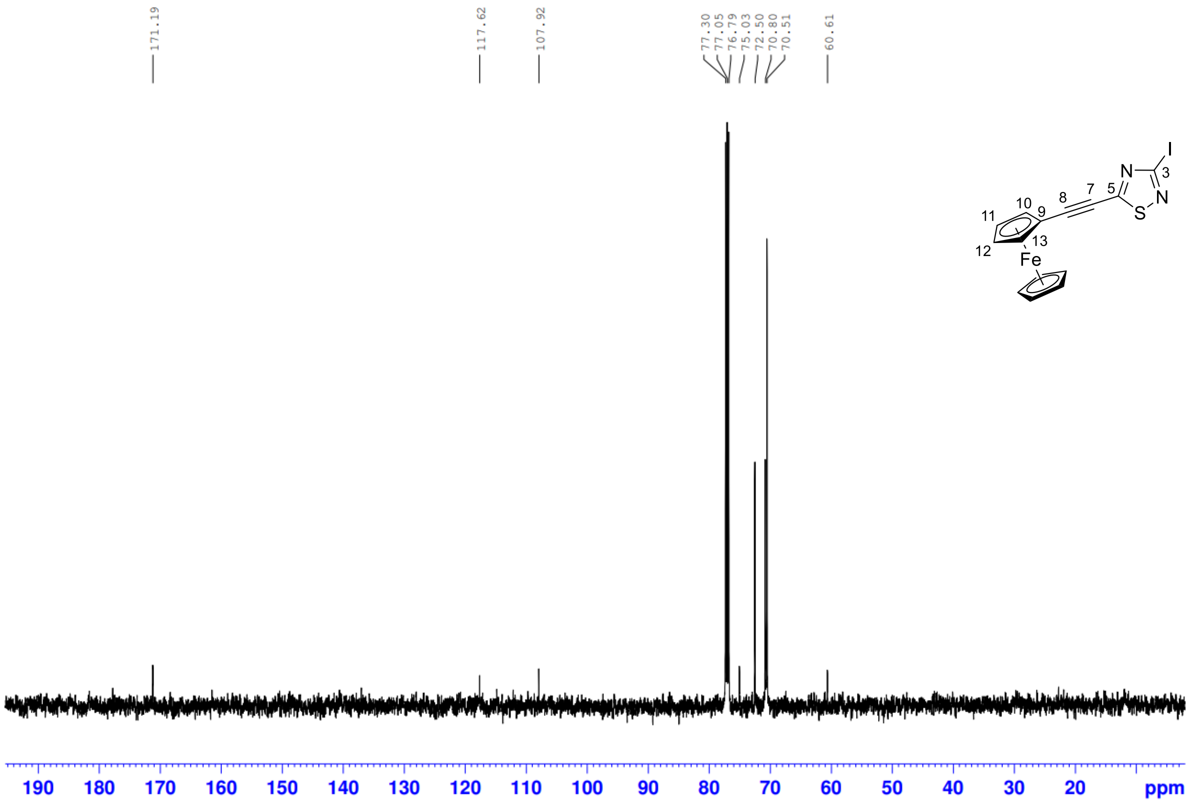

Figure S25. ^{13}C NMR spectrum of compound **6** in $CDCl_3$

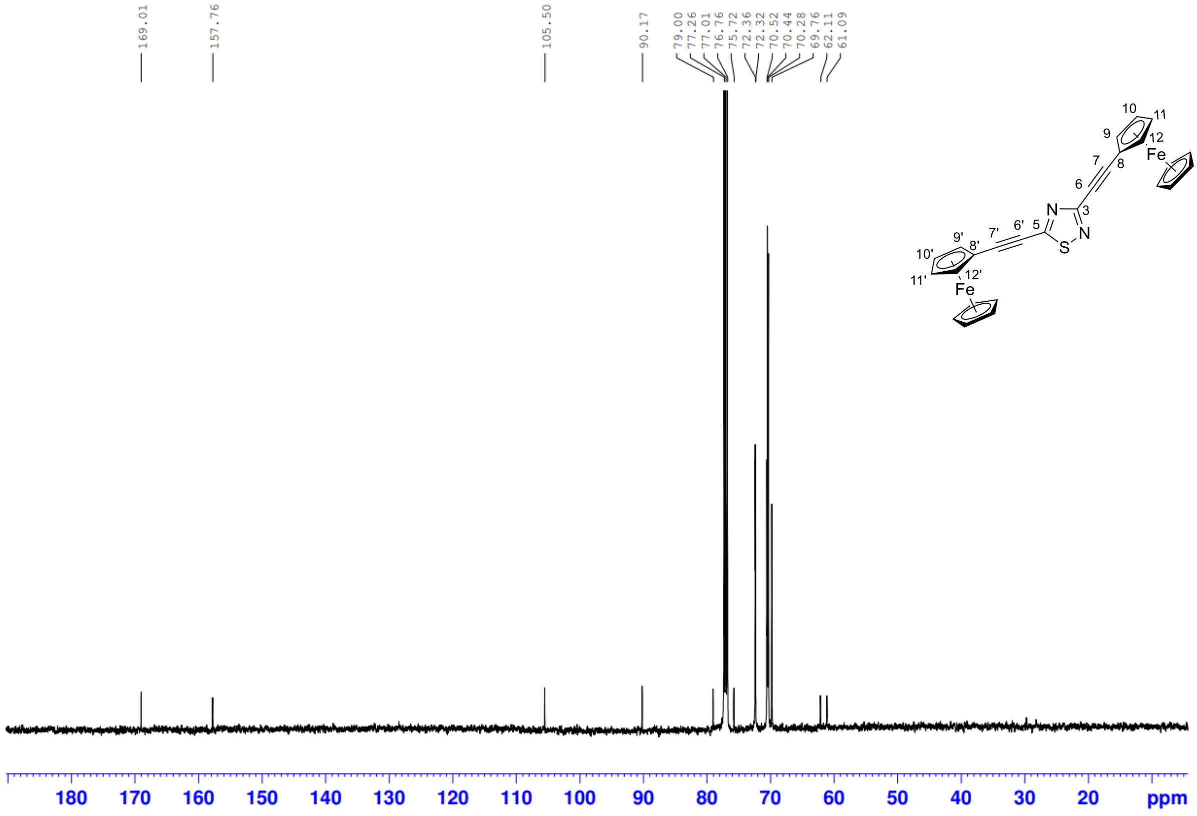

Figure S26. ^{13}C NMR spectrum of compound **8** in $CDCl_3$

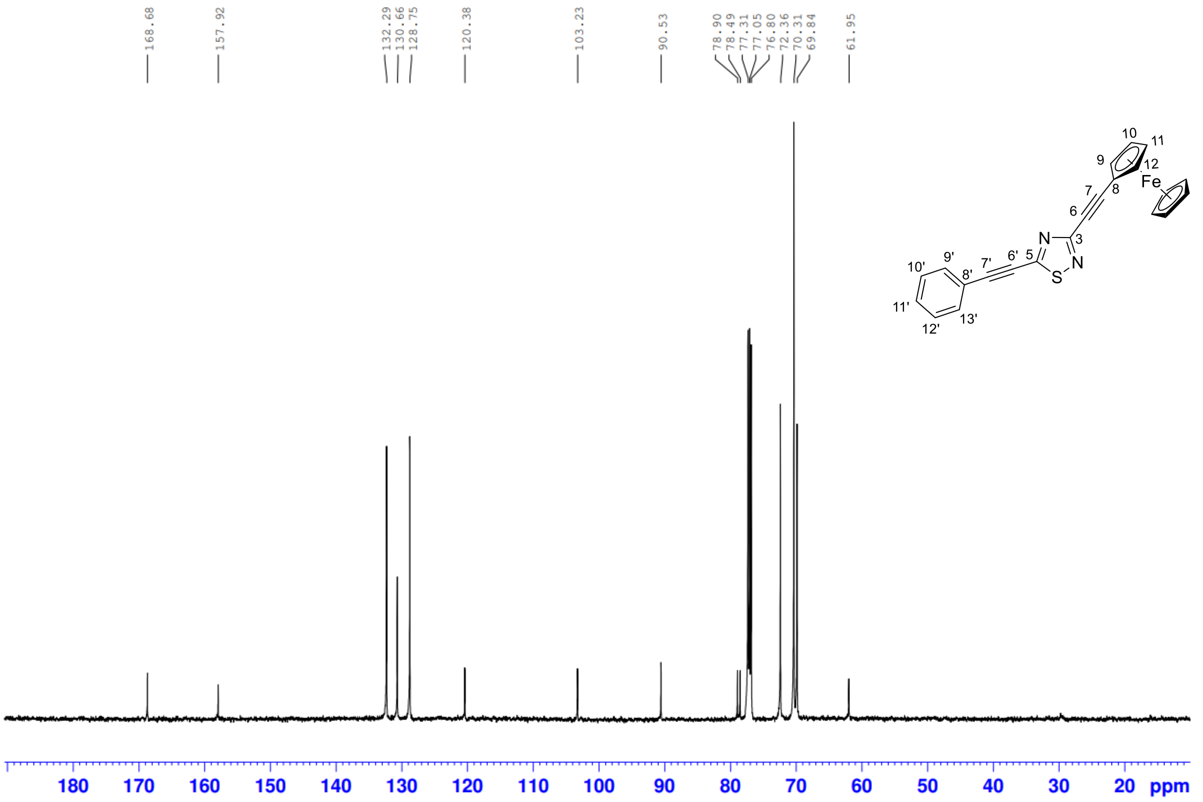

Figure S27. ^{13}C NMR spectrum of compound **10** in $CDCl_3$

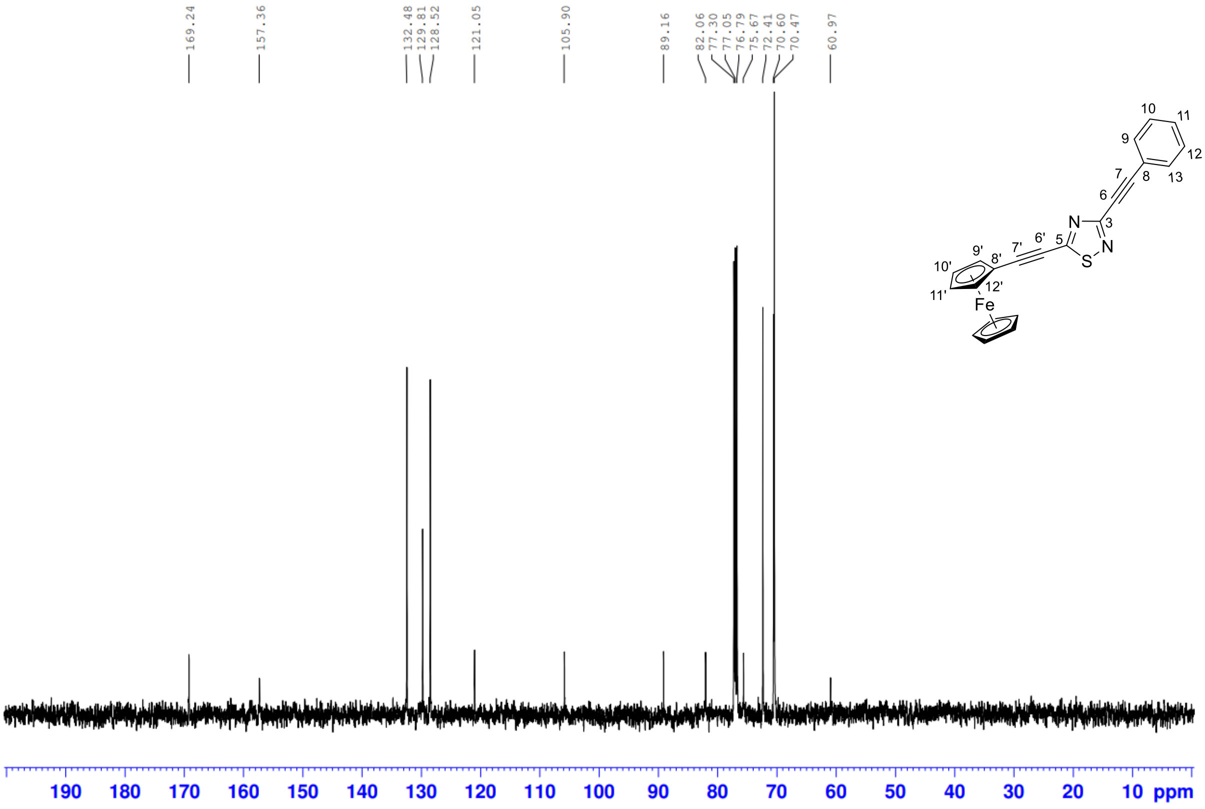

Figure S28. ^{13}C NMR spectrum of compound **11** in $CDCl_3$

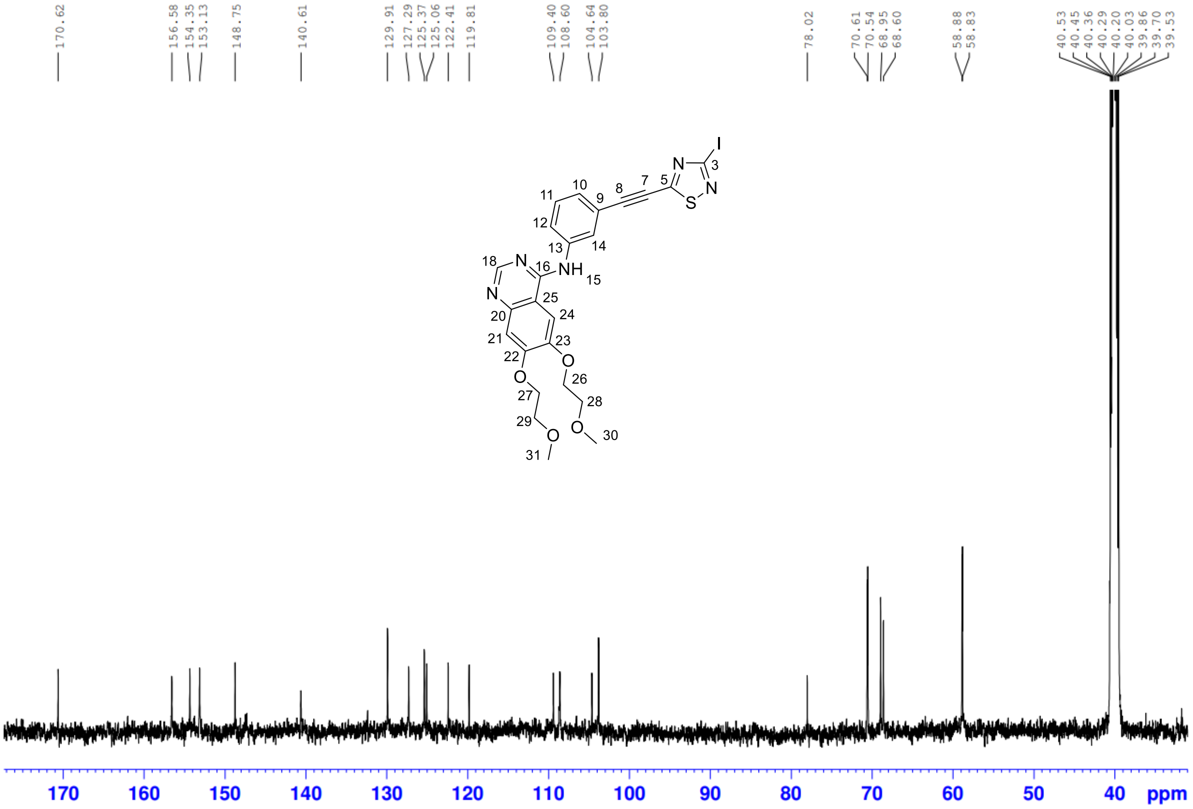

Figure S29. ^{13}C NMR spectrum of compound **13** in DMSO-d_6

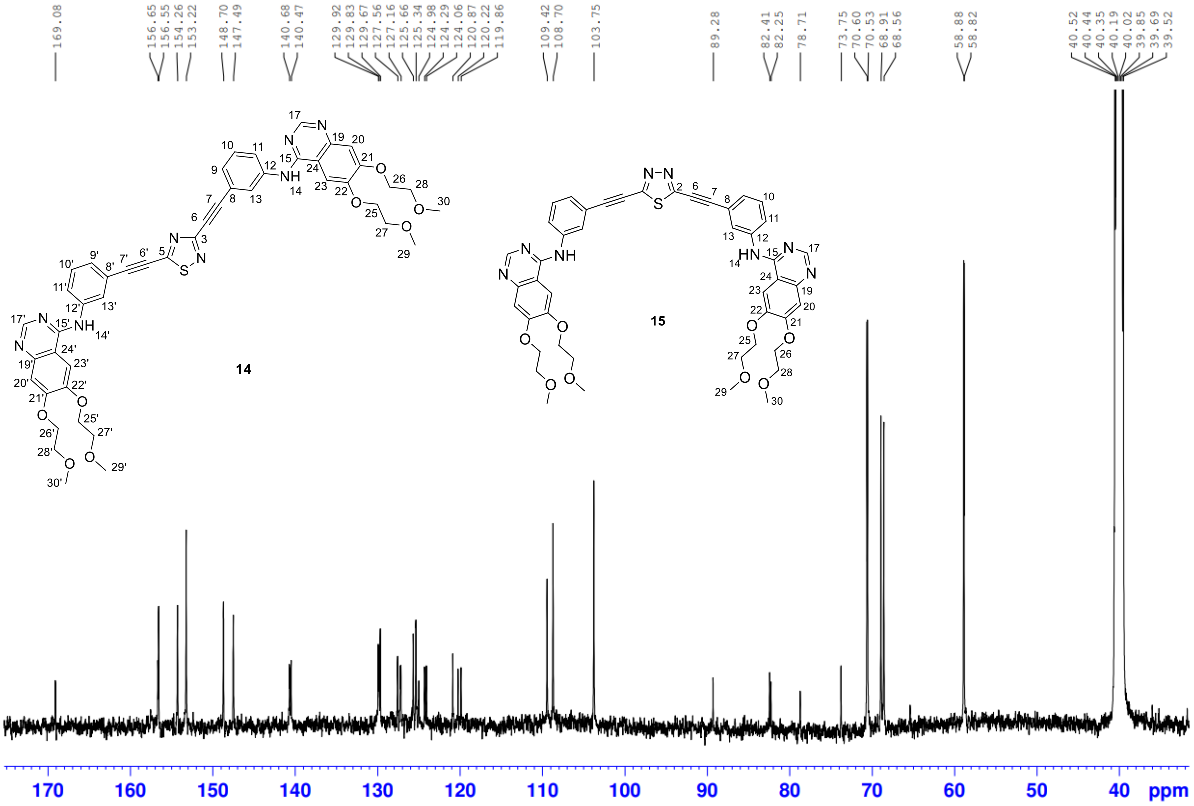

Figure S30. ^{13}C NMR spectrum of compounds **14**/**15** in DMSO-d_6

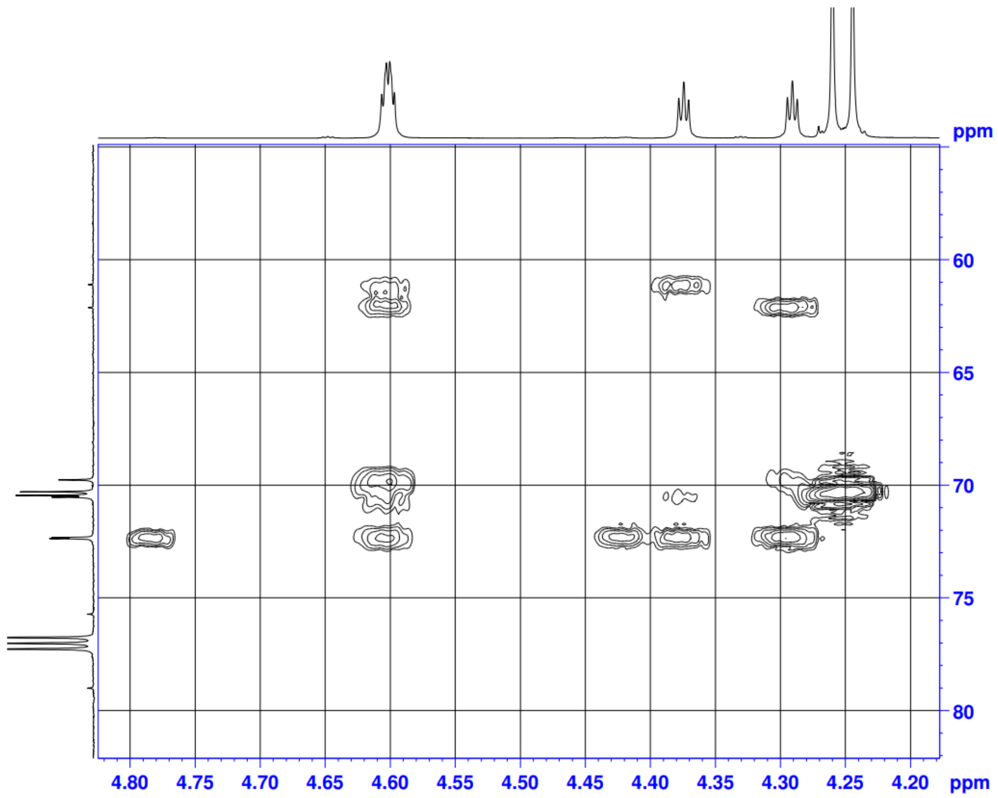

Figure S31. HMBC NMR spectrum of compound **8** in $CDCl_3$

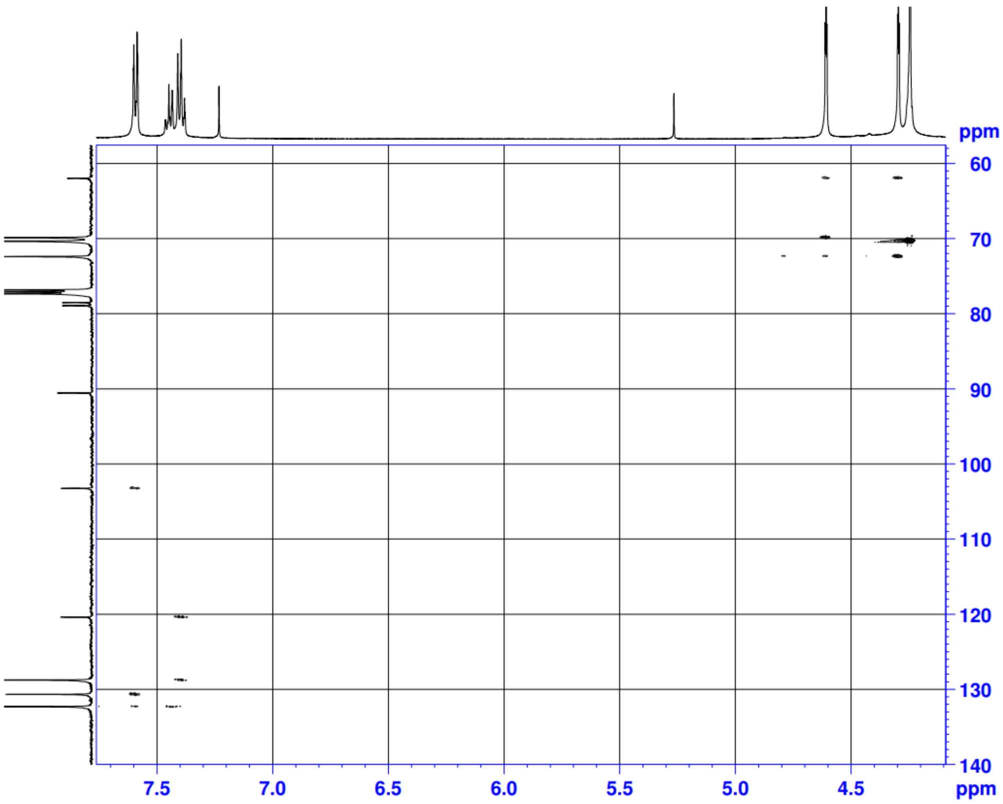

Figure S32. HMBC NMR spectrum of compound **10** in $CDCl_3$

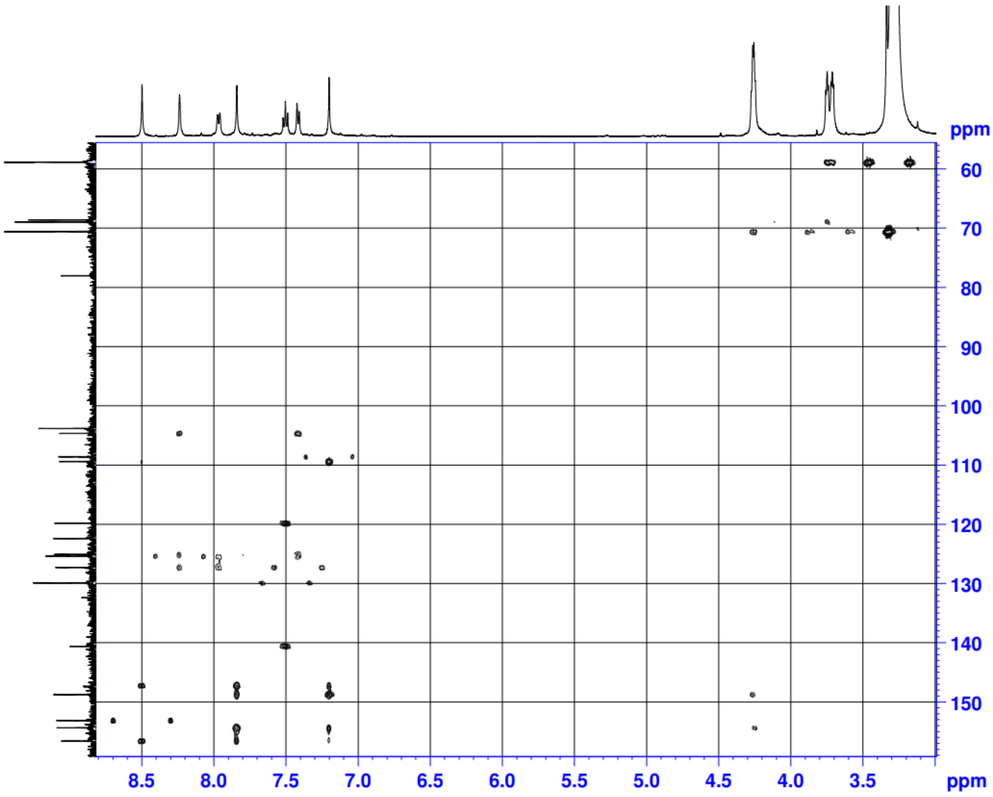

Figure S33. HMBC NMR spectrum of compound **13** DMSO-d$_6$

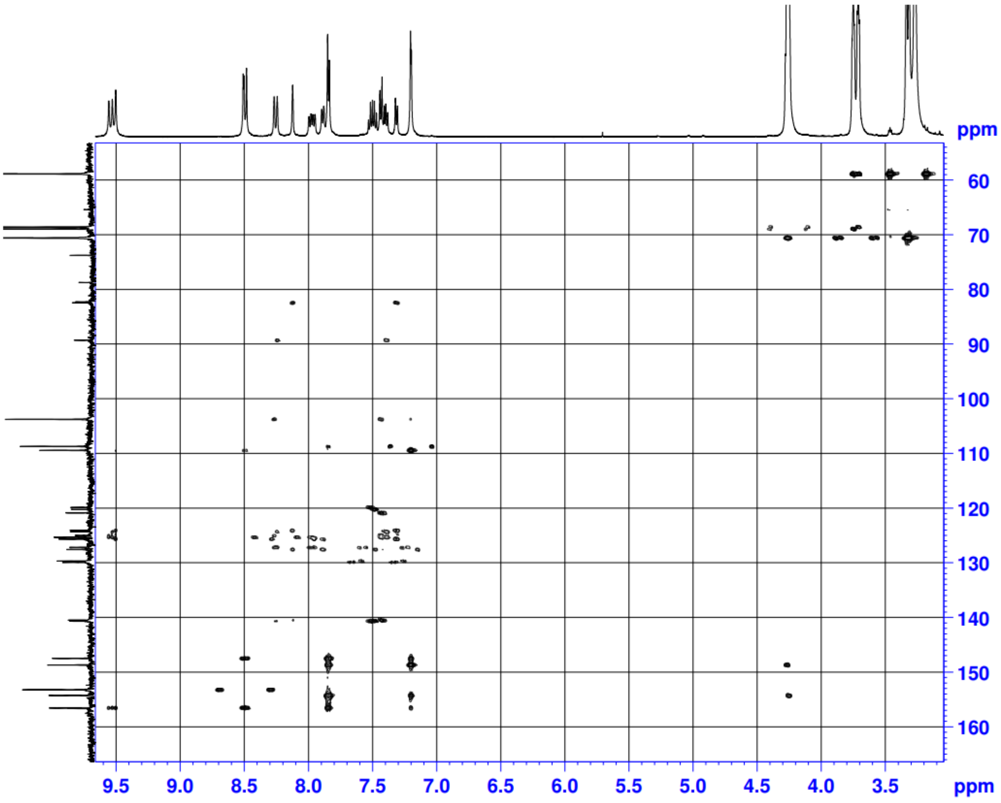

Figure S34. HMBC NMR spectrum of compounds **14**/**15** in DMSO-d$_6$

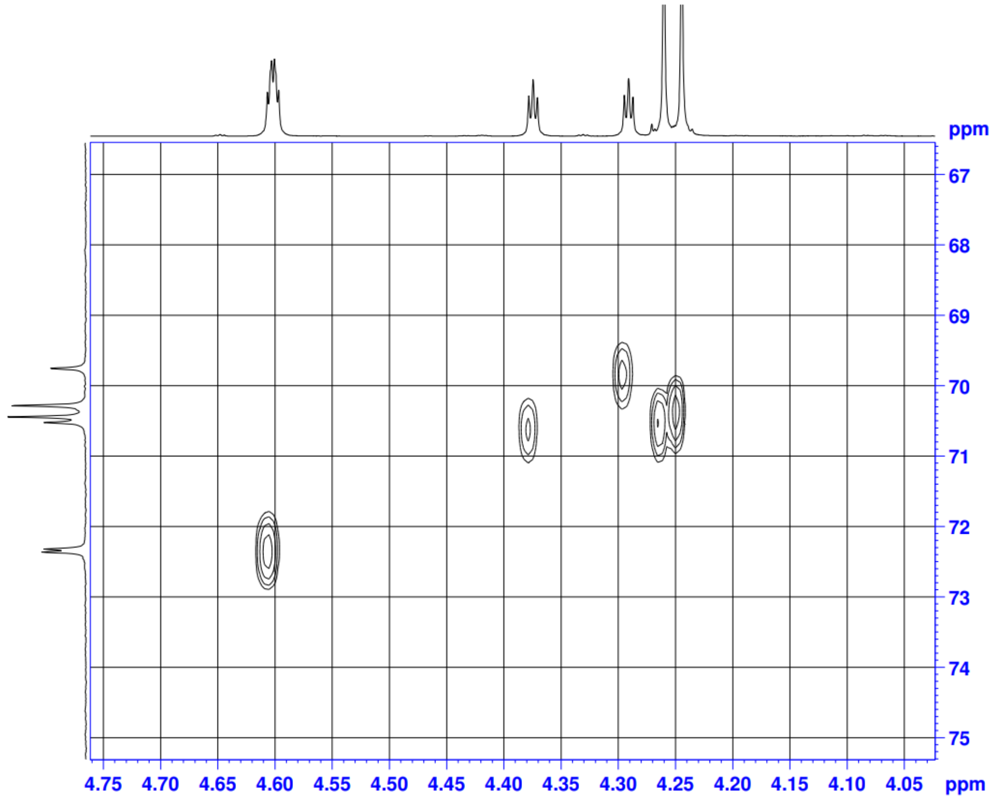

Figure S35. HSQC NMR spectrum of compound **8** in $CDCl_3$

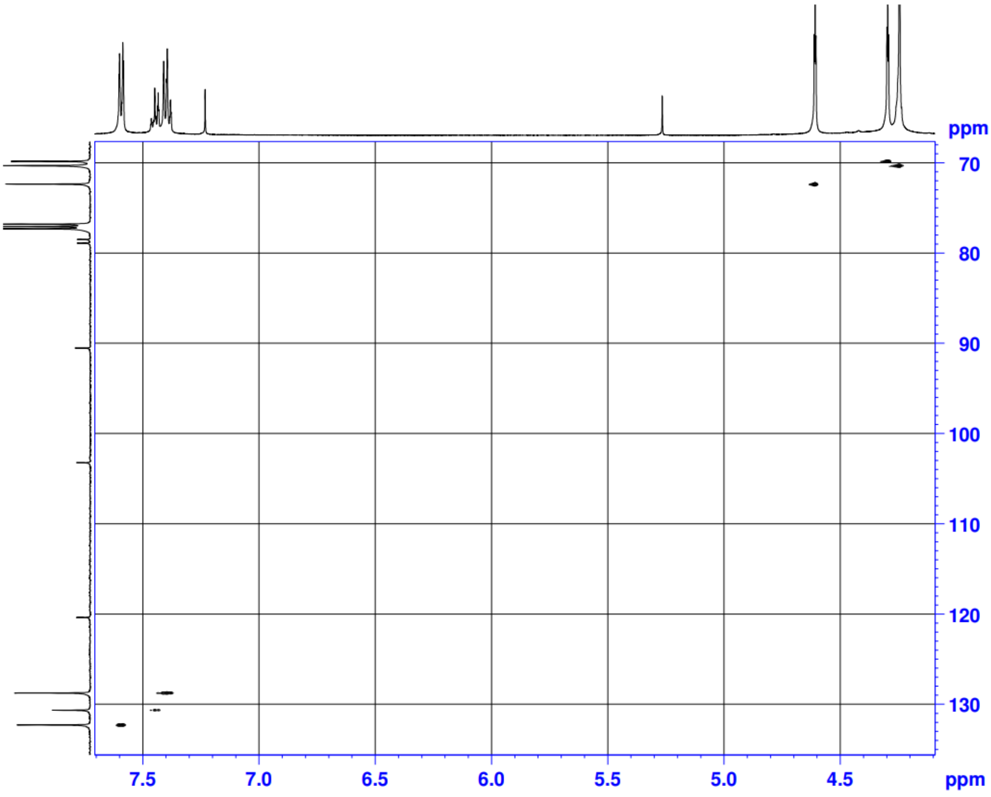

Figure S36. HSQC NMR spectrum of compound **10** in $CDCl_3$

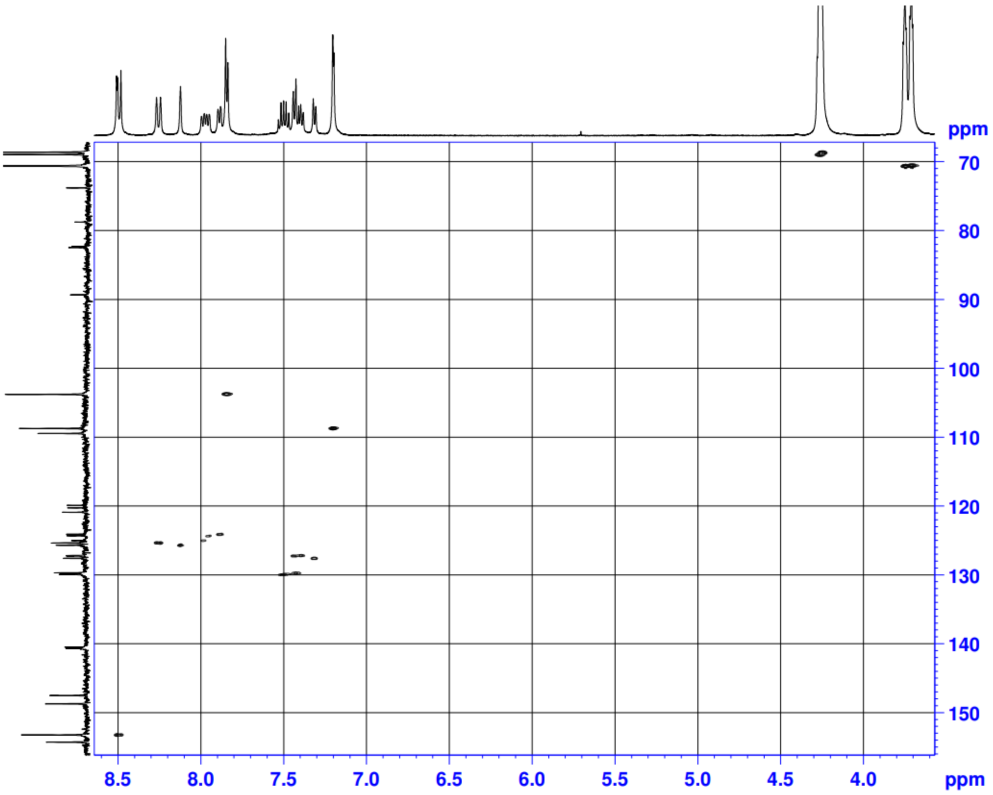

Figure S37. HSQC NMR spectrum of compound **14**/**15** in DMSO-d_6

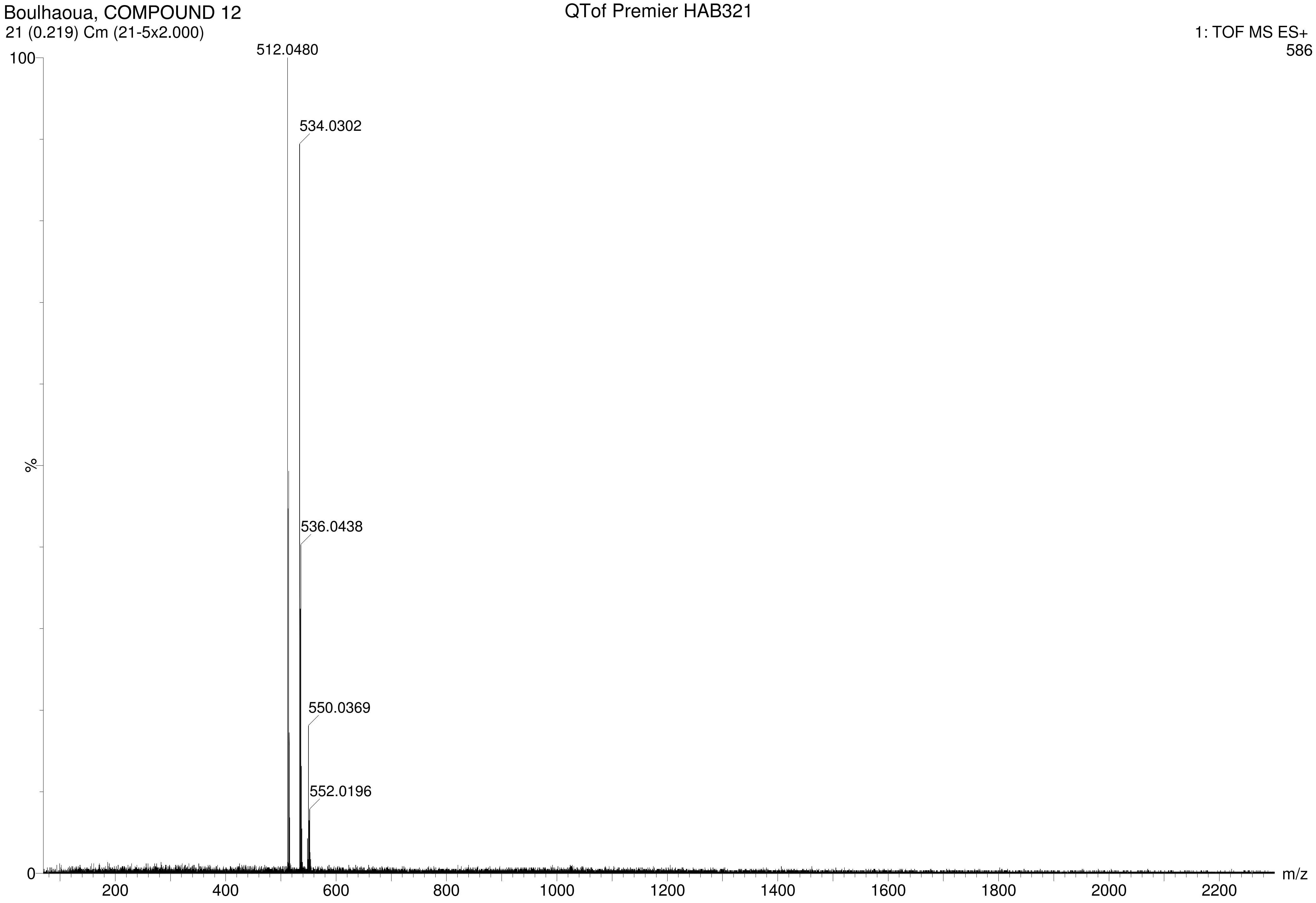

Boulhaoua, COMPOUND 12
21 (0.219) Cm (21-5x2.000)
QTof Premier HAB321
1: TOF MS ES+
586
512.0480
534.0302
536.0438
550.0369
552.0196
100
%
0
200
400
600
800
1000
1200
1400
1600
1800
2000
2200
m/z

Elemental Composition Report

Single Mass Analysis

Tolerance = 20.0 PPM / DBE: min = -1.5, max = 50.0
Element prediction: Off
Number of isotope peaks used for i-FIT = 3

Monoisotopic Mass, Even Electron Ions
1403 formula(e) evaluated with 31 results within limits (up to 50 best isotopic matches for each mass)
Elements Used:
C: 0-80 H: 0-50 N: 0-8 O: 0-8 S: 0-1 Cl: 0-1

Boulhaoua, COMPOUND 12 QTof Premier HAB321
43 (0.447) AM (Cen,4, 70.00, Ht,10000.0,556.28,0.70,LS 10); Cm (43:46) 1: TOF MS ES+ 2.26e+002

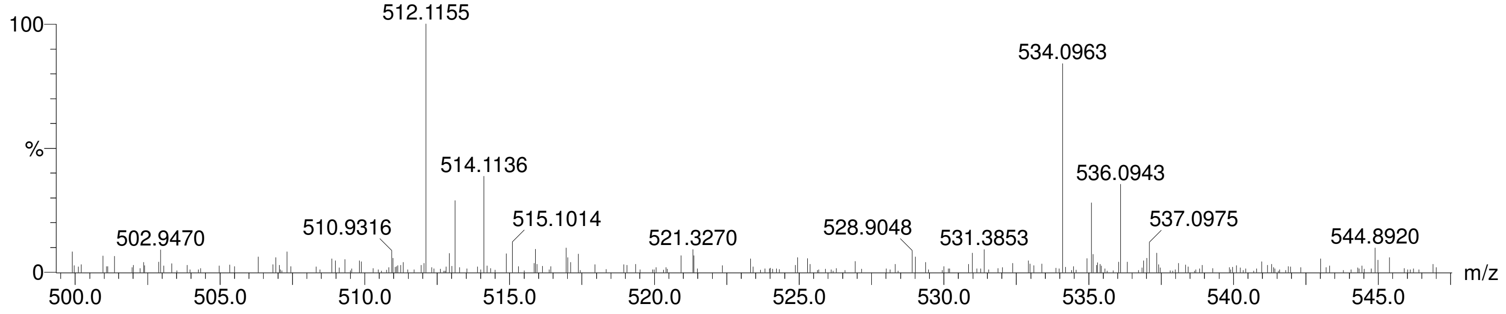

Mass	Calc. Mass	mDa	PPM	DBE	i-FIT	i-FIT (Norm)	Formula
Minimum:				-1.5			
Maximum:		5.0	20.0	50.0			
512.1155	512.1159	-0.4	-0.8	15.5	50.4	1.6	C24 H23 N5 O4 S Cl
	512.1146	0.9	1.8	10.5	50.5	1.7	C23 H27 N O8 S Cl
	512.1126	2.9	5.7	20.5	50.8	2.0	C27 H19 N5 O4 Cl
	512.1112	4.3	8.4	15.5	50.9	2.1	C26 H23 N O8 Cl
	512.1119	3.6	7.0	11.5	51.2	2.3	C19 H23 N7 O6 S Cl
	512.1166	-1.1	-2.1	24.5	51.6	2.8	C32 H19 N3 O2 Cl
	512.1200	-4.5	-8.8	19.5	51.8	3.0	C29 H23 N3 O2 S Cl
	512.1085	7.0	13.7	16.5	51.9	3.1	C22 H19 N7 O6 Cl
	512.1225	-7.0	-13.7	15.5	52.2	3.4	C25 H23 N3 O7 Cl
	512.1087	6.8	13.3	19.5	52.5	3.7	C30 H23 N O3 S Cl
	512.1238	-8.3	-16.2	20.5	52.9	4.1	C26 H19 N7 O3 Cl
	512.1060	9.5	18.6	20.5	53.3	4.4	C26 H19 N7 O S Cl
	512.1206	-5.1	-10.0	28.5	53.8	5.0	C37 H19 N Cl
	512.1053	10.2	19.9	24.5	54.4	5.6	C33 H19 N O3 Cl
	512.1240	-8.5	-16.6	23.5	54.5	5.7	C34 H23 N S Cl
	512.1141	1.4	2.7	20.5	56.3	7.5	C25 H18 N7 O4 S
	512.1128	2.7	5.3	15.5	56.4	7.6	C24 H22 N3 O8 S
	512.1168	-1.3	-2.5	19.5	56.7	7.9	C29 H22 N O6 S
	512.1181	-2.6	-5.1	24.5	57.0	8.2	C30 H18 N5 O2 S
	512.1109	4.6	9.0	28.5	57.2	8.4	C36 H18 N O S
	512.1069	8.6	16.8	24.5	58.3	9.4	C31 H18 N3 O3 S
	512.1134	2.1	4.1	24.5	58.7	9.9	C32 H18 N O6
	512.1107	4.8	9.4	25.5	58.7	9.9	C28 H14 N7 O4
	512.1221	-6.6	-12.9	28.5	58.7	9.9	C35 H18 N3 S
	512.1147	0.8	1.6	29.5	58.7	9.9	C33 H14 N5 O2
	512.1240	-8.5	-16.6	15.5	58.9	10.0	C23 H22 N5 O7 S
	512.1094	6.1	11.9	20.5	58.9	10.1	C27 H18 N3 O8
	512.1188	-3.3	-6.4	33.5	59.5	10.7	C38 H14 N3
	512.1206	-5.1	-10.0	20.5	59.6	10.8	C26 H18 N5 O7
	512.1075	8.0	15.6	33.5	59.6	10.8	C39 H14 N O
	512.1246	-9.1	-17.8	24.5	60.7	11.9	C31 H18 N3 O5

Boulhaoua, COMPOUND 13

QTof Premier HAB321

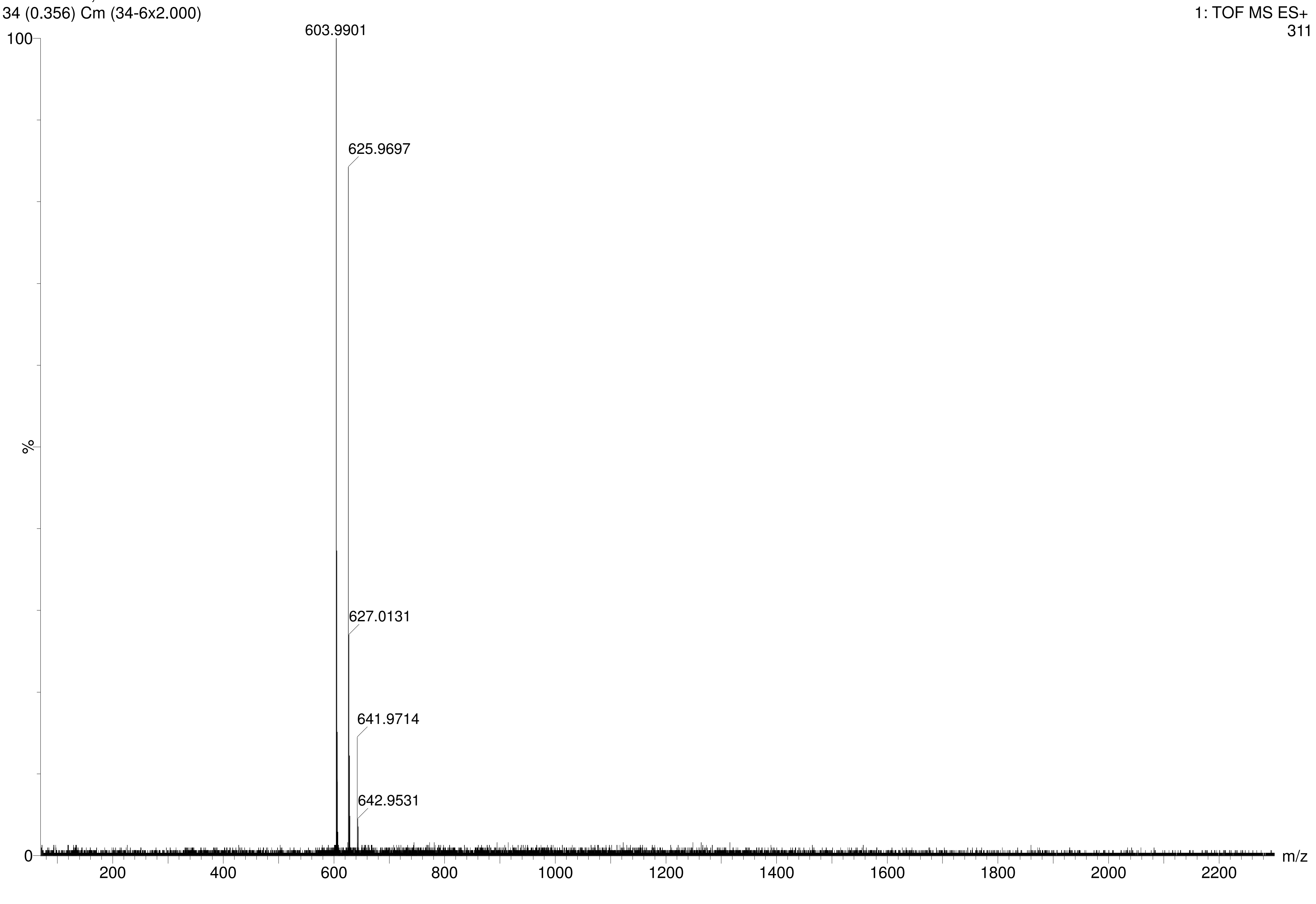

Single Mass Analysis

Tolerance = 20.0 PPM / DBE: min = -1.5, max = 50.0
Element prediction: Off
Number of isotope peaks used for i-FIT = 3

Monoisotopic Mass, Even Electron Ions
1403 formula(e) evaluated with 39 results within limits (up to 50 best isotopic matches for each mass)
Elements Used:
C: 0-80 H: 0-50 N: 0-8 O: 0-8 S: 0-1 I: 0-1

Boulhaoua, COMPOUND 13 QTof Premier HAB321
51 (0.528) AM (Cen,4, 70.00, Ht,10000.0,556.28,0.70,LS 10); Cm (46:52) 1: TOF MS ES+ 7.96e+002

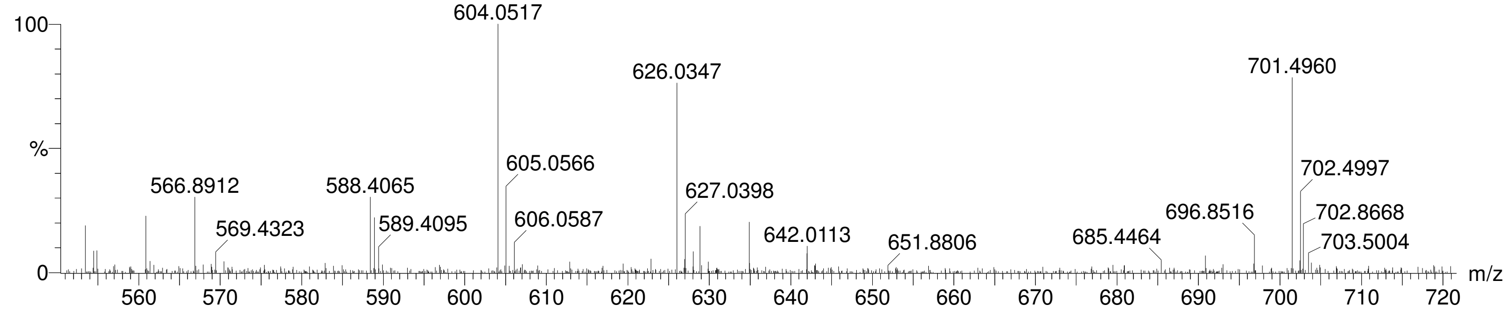

Mass	Calc. Mass	mDa	PPM	DBE	i-FIT	i-FIT (Norm)	Formula
Minimum:				-1.5			
Maximum:		5.0	20.0	50.0			
604.0517	604.0516	0.1	0.2	15.5	58.7	3.2	C24 H23 N5 O4 S I
	604.0522	-0.5	-0.8	24.5	59.1	3.6	C32 H19 N3 O2 I
	604.0511	0.6	1.0	44.5	60.7	5.2	C45 H6 N3 O
	604.0529	-1.2	-2.0	31.5	58.1	2.6	C33 H10 N5 O8
	604.0504	1.3	2.2	35.5	59.5	3.9	C37 H10 N5 O3 S
	604.0502	1.5	2.5	10.5	59.6	4.1	C23 H27 N O8 S I
	604.0491	2.6	4.3	30.5	58.7	3.2	C36 H14 N O7 S
	604.0545	-2.8	-4.6	39.5	60.8	5.3	C42 H10 N3 O S
	604.0482	3.5	5.8	20.5	60.4	4.9	C27 H19 N5 O4 I
	604.0556	-3.9	-6.5	19.5	57.0	1.5	C29 H23 N3 O2 S I
	604.0475	4.2	7.0	11.5	61.8	6.3	C19 H23 N7 O6 S I
	604.0562	-4.5	-7.4	28.5	59.7	4.2	C37 H19 N I
	604.0563	-4.6	-7.6	26.5	56.9	1.4	C30 H14 N5 O8 S
	604.0471	4.6	7.6	40.5	60.5	5.0	C40 H6 N5 O3
	604.0468	4.9	8.1	15.5	60.8	5.3	C26 H23 N O8 I
	604.0464	5.3	8.8	31.5	58.4	2.9	C32 H10 N7 O5 S
	604.0570	-5.3	-8.8	35.5	59.6	4.1	C38 H10 N3 O6
	604.0457	6.0	9.9	35.5	60.1	4.6	C39 H10 N O7
	604.0581	-6.4	-10.6	15.5	61.3	5.8	C25 H23 N3 O7 I
	604.0583	-6.6	-10.9	40.5	60.8	5.3	C39 H6 N7 O2
	604.0443	7.4	12.3	19.5	58.1	2.6	C30 H23 N O3 S I
	604.0442	7.5	12.4	16.5	62.8	7.3	C22 H19 N7 O6 I
	604.0594	-7.7	-12.7	20.5	61.6	6.1	C26 H19 N7 O3 I
	604.0596	-7.9	-13.1	23.5	58.5	3.0	C34 H23 N S I
	604.0432	8.5	14.1	39.5	62.8	7.3	C43 H10 N O2 S
	604.0603	-8.6	-14.2	30.5	59.7	4.2	C35 H14 N3 O6 S
	604.0430	8.7	14.4	36.5	60.9	5.4	C35 H6 N7 O5
	604.0610	-9.3	-15.4	39.5	62.1	6.6	C43 H10 N O4
	604.0614	-9.7	-16.1	10.5	61.5	5.9	C22 H27 N3 O7 S I
	604.0417	10.0	16.6	20.5	59.9	4.4	C26 H19 N7 O S I
	604.0617	-10.0	-16.6	35.5	61.1	5.6	C36 H10 N7 O2 S
	604.0621	-10.4	-17.2	19.5	61.7	6.1	C30 H23 N O5 I
	604.0623	-10.6	-17.5	44.5	63.1	7.6	C44 H6 N5
	604.0410	10.7	17.7	24.5	61.8	6.3	C33 H19 N O3 I
	604.0628	-11.1	-18.4	15.5	61.0	5.5	C23 H23 N7 O3 S I
	604.0405	11.2	18.5	40.5	63.1	7.5	C39 H6 N7 S
	604.0403	11.4	18.9	15.5	60.8	5.2	C25 H23 N3 O5 S I
	604.0634	-11.7	-19.4	24.5	62.3	6.8	C31 H19 N5 O I
	604.0399	11.8	19.5	44.5	63.9	8.4	C46 H6 N O2

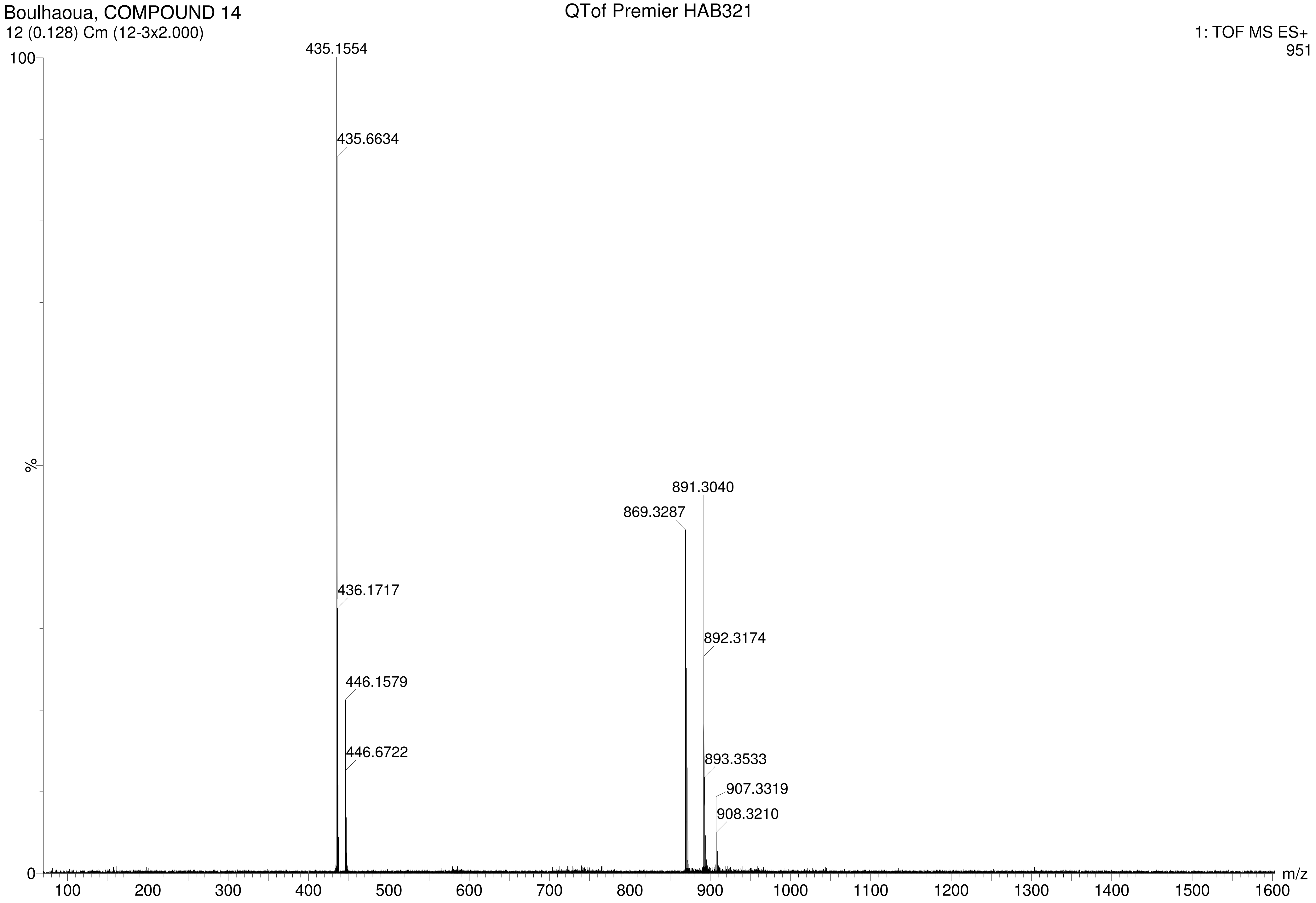

Boulhaoua, COMPOUND 14
12 (0.128) Cm (12-3x2.000)
QTof Premier HAB321
1: TOF MS ES+
951
435.1554
435.6634
436.1717
446.1579
446.6722
869.3287
891.3040
892.3174
893.3533
907.3319
908.3210
%
100
0
m/z
100
200
300
400
500
600
700
800
900
1000
1100
1200
1300
1400
1500
1600

Single Mass Analysis

Tolerance = 20.0 PPM / DBE: min = -1.5, max = 50.0
Element prediction: Off
Number of isotope peaks used for i-FIT = 3

Monoisotopic Mass, Even Electron Ions
702 formula(e) evaluated with 33 results within limits (up to 50 best isotopic matches for each mass)
Elements Used:
C: 0-80 H: 0-50 N: 0-8 O: 0-8 S: 0-1

Boulhaoua, COMPOUND 14 QTof Premier HAB321
17 (0.183) AM (Cen,4, 60.00, Ht,10000.0,556.28,0.70,LS 10); Cm (17:22) 1: TOF MS ES+ 6.60e+002

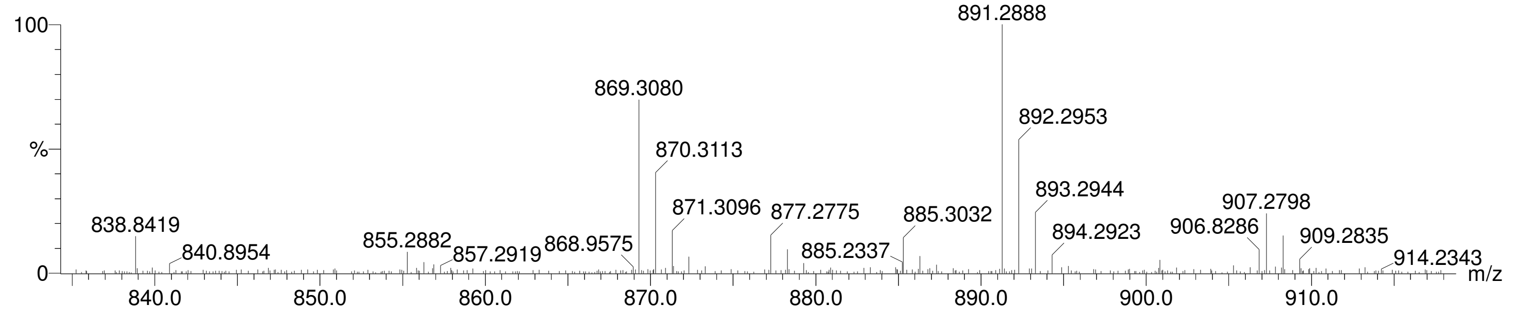

Minimum:				-1.5			
Maximum:		5.0	20.0	50.0			

Mass	Calc. Mass	mDa	PPM	DBE	i-FIT	i-FIT (Norm)	Formula
869.3080	869.3081	-0.1	-0.1	28.5	55.0	1.9	C46 H45 N8 O8 S
	869.3121	-4.1	-4.7	32.5	55.0	2.0	C51 H45 N6 O6 S
	869.3009	7.1	8.2	32.5	55.4	2.4	C52 H45 N4 O7 S
	869.3022	5.8	6.7	37.5	55.5	2.4	C53 H41 N8 O3 S
	869.3049	3.1	3.6	36.5	55.7	2.6	C57 H45 N2 O5 S
	869.3148	-6.8	-7.8	31.5	55.8	2.7	C55 H49 O8 S
	869.3063	1.7	2.0	41.5	56.1	3.1	C58 H41 N6 O S
	869.3088	-0.8	-0.9	37.5	56.2	3.1	C54 H41 N6 O6
	869.3114	-3.4	-3.9	36.5	56.4	3.3	C58 H45 O8
	869.3162	-8.2	-9.4	36.5	56.5	3.4	C56 H45 N4 O4 S
	869.3047	3.3	3.8	33.5	56.7	3.7	C49 H41 N8 O8
	869.3128	-4.8	-5.5	41.5	56.9	3.8	C59 H41 N4 O4
	869.3089	-0.9	-1.0	40.5	56.9	3.8	C62 H45 O3 S
	869.3015	6.5	7.5	41.5	57.0	3.9	C60 H41 N2 O5
	869.3029	5.1	5.9	46.5	57.2	4.2	C61 H37 N6 O
	869.3175	-9.5	-10.9	41.5	57.3	4.2	C57 H41 N8 S
	869.2989	9.1	10.5	42.5	57.3	4.3	C56 H37 N8 O3
	869.2975	10.5	12.1	37.5	57.4	4.3	C55 H41 N4 O7
	869.3056	2.4	2.8	45.5	57.5	4.4	C65 H41 O3
	869.3141	-6.1	-7.0	46.5	57.5	4.5	C60 H37 N8
	869.3234	-15.4	-17.7	32.5	57.5	4.5	C50 H45 N8 O5 S
	869.2937	14.3	16.4	36.5	58.0	5.0	C58 H45 O6 S
	869.2910	17.0	19.6	37.5	58.1	5.1	C54 H41 N6 O4 S
	869.3200	-12.0	-13.8	37.5	58.2	5.1	C53 H41 N8 O5
	869.2950	13.0	15.0	41.5	58.3	5.2	C59 H41 N4 O2 S
	869.3168	-8.8	-10.1	45.5	58.5	5.5	C64 H41 N2 O2
	869.2990	9.0	10.4	45.5	58.6	5.5	C64 H41 N2 S
	869.3227	-14.7	-16.9	36.5	58.7	5.6	C57 H45 N2 O7
	869.3202	-12.2	-14.0	40.5	58.7	5.7	C61 H45 N2 O2 S
	869.3240	-16.0	-18.4	41.5	59.3	6.2	C58 H41 N6 O3
	869.2917	16.3	18.8	46.5	59.6	6.6	C62 H37 N4 O2
	869.3208	-12.8	-14.7	49.5	60.5	7.4	C69 H41
	869.3242	-16.2	-18.6	44.5	60.9	7.9	C66 H45 S
